# Discovery of Molecular Glue Degraders via Isogenic Morphological Profiling

**DOI:** 10.1021/acschembio.3c00598

**Published:** 2023-11-21

**Authors:** Amanda Ng, Fabian Offensperger, Jose A. Cisneros, Natalie S. Scholes, Monika Malik, Ludovica Villanti, Andrea Rukavina, Evandro Ferrada, J. Thomas Hannich, Anna Koren, Stefan Kubicek, Giulio Superti-Furga, Georg E. Winter

**Affiliations:** † CeMM Research Center for Molecular Medicine of the Austrian Academy of Sciences, 1090 Vienna, Austria; ‡ Center for Physiology and Pharmacology, Medical University of Vienna, 1090 Vienna, Austria

## Abstract

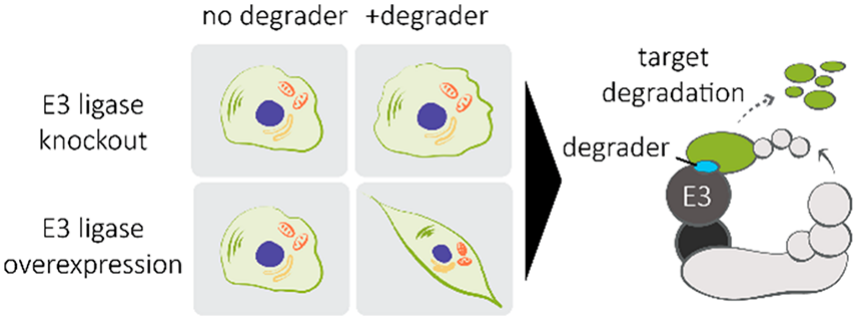

Molecular glue degraders (MGDs) are small molecules that degrade proteins of interest via the ubiquitin–proteasome system. While MGDs were historically discovered serendipitously, approaches for MGD discovery now include cell-viability-based drug screens or data mining of public transcriptomics and drug response datasets. These approaches, however, have target spaces restricted to the essential proteins. Here we develop a high-throughput workflow for MGD discovery that also reaches the nonessential proteome. This workflow begins with the rapid synthesis of a compound library by sulfur(VI) fluoride exchange chemistry coupled to a morphological profiling assay in isogenic cell lines that vary in levels of the E3 ligase CRBN. By comparing the morphological changes induced by compound treatment across the isogenic cell lines, we were able to identify **FL2-14** as a CRBN-dependent MGD targeting the nonessential protein GSPT2. We envision that this workflow would contribute to the discovery and characterization of MGDs that target a wider range of proteins.

## Introduction

Most small-molecule drugs antagonize or aggravate protein function. This narrow focus has limited the development of drugs against some of the most well-validated targets in disease and a myriad of other targets. Thus, we and others have begun to explore other pharmacological modulation strategies. One of these strategies is targeted protein degradation (TPD). Under the TPD strategy, small-molecule drugs recruit protein targets to E3 ubiquitin ligases for selective degradation.

There are two main classes of TPD drugs: proteolysis-targeting chimeras (PROTACs) and molecular glue degraders (MGDs). PROTACs comprise a target-binding warhead and an E3 ligase-binding warhead joined by a flexible linker. This modular design of PROTACs offers a rational strategy for targeting proteins of interest using existing ligands.^1,2^ Inherently, the degradable proteome explored is constrained to the ligandable proteome.^3−5^

In contrast to PROTACs, MGDs have a wider potential target space due to their molecular mechanism. MGDs induce cooperative interactions between E3 ligases and a target. The mechanistic elucidation of clinically approved thalidomide analogs as MGDs exemplifies how MGDs can expand the target space. Thalidomide analogs bind and stabilize the CRL4^CRBN^ E3 ligase complex with select zinc finger transcription factors. Consequently, these transcription factors are ubiquitinated and subsequently degraded.^6−8^ Other clinically tested compounds, such as indisulam, have also serendipitously been identified as MGDs (recruiting the E3 ligase DCAF15 to degrade the splicing factors RBM23 and RBM39).^9^ In both cases, MGDs prompt the degradation of proteins of interest without binding to them in isolation, thus highlighting the potential of MGDs in degrading unliganded proteins.^10,11^

The appeal of MGDs has led to the development of various screening approaches. Data mining of transcriptomics and drug response (drug sensitivity of cancer cell lines) resources, for example, has led to the identification of nondegradative molecular glues and MGDs.^12,13^ As these analyses use multiple cell lines, a wide range of essential targets can be explored. Given the intensive resources required, these analyses are typically performed retrospectively, thus precluding the analysis of custom-built chemical libraries. Target-agnostic primary assays employing readouts on cell viability have also been developed. These assays are less resource-intensive and enable the use of custom-built chemical libraries yet are still limited to essential target proteins.^14,15^

One attractive strategy to address this limitation is adapting a morphological profiling approach for MGD discovery. Morphological profiling approaches like the cell painting assay (CPA) monitor hundreds to thousands of parameters, including readouts on cell viability (by means of cell count). The CPA uses several dyes for the detection of cell organelles and components.^16,17^ It has also been used for detecting and distinguishing the bioactivities of many compound libraries.^18,19^ Currently, the CPA has largely been employed to interrogate the effects of genetic and pharmacologic perturbations within a single cell line context. We hypothesized that CPA could be adapted for MGD discovery by using isogenic cell line contexts. We refer to this approach as the isogenic CPA. In the isogenic CPA, we compare the morphological changes induced by compound treatment across cells expressing varying levels of an E3 ligase.

Here we report a proof of concept where we employ the isogenic CPA and the associated computational approach for discovering MGDs that co-opt CRBN (the substrate receptor of the CUL4^CRBN^ E3 ligase complex) for TPD. To test the isogenic CPA, we screened a library of 132 CRBN binders synthesized using a high-throughput synthesis protocol based on the sulfur(VI) fluoride exchange (SuFEx) chemistry that we developed. This new modality of click chemistry is based on the substitution of S(VI)–F with suitable nucleophiles forming stable chemical connections.^20^ The SuFEx reaction occurs under metal-free conditions, unlike the traditional copper-catalyzed azide–alkyne cycloaddition (CuAAC), making it ideal for subsequent cell-based high-throughput assays (“direct to biology” (D2B)). Using this workflow, we discovered and characterized a structurally novel degrader of GSPT2.

## Results

### Preparation of a Compound Library for the Isogenic CPA

We decided to test the isogenic CPA concept by focusing on the clinically validated E3 ligase CRBN. Compared to other E3 ligases, CRBN has the greatest number of reported MGDs and heterobifunctional degraders targeting various proteins (<50 unique targets),^21^ which we can utilize as CRBN-dependent controls for the isogenic CPA. Furthermore, CRBN has a permissive recognition motif on its targets known as a G-loop. Computational mining attempts have estimated that between 600 to over 2500 unique proteins contain a G-loop,^22−25^ thus potentially presenting a wide opportunity for the discovery of MGDs with novel targets. We here focus our efforts on RKO cells, a colorectal carcinoma cell line that we have previously employed to profile CRBN-based MGDs via deep mutational scanning.^26^ Notably, while half of the proteins predicted to contain G-loop(s) are not expressed in RKO, the remaining half largely comprise nonessential proteins (49.6%), with only 6.6% being essential proteins (Figure S1), highlighting the need for MGD discovery approaches that go beyond cell viability as a readout.

**Figure 1:**
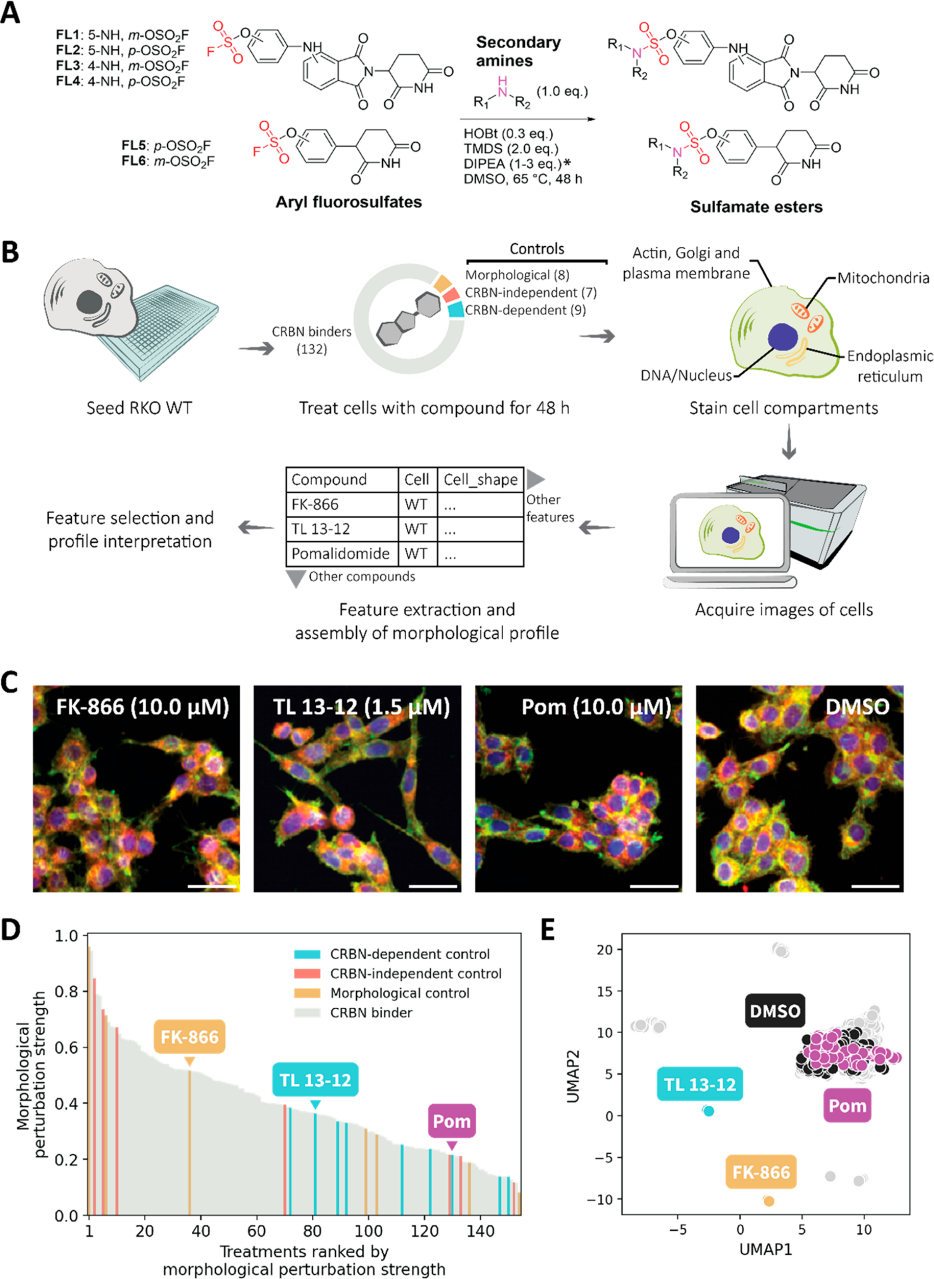
CRBN-dependent degraders have different morphological perturbation strengths. (A) Synthesis of sulfamate ester-based CRBN binders by SuFEx chemistry. *The number of equivalents of DIPEA used depends on the salt ratio of the corresponding amine. (B) Overview of the standard cell painting assay (CPA). (C) Images of cells treated with various compounds showing the diversity of morphological changes observed in CPA (scale bar: 50 μm). The images are composites of four fluorescent channels corresponding to the “Actin, Golgi and plasma membrane” (green), “DNA/Nucleus” (blue), “Endoplasmic reticulum” (yellow), and “Mitochondria” (red). (D) Morphological perturbation strengths of the compounds calculated using the cosine-similarity-metric-based robust Hellinger distance. (E) Projection of morphological profiles of compounds in two UMAP dimensions. Each point represents the median of the features extracted from all segmented cells per image (median of ∼200–300 cells per image).

For the design of our library, we used two different scaffolds: phthalimide and phenyl glutarimides. Phenyl glutarimides have been recently reported as alternative CRBN binders with high affinity,^27^ but their use has been limited to the synthesis of PROTACs. We utilize the SuFEx reaction, which is a highly efficient click chemistry reaction, making it ideal for high-throughput synthesis of a compound library. We first generated a series of fluorosulfates that would undergo SuFEx reactions. They can be easily obtained via fluorosulfonation of their corresponding phenol precursors as previously reported.^28^ Reacting these fluorosulfates with a collection of aliphatic amines would allow us to yield a library of structurally distinct sulfamates (Figure 1A). Of note, we could not isolate the aryl fluorosulfates in the *ortho* position from pomalidomide-based derivatives due to the formation of the corresponding cyclic sulfamates **12** and **13**. These cyclic sulfamates resulted from the reaction between the formed fluorosulfate and the adjacent aromatic NH (Scheme S1). We hypothesize that this reaction occurs assisted by proximity, since the poor nucleophilicity of the aromatic amine would typically prevent the formation of these molecules (in the absence of any catalyst/additive). Finally, sulfamate esters derived from **FL7** were not pursued given the very low affinity of the fluorosulfate for CRBN (EC_50_ = 72.4 μM). Altogether we were able to obtain six fluorosulfates (**FL1** to **FL6**; Figure 1A) with similar CRBN binding affinities to thalidomide (with EC_50_ ranging from 0.5 to 5.3 μM). We reacted these fluorosulfates with a collection of 21 amines (Table S1). Each compound is named based on the fluorosulfate precursor and the amine used (e.g., **FL2-14** is a product of the reaction between fluorosulfate **FL2** and amine **14**). To account for the different subproducts that can arise from reactions between different pairs of fluorosulfates and amines, we optimized the reaction conditions using a recently published protocol.^29^ In brief, aryl fluorosulfate molecules react with the appropriate secondary amines in the presence of TMDS, DIPEA, and catalytic amounts of HOBt at 65 °C for 48 h to yield the desired products in 384-well plate format. Conversion of the reactions was confirmed by thin-layer chromatography (TLC).

Using this protocol, we were able to rapidly synthesize a library of 132 compounds in 384-well plates that could be used directly in biological assays. To inform and benchmark the use of this library in CPA, we also selected nine CRBN-dependent bifunctional degraders and MGDs and six CRBN-independent compounds as controls (Table S3). We also included methylated pomalidomide (which lacks CRBN binding affinity) and eight compounds known to induce diverse morphological changes (dubbed “morphological controls”) recommended by the Joint Undertaking in Morphological Profiling-Cell Painting consortium.^30^

### CRBN-Dependent Degraders Have Different Morphological Perturbation Strengths

Prior to screening the compound library in the isogenic CPA, we assessed their CRBN binding affinities using a fluorescence-polarization-based competition assay with recombinant DDB1-CRBN and the fluorescent tracer **16** (Figure S2). The compounds from our library have EC_50_ values between 0.2 and 4.8 μM, which is in the range of thalidomide (EC_50_ = 1.0 μM). This observation indicates that modifications in the SuFEx handle can modulate the affinity for CRBN, in line with the reported influence of allostery in CRBN binding and the subsequent target recruitment and degradation;^31^ as such, we hereby refer to them as “CRBN binders”. We also assessed the cytotoxicity of the CRBN binders in RKO wild-type (WT) cells to determine the appropriate treatment concentration. As none of the CRBN binders were cytotoxic (with up to 13.5 μM tested; Figure S3), we decided to use the standard CPA treatment concentration of 10 μM.^16^

For the isogenic CPA, we used three isogenic cell lines expressing varying levels of CRBN: RKO WT, RKO with CRBN knocked out (RKO CRBN KO), and RKO CRBN KO reconstituted with CRBN (RKO CRBN OE). We first seeded the cells and left them to adhere to plates for 24 h before treating them with compounds for 48 h. Finally, we stained and fixed the cells and acquired images from the plates (Figure 1B).

We then assembled morphological profiles from these images. These profiles comprise robust *Z* scores of 3005 morphological features normalized to DMSO controls. Thereafter, feature selection was applied, and predictions/interpretations on compound bioactivity were made from the profiles (Figure 1B). We first explored the morphological profiles within the RKO WT context following a typical computational analysis for the CPA.^32−34^ We refer to this strategy as “global feature selection”, reflecting the way that the strategy is applied to the profiles of all compound treatments globally. For morphological profiles within the RKO WT context, a set of 470 features is selected by global feature selection. Various interpretations can then be made from the selected features of the profile, including the morphological perturbation strength, which is the magnitude of change across all morphological features induced by a compound compared to DMSO controls. We observed that our CRBN-dependent controls tended to induce mild to weak morphological changes in comparison to other controls such as the morphological control, FK-866 (Figure 1C–E). Echoing previously published data,^33^ some CRBN-dependent degraders, like pomalidomide, have low morphological perturbation strength, while others, like TL 13-12 (CRBN-dependent multikinase PROTAC;^35^Table S3), have a higher morphological perturbation strength. As visualized in a two-dimensional uniform manifold approximation and projection (UMAP) projection, many compounds induce subtle morphological changes within a confined spectrum of possible cellular morphologies (Figure 1E).^36^ Only a handful of compounds induce strong and distinct morphologies.

To distinguish these subtle changes and thereby distinguish compounds by their underlying bioactivities, the analysis of the CPA often employs large sets of reference compounds targeting a wide spectrum of biological targets. These reference compounds are used for annotating the bioactivity of test compounds by using a guilt-by-association strategy. Test compounds that induce similar morphological changes as reference compounds are predicted to have similar bioactivities to these reference compounds.^19,37^ The paucity of reported CRBN-dependent degraders (for use as reference compounds), however, precludes the use of this approach. We thus pursued a different strategy using multiple isogenic cell lines. We hypothesized that we could select features describing the CRBN-dependent bioactivities of compounds by examining the changes in features induced by the compounds across cell lines. In theory, if a compound has CRBN-dependent bioactivity, the magnitude of change in the feature induced by the compound (robust *Z* score) should correlate with the CRBN expression level of the cell line context. We refer to this strategy as “treatment-centric feature selection”. This treatment-centric feature selection would enable a CRBN-dependency prediction. We could also compare these treatment-centric features to distinguish the CRBN-dependent bioactivities of compounds and thereby prioritize compounds for target elucidation by the novelty of their bioactivity.

### Treatment-centric Feature Selection Enables CRBN Dependency Prediction

**Figure 2:**
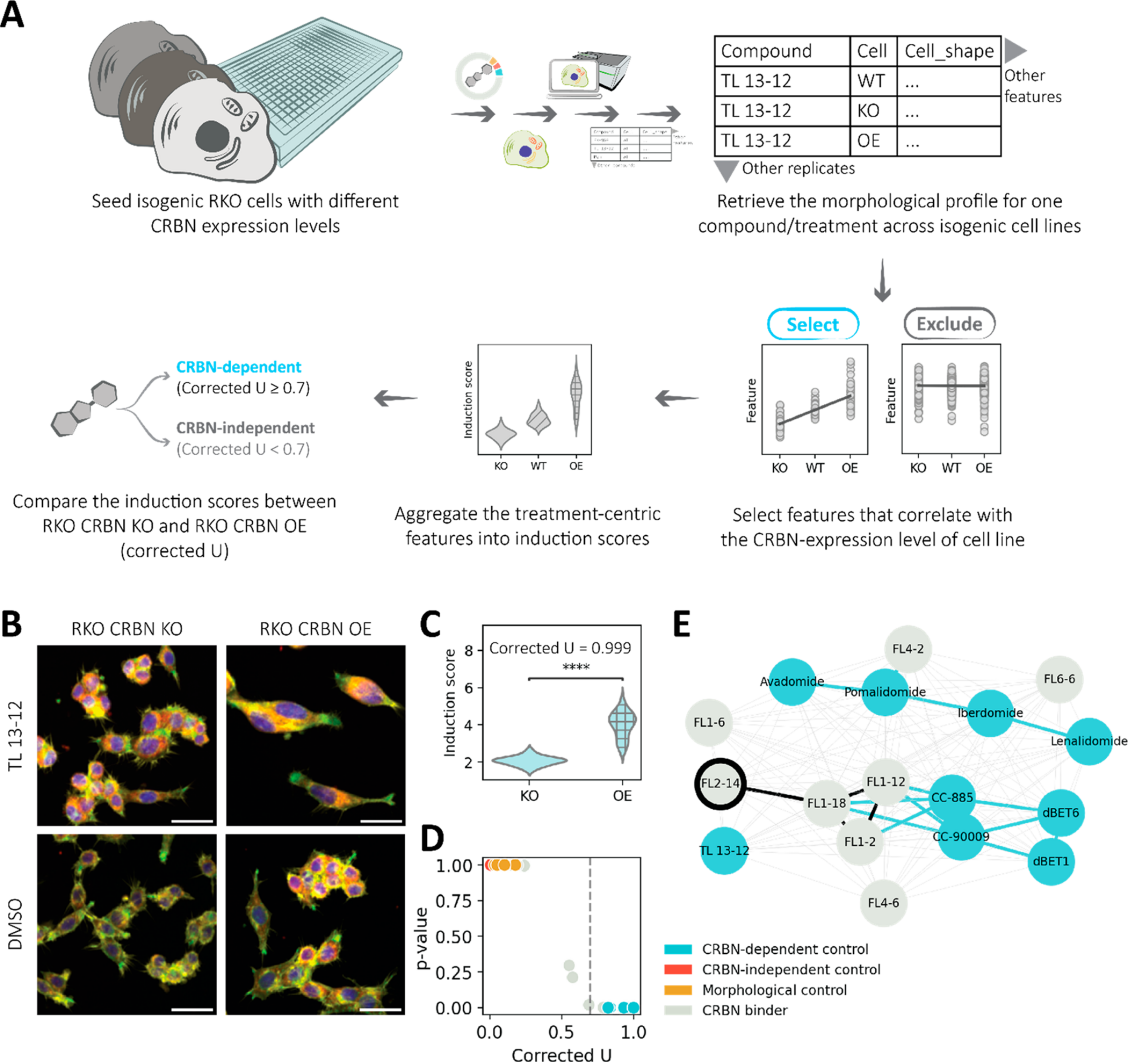
Treatment-centric feature selection enables CRBN-dependency prediction. (A) Overview of the isogenic CPA and usage of the morphological profiles assembled for treatment-centric feature selection. The isogenic CPA employs the use of multiple isogenic cell lines (three in this case, namely, RKO WT, RKO CRBN KO, and RKO CRBN OE). Like the standard CPA, morphological profiles are assembled. Thereafter, the morphological profile for a single compound treatment across cell line contexts is retrieved and analyzed using a treatment-centric feature selection approach that we developed. These treatment-centric features can be used to predict the likelihood of a compound having CRBN-dependent bioactivity. (B) Images of isogenic RKO cells treated with 1.5 μM TL 13-12 (scale bar: 50 μm). TL 13-12 enlarges cells drastically in a CRBN-dependent manner. (C) Induction scores calculated for TL 13-12 across isogenic RKO cells. The corrected *U* score predicts with high confidence (p < 1.00 × 10^–4^) that TL 13-12 has CRBN-dependent bioactivity. (D) The corrected *U* scores are calculated using the treatment-centric features of all compounds (with at least five treatment-centric features). The controls are distinguished clearly by corrected *U* score. (E) Compounds with predicted CRBN-dependent bioactivity can be grouped by treatment-centric features. The edges represent the similarity in treatment-centric features. Thin edges represent low similarity, while thick edges represent high similarity. Thick edges are further color-coded as black (both compounds connected by the edge are CRBN binders) and blue (at least one compound connected by the edge is a CRBN-dependent control). We selected **FL2-14** (circled in black) for target elucidation.

Putting our theorized treatment-centric feature selection into practice, we applied the following procedure. First, we retrieved the morphological profile for each compound treatment across the three isogenic cell lines (RKO WT, RKO CRBN KO, and RKO CRBN OE). We then selected features that exhibit a correlation between the robust *Z* score and the CRBN expression level of cell lines using Kendall’s τ_b_ correlation coefficient (Figure 2A). Cell morphology, however, is prone to noise; thus, we suspected that some features would correlate with CRBN expression levels of cell lines by chance. As we had suspected, a few CRBN-independent controls had treatment-centric features. In some cases, CRBN-independent controls had similar numbers of treatment-centric features as CRBN-dependent controls (Figure S4). We hypothesized that only compounds with true CRBN-dependent bioactivity would consistently exhibit a correlation between the robust *Z* score and the CRBN expression of cell lines across all observations. To account for the false selection of features, we began by averaging the robust *Z* scores across all treatment-centric features (dubbed the “induction score”) for each observation, i.e., each image of cells treated with the compound. Thereafter, we compared the induction scores calculated within the RKO CRBN KO and RKO CRBN OE contexts using the Mann–Whitney *U* test. The resulting score after correction for slight differences in the number of observations is the corrected *U* score (ranging from 0 to 1) (Figure 2A). Mathematically, any compound treatment that yields a corrected *U* score higher than 0.5 would be predicted to have CRBN-dependent bioactivity, and a higher corrected *U* score indicates a higher confidence in the prediction. To lower the risk of false predictions, we filtered for compound treatments with at least five treatment-centric features for calculating corrected *U* scores.

We first benchmarked this workflow with TL 13-12. We previously observed that TL 13-12 induces obvious morphological changes between the RKO CRBN KO and RKO CRBN OE contexts (Figure 2B). Most noticeably, TL 13-12 induced the drastic expansion of RKO CRBN OE and little to no changes in the size of RKO CRBN KO. The induction scores calculated for RKO CRBN OE are significantly higher than those for RKO CRBN KO (Figure 2C). The final corrected *U* score of 0.999 confidently predicts TL 13-12 as a compound with CRBN-dependent bioactivity. Expanding our procedure to all other compounds, we observed a distinction in our controls by the reported CRBN dependency (Figure 2D). Notably, all CRBN-dependent positive controls have corrected *U* scores above 0.7. We thus decided to apply this experimentally determined threshold of 0.7 for a compound to be predicted as CRBN-dependent. We also observed that the corrected *U* score has no correlation with the CRBN binding affinity or morphological perturbation strength of compounds (Figure S5A,B).

### Treatment-centric Features Can Group Compounds by CRBN-Dependent Bioactivity

Out of the 159 compounds we screened in the isogenic CPA, 17 compounds had at least five treatment-centric features and corrected *U* scores ≥0.7. Out of the 17, eight are CRBN binders from our library, and the remaining nine are CRBN-dependent controls (Table S4). To distinguish the CRBN-dependent bioactivities of the 17 compounds, we compared their treatment-centric features, which can vary dramatically. In our case, our 17 compounds with predicted CRBN-dependent bioactivity had anywhere between six and 267 treatment-centric features (with a median of 33.5 features and a standard deviation of 65.4 features). These large differences in treatment-centric features could reflect that the compounds modulate/degrade targets involved in various biological circuits. Comparing the treatment-centric features of each compound with corrected *U* scores ≥0.7 could hence allow us to distinguish compounds by their underlying CRBN-dependent bioactivity.

We carried out pairwise comparisons of compounds using the τ values of their treatment-centric features, where the similarity in the τ values was calculated using the cosine similarity metric. To visualize the cosine similarities, we plotted a force-directed network graph (Figure 2E). Pairs of compounds with thick edges have cosine similarities greater than 0.85. The thick edges are also color-coded if the compound has high cosine similarity to a CRBN-dependent control (blue) or a CRBN binder (black).

Using our CRBN-dependent controls as a gauge, we found that a cosine similarity of ≥0.85 indicated that our compounds had similar bioactivities. As TL 13-12 is our only control that degrades AURKA/PTK2, it is not connected to any other controls by thick edges. The degraders of IKZF1/3 and ZFP91 (avadomide, pomalidomide, iberdomide, and lenalidomide) are only connected to one another. Interestingly, the degraders of GSPT1 (CC-885 and CC-90009) are connected to the degraders of the BET family proteins (dBET1 and dBET6). We postulate that these edges may be due to the similarity in cytotoxicity induced by these compounds in RKO WT and RKO CRBN OE. Notably, CC-885 has similar treatment-centric features to three CRBN binders, **FL1-2**, **FL1-12**, and **FL1-18**. In contrast, dBET1 and dBET6 do not have similar treatment-centric features to these CRBN binders. Accordingly, the CC-885/CC-90009 pair clusters closer together on the network graph than the dBET1/dBET6 pair and vice versa. This observation implies that the cosine similarities a compound has to other compounds also play a key role in distinguishing the bioactivities of compounds in addition to the treatment-centric features of the compound.

### FL2-14 Is a CRBN-Dependent GSPT2 Molecular Glue Degrader

**Figure 3:**
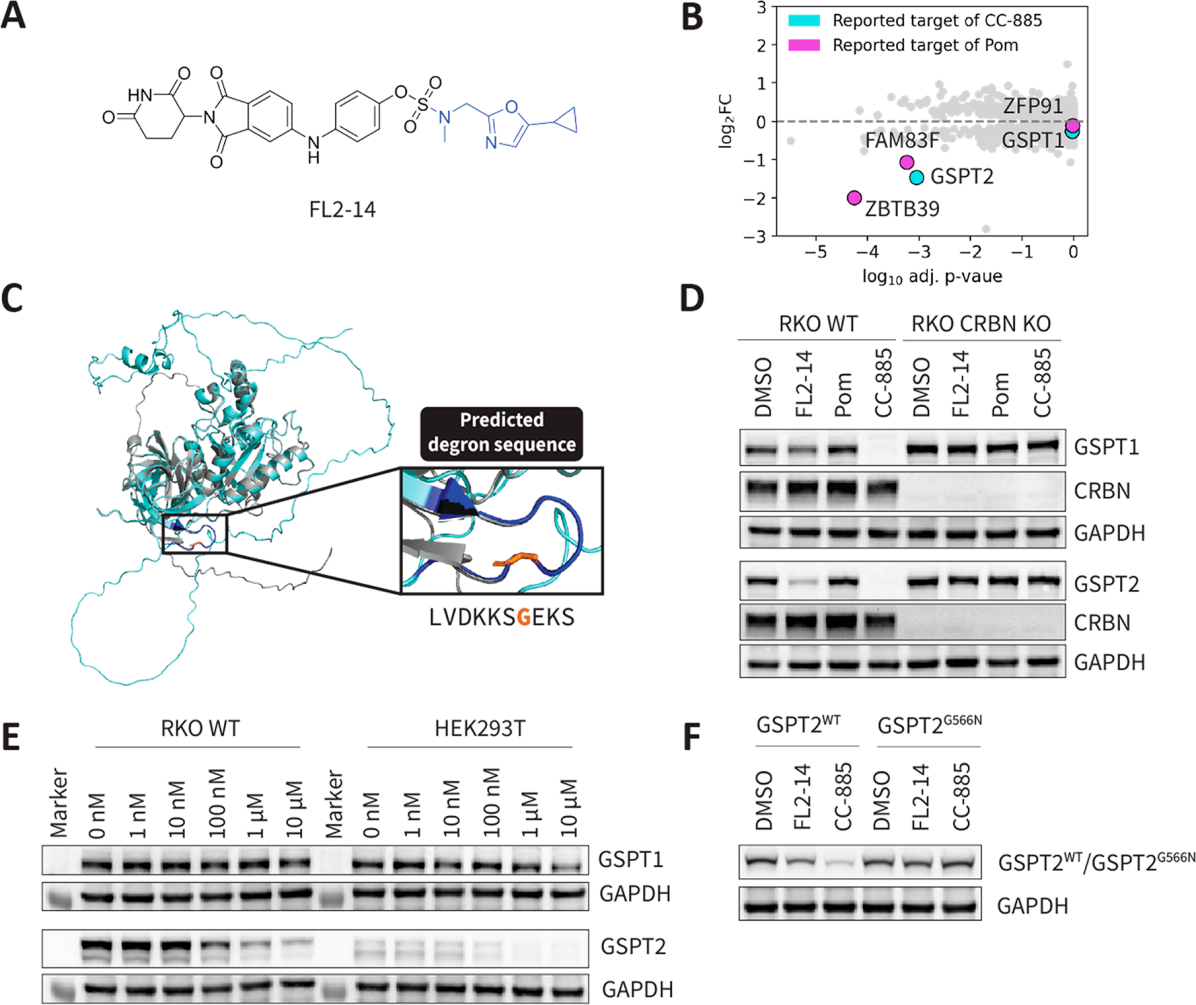
**FL2-14** is a selective GSPT2 molecular glue degrader. (A) Structure of **FL2-14** comprising fluorosulfate scaffold **FL2** (black) and tail group modification **14** (blue). (B) DMSO-normalized quantitative proteomics after 16 h of treatment with **FL2-14** (10 μM) in RKO WT (*n* = 2). **FL2-14** significantly downregulates (fold change > −2.00 and adjusted *p* < 0.01) some of the reported targets of CC-885 and pomalidomide (Pom). (C) Structural alignment of AlphaFold models of GSPT1 (AF-P15170-F1-model_v4 in gray) and GSPT2 (AF-Q8IYD1-F1-model_v4 in blue). (D) Validation of GSPT2 degradation by **FL2-14**. RKO WT or CRBN KO cells were treated for 16 h with 10 μM **FL2-14**, 10 μM Pom, or 10 nM CC-885 and subjected to Western blot analysis. (E) Dose–response of **FL2-14** in RKO and HEK293T cells. Cells were treated with the **FL2-14** (0 to 10 μM) for 16 h. (F) Degradation of GSPT2^WT^ or GSPT2^G566N^. RKO cells stably overexpressing HA-tagged GSPT2^WT^ or GSPT2^G566N^ were treated with 10 μM **FL2-14** or 10 nM CC-885 for 16 h and subjected to Western blot analysis for HA-tag detection.

We selected **FL2-14** for target elucidation (circled in black in Figure 2E) based on three criteria (Table S4). First, **FL2-14** has low cosine similarity with all of the CRBN-dependent controls (i.e., no thick blue edges). Second, it has comparable CRBN binding affinity to thalidomide, confirming sufficient E3 ligase recruitment. Third, it has a unique chemical structure, being the only CRBN binder with the tail group modification **14** predicted to have CRBN-dependent bioactivity (Figure 3A). To elucidate the target for **FL2-14**, we performed quantitative proteomics to evaluate changes in protein abundances upon treatment with **FL2-14**. Over 8000 proteins were quantified, of which three proteins were significantly downregulated, namely, GSPT2, FAM83F, and ZBTB39 (Figure 3B). There is a partial overlap in the downregulated targets of **FL2-14** with two CRBN-dependent controls (CC-885 and pomalidomide). CC-885 degrades both GSPT1 and GSPT2 among other targets. Although GSPT1 and GSPT2 are homologous proteins, GSPT1 is pan-essential while GSPT2 is not essential in any of the 1095 assayed cell lines (according to https://depmap.org/portal/). This target difference corroborates the previous observation that **FL2-14** is not cytotoxic (Figure S3) and supports the low similarity between the treatment-centric features of **FL2-14** and CC-885 (Figure 2E). **FL2-14** also shares two common targets with pomalidomide: ZBTB39 and FAM83F. Pomalidomide is also not cytotoxic to RKO WT, considering that none of its targets expressed in RKO WT are essential (Table S3). The low similarity in treatment-centric features observed may thus be driven by the difference in downregulation of other targets of pomalidomide, such as ZFP91.

Given that GSPT2 and GSPT1 are closely related and that both are functionally involved in translation termination, we were intrigued by the apparent preferential degradation of GSPT2 by **FL2-14**. Comparing the AlphaFold models of GSPT1 and GSPT2, GSPT2 has an extended disordered C-terminal domain but otherwise shares the same structure and predicted G-loop sequence as GSPT1 (Figure 3C). Corroborating quantitative proteomics, **FL2-14** indeed preferentially degraded GSPT2 over GSPT1 as revealed by Western blot analysis (Figure 3D). In line with the predicted function as a CRBN modulator, GSPT2 degradation by **FL2-14** was completely abrogated in RKO CRBN KO cells (Figure 3D). GSPT2 degradation was observed in the submicromolar range, could also be observed in HEK293T cells (Figure 3E), and was apparent after 5 h of **FL2-14** treatment (Figure S6A). Moreover, **FL2-14** did not induce degradation of IKZF1, the consensus target of thalidomide analogs (Figure S6B). As expected, substitution of the glycine in the G-loop of GSPT2 with asparagine rescued degradation of GSPT2 through **FL2-14** or CC-885 (Figure 3F), confirming the predicted G-loop of GSPT2.

Next, we wondered whether other CRBN binders with tail group modification **14** could degrade GSPT1/2. Aside from **FL2-14** (and to a lesser extent **FL1-14**), none of the other CRBN binders degraded GSPT1/2 (Figure S6C). **FL1-14** was not predicted to have CRBN-dependent bioactivity, as it had no treatment-centric features selected, following which no corrected *U* score could be calculated. The absence of treatment-centric features could suggest that the weak degradation of GSPT2 was insufficient for inducing a quantifiable morphological change, unlike **FL2-14**. We also observed that **FL2-14** has high similarity in treatment-centric features with **FL1-18**, which in turn has high similarity in treatment-centric features with **FL1-2** and **FL1-12** (Figure 2E). These CRBN binders exhibited a similar preference in degrading GSPT2 over GSPT1 in a CRBN-dependent manner (Figure S6D,E), validating the computational prediction that these CRBN binders have CRBN-dependent bioactivity. Similar to **FL2-14**, these CRBN binders are not cytotoxic in RKO WT and HEK293T (EC_50_ not reached at 10 μM treatment; Figure S6F). Additionally, the tendency of these GSPT2 degraders to cluster on the network graph suggests that the treatment-centric features can also be indicative of the underlying biological targets. In summary, our computational approach was able to identify **FL2-14** as a CRBN-dependent degrader that preferentially degrades the nonessential GSPT2 over the closely related GSPT1.

## Conclusion

MGDs are small-molecule drugs that hold promise in targeting otherwise unligandable proteins but remain challenging to discover. Current cell-based approaches to discover MGDs largely rely on screening compound libraries through primary assays reliant on cell viability as a readout or data mining of public transcriptomic and drug response datasets. Both approaches focus on essential targets, which represent only a fraction of all expressed or therapeutically relevant proteins of the assayed cell line system. Exploring the nonessential proteome could hence greatly extend the target reach of an MGD discovery workflow.

To this end, we developed and benchmarked the isogenic CPA. We also used the isogenic CPA to identify functionally differentiated, CRBN-based MGDs from a bespoke library of CRBN-binding ligands. By comparing the morphological features changed by compound treatments across isogenic cell lines expressing different levels of CRBN (referred to as “treatment-centric features”), we successfully segregated CRBN-dependent from CRBN-independent controls. Based on comparative analysis of treatment-centric features, we selected **FL2-14** for further validation. We identified and validated GSPT2 as a target of **FL2-14**, which is degraded in a CRBN-dependent manner. Interestingly, **FL2-14** featured selectivity in degrading GSPT2 over the closely related GSPT1, which is a known off-target liability of CRBN-based PROTACs.^38^ GSPT2 was identified as a novel termination release factor more than two decades ago.^39^ In humans, GSPT1 (eRF3a) and GSPT2 (eRF3b) share almost 88% identity. Both homologues are also expressed in most human tissues, with the GSPT2 level generally being lower than that of GSPT1.^40^ GSPT1 is considered the major factor acting in translation termination, and there is evidence that GSPT2 can partially substitute GSPT1.^40^ The role of GSPT2, however, has been poorly characterized thus far; FL2-14 might serve as a useful chemical probe for disentangling the role of GSPT2 (from GSPT1).

Intriguingly, the CRBN-dependent controls tended to cluster loosely either by their degradation targets or based on target essentiality. This observation could indicate that the network graph of treatment-centric features could be used to cluster compounds by their degradation targets, although a more extensive study of CRBN-dependent MGDs with known targets would be required to test this hypothesis. Collectively, we expect the logic behind the developed isogenic CPA to be applicable to all other E3 ligases that are amenable to gain and loss of function mutations in a given cellular background. Of note, this approach could be expanded toward regulators that orchestrate the activity of entire clades of E3 ligases, as we have done before.^14^ In summary, we anticipate that these efforts will expand the reach of MGDs toward nonessential targets of therapeutic relevance in fields other than oncology.

Crucially, the presented experimental and analytical strategy could be adapted to identify proximity-inducing compounds outside the field of targeted protein degradation, which is an active area of research.^41,42^ Here we focus on targeted protein degradation. Following our focus, we employed quantitative proteomics for the initial target/mechanistic elucidation for hit compounds identified by the isogenic CPA. If other pharmacological modulation strategies are being pursued, we expect that the isogenic CPA will remain a useful blueprint for hit compound selection and prioritization. The key change required would be the subsequent target and mechanistic elucidation approach used.

Overall, we believe that the isogenic CPA and the computational analyses we have developed will contribute to the arsenal of primary assays for MGD discovery.

## Data Availability

The mass spectrometry proteomics data have been deposited to the ProteomeXchange Consortium via the PRIDE partner repository^43^ with the dataset identifier PXD045892. The computational pipeline for the isogenic CPA can be found at https://github.com/GWinterLab/IsogenicCPA.

## Supporting Information Available

Materials and methods including chemical synthesis information, fluorescence polarization-based competition assay results, CellTiter-Glo assay results, additional information on the controls used in the isogenic CPA, additional plots from the isogenic CPA, and Western blots (PDF)
Compound calculations from the isogenic CPA (XLSX)
