# Supplementary material for: Discovery of Molecular Glue Degraders via Isogenic Morphological Profiling
Source: ACS Chem Biol. 2023 Nov 21;18(12):2464–73. doi: 10.1021/acschembio.3c00598 (PMC10764104; doi:10.1021/acschembio.3c00598)
Supplement: Supplementary file 1 — cb3c00598_si_001.pdf [file cb3c00598_si_001.pdf]

Discovery of molecular glue degraders via isogenic morphological profiling

Amanda Ng[1,#], Fabian Offensperger[1,#], Jose A. Cisneros[1,#], Natalie S. Scholes[1], Monika Malik[1], Ludovica Villanti[1], Andrea Rukavina[1], Evandro Ferrada[1], J. Thomas Hannich[1], Anna Koren[1], Stefan Kubicek[1], Giulio Superti-Furga[1, 2], Georg E. Winter[1,*]

[1]CeMM, Research Center for Molecular Medicine of the Austrian Academy of Sciences, 1090, Vienna, Austria.

[2]Center for Physiology and Pharmacology, Medical University of Vienna, 1090, Vienna, Austria

[#]equal contribution

[*]correspondence to gwinter@cemm.oeaw.ac.at

Supporting Information

Contents

General information on chemical synthesis 3

Synthesis of fluorosulfates FL1 to FL7 and cyclic sulfamates 12, 13....................3

General procedure for the synthesis of 1, 2 3

General procedure for the synthesis of 3-5....................4

General procedure for the synthesis of phenols 6-11 4

General procedure for the synthesis of fluorosulfates FL1 to FL4 5

General procedure for the synthesis of fluorosulfates FL5, FL6 and FL7 6

Synthesis of cyclic sulfamates 12, 13....................6

High Throughput Synthesis of sulfamate esters....................7

Synthesis of FL2-14 8

Synthesis of fluorescent tracer 16 8

^{1}H, ^{13}C and ^{19}F NMR spectra 10

Experimental methods 22

Assessment of gene expression and essentiality in RKO WT....................22

Cell culture....................22

Fluorescence Polarization-based competition assay....................22

Cell-titer Glo assay....................22

Isogenic Cell Painting Assay....................23

Expression proteomics to identify the degradation target of FL2-14....................26

Analysis of targeted protein degradation of GSPT1 and GSPT2 via western blot 28

Prediction of the G-loop degron sequence in GSPT2 29

Analysis of G-loop mutation 29

Expression and purification of recombinant CRBN-DDB1 complex....................30

Supplementary references 37

General information on chemical synthesis

All starting materials, solvents, and reagents used were purchased from commercial sources unless stated otherwise, with no further purification. Reactions were monitored by thin-layer chromatography (TLC) using pre-coated silica gel plates F-254. Proton nuclear magnetic resonance (^{1}H NMR) spectra, carbon nuclear magnetic resonance (^{13}C NMR) spectra and fluorine nuclear magnetic resonance (^{19}F NMR) spectra were recorded on Bruker AV Neo 500, AV III 600 and AV III HD 700 instruments from the NMR Facility of the University of Vienna. The NMR peak multiplicities are denoted as follows: s, singlet; d, doublet; dd, doublet of doblets: t, triplet; m, multiplet; br s, broad singlet. Column chromatography was carried out using Biotage Selekt over Biotage Sfar Silica D column cartridges employing Merck silica gel (Kieselgel 60, 63-200 μm). Low resolution mass determinations were performed using electrospray ionization (ESI) on Bruker amaZon speed ETD while HRMS (ESI-TOF) analyses were performed on Bruker timsTOF flex at the MS Facility of the University of Vienna. 384-well plates with lid were purchased from Corning. Library of secondary amines was purchased from provided by Enamine and Sigma.

Synthesis of fluorosulfates FL1 to FL7 and cyclic sulfamates 12, 13

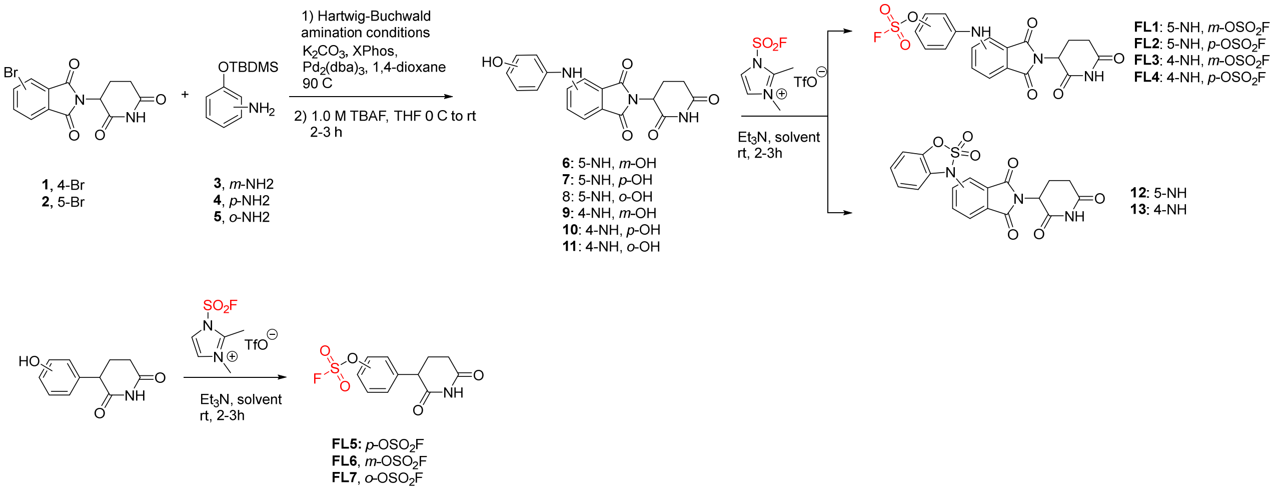

Scheme S 1: Synthesis of aryl fluorosulfates and cyclic sulfamates.

General procedure for the synthesis of 1, 2

A mixture of 3-bromophthalic or 4-bromophtalic anhydride (200 mg, 0.88 mmol), 3-aminopiperidine-2, 6-dione hydrochloride (144 mg, 0.88 mmol) and sodium acetate (180 mg, 2.2 mmol) in acetic acid (4.4 mL) is stirred at 120 C for 16 h. Once the reaction is completed, the mixture is cooled, water is added, and the desired product collected by filtration. Characterization data correspond to those previously reported.[1]

5-bromo-2-(2,6-dioxopiperidin-3-yl)isoindoline-1,3-dione (1). 260 mg. Yield 80%. ^{1}H NMR (600 MHz, DMSO) δ 11.14 (s, 1H), 8.15 (d, J = 1.6 Hz, 1H), 8.10 (dd, J = 7.9, 1.7 Hz, 1H), 7.87 (d, J = 7.9 Hz, 1H), 5.16 (dd, J = 13.0, 5.4 Hz, 1H), 2.95 – 2.83 (m, 1H), 2.65 – 2.58 (m, 1H), 2.56 – 2.51 (m, 1H), 2.10 – 2.02 (m, 1H). (ESI) $[M + H]^+$ calcd for $C_{13}H_{11}N_2O_4Br$, 336.98; found, 336.98

4-bromo-2-(2,6-dioxopiperidin-3-yl)isoindoline-1,3-dione (2). 259 mg. Yield 77%. ^{1}H NMR (600 MHz, DMSO) δ 11.15 (s, 1H), 8.07 (dd, J = 8.1, 0.8 Hz, 1H), 7.93 (dd, J = 7.3, 0.8 Hz, 1H), 7.78 (dd, J = 8.1, 7.4 Hz, 1H), 5.17 (dd, J = 12.9, 5.4 Hz, 1H), 2.95 – 2.85 (m, 1H), 2.66 – 2.57 (m, 1H), 2.56 – 2.51 (m, 1H), 2.11 – 2.03 (m, 1H). (ESI) $[M + H]^+$ calcd for $C_{13}H_{11}N_2O_4Br$, 336.98; found, 336.98

General procedure for the synthesis of 3-5

Imidazole (306 mg, 4.5 mmol) is added to a solution of *o*-aminphenol, *m*-aminophenol or *p*-aminophenol (200 mg, 1.8 mmol) in THF (3.0 mL). TBDMS chloride (406 mg, 2.7 mmol) is added to the mixture at 0 °C and warmed to room temperature. The reaction is stirred for 5-6 h until TLC shows no starting aminophenols. The reaction is diluted with ethyl acetate and washed with water and brine. The organic layer was dried over anhydrous Na_2SO_4, filtered, the solvent evaporated under reduced pressure and the desired intermediates purified by flash chromatography in hexane:ethyl acetate. Compounds obtained appear as dark oils. Characterization data correspond to those previously reported.[2]

3-((tert-butyldimethylsilyl)oxy)aniline (3). 360 mg. Yield 90%. ^{1}H NMR (600 MHz, $CDCl_3$) δ 7.00 (t, *J* = 8.0 Hz, 1H), 6.36 – 6.31 (m, 1H), 6.31 – 6.26 (m, 1H), 6.25 – 6.20 (m, 1H), 0.97 (s, 9H), 0.19 (s, 6H).

4-((tert-butyldimethylsilyl)oxy)aniline (4). 308 mg. Yield 75%. ^{1}H NMR (600 MHz, $CDCl_3$) δ 6.67 – 6.64 (m, 2H), 6.59 – 6.56 (m, 2H), 3.40 (s, 2H), 0.96 (s, 10H), 0.15 (s, 6H).

2-((tert-butyldimethylsilyl)oxy)aniline (5). 350 mg. Yield 86%. ^{1}H NMR (600 MHz, $CDCl_3$) δ 6.81 – 6.76 (m, 1H), 6.76 – 6.71 (m, 2H), 6.65 – 6.60 (m, 1H), 1.02 (s, 9H), 0.24 (s, 6H).

General procedure for the synthesis of phenols 6-11

To a mixture of **1** or **2** (150 mg, 0.44 mmol) and corresponding anilines **3**, **4** or **5** (98 mg, 0.44 mmol) in 1,4-dioxane (0.3 M), Pd2(dba)3 (20 mg, 0.022 mmol), XPhos (41 mg, 0.088 mmol) and K2CO3 (121 mg, 0.88 mmol) are added. After three cycles of argon and vacuum, the reaction is stirred at 90 C for 16 h. After solvent evaporation under reduced pressure, the mixture is redissolved in ethyl acetate and washed with water, aq. 0.5 M HCl and brine and take for TBDMS deprotection without further purification. The resulting mixture is dissolved in THF and 1.0 M TBAF in THF is added at 0 C. The reaction is allowed to warm to room temperature and stirred for 2-3 h. Ethyl acetate is added and washed with water. Organic layer is dried over anhydrous Na_2SO_4, filtered and solvent evaporated under reduced pressure. Desired phenols were purified by flash chromatography in dichloromethane:methanol and obtained as yellow powders.

2-(2,6-dioxopiperidin-3-yl)-5-((3-hydroxyphenyl)amino)isoindoline-1,3-dione (6). 120 mg. Yield 75%. ^{1}H NMR (700 MHz, DMSO) δ 11.08 (s, 1H), 9.52 (s, 1H), 9.12 (s, 1H), 7.70 (d, *J* = 8.3 Hz, 1H), 7.33 (d, *J* = 2.1 Hz, 1H), 7.28 (dd, *J* = 8.3, 2.1 Hz, 1H), 7.16 (t, *J* = 7.9 Hz, 1H), 6.70 – 6.61 (m, 2H), 6.53 – 6.43 (m, 1H), 5.07 (dd, *J* = 12.9, 5.5 Hz, 1H), 2.94 – 2.82 (m, 1H), 2.61 – 2.56 (m, 1H), 2.56 – 2.50 (m, 1H), 2.05 – 1.99 (m, 1H). ^{13}C NMR (176 MHz, DMSO) δ 172.8, 170.0, 167.3, 166.8, 158.4, 150.5, 141.4, 133.9, 130.3, 125.3, 119.2, 118.6, 111.0, 110.4, 107.9, 107.0, 48.8, 30.9, 22.1. HRMS (ESI) $[M + Na]^+$ calcd for C19H15N3O5, 365.1012; found, 388.0912

2-(2,6-dioxopiperidin-3-yl)-5-((4-hydroxyphenyl)amino)isoindoline-1,3-dione (7). 112 mg. Yield 70%. Characterization data correspond to those previously reported.[3] ^{1}H NMR (500 MHz, DMSO) δ 11.06 (s, 1H), 9.40 (s, 1H), 8.88 (s, 1H), 7.62 (d, *J* = 8.2 Hz, 1H), 7.10 – 7.00 (m, 4H), 6.85 – 6.75 (m, 2H), 5.04 (dd, *J* = 12.7, 5.4 Hz, 1H), 2.93 – 2.82 (m, 1H), 2.64 – 2.52 (m, 2H), 2.03 – 1.97 (m, 1H). HRMS (ESI) $[M + H]^+$ calcd for C19H15N3O5, 365.1012; found, 366.1086.

2-(2,6-dioxopiperidin-3-yl)-5-((2-hydroxyphenyl)amino)isoindoline-1,3-dione (8). 110 mg. Yield 69%. ^{1}H NMR (600 MHz, DMSO) δ 11.06 (s, 1H), 9.71 (s, 1H), 8.65 (s, 1H), 7.63 (d, *J* = 8.3 Hz, 1H), 7.20 (dd, *J* = 7.8, 1.6 Hz, 1H), 7.12 – 7.01 (m, 3H), 6.96 (dd, *J* = 8.1, 1.5 Hz, 1H), 6.85 (td, *J* = 7.6, 1.5 Hz, 1H), 5.04 (dd, *J* = 12.9, 5.4 Hz, 1H), 2.96 – 2.79 (m, 1H), 2.63 – 2.52 (m, 2H), 2.04 – 1.99 (m, 1H). ^{13}C NMR (176 MHz, DMSO) δ 172.8, 170.1, 167.5, 167.0, 152.1, 151.1,

133.7, 126.9, 125.8, 124.9, 124.7, 119.4, 117.9, 117.6, 116.4, 107.3, 48.7, 30.9, 22.2. HRMS (ESI) $[M + Na]^+$ calcd for C19H15N3O5, 365.1012; found, 388.0899

2-(2,6-dioxopiperidin-3-yl)-4-((3-hydroxyphenyl)amino)isoindoline-1,3-dione (9). 112 mg. Yield 70%. ^{1}H NMR (600 MHz, DMSO) δ 11.13 (s, 1H), 9.55 (s, 1H), 8.32 (s, 1H), 7.67 – 7.59 (m, 1H), 7.47 (d, *J* = 8.6 Hz, 1H), 7.29 – 7.22 (m, 1H), 7.17 (t, *J* = 8.0 Hz, 1H), 6.80 – 6.69 (m, 2H), 6.59 – 6.50 (m, 1H), 5.12 (dd, *J* = 12.8, 5.5 Hz, 1H), 2.98 – 2.82 (m, 1H), 2.67 – 2.52 (m, 2H), 2.13 – 2.03 (m, 1H). ^{13}C NMR (151 MHz, DMSO) δ 172.8, 170.4, 168.3, 167.1, 158.3, 150.5, 142.8, 136.2, 132.4, 130.2, 119.7, 113.4, 112.3, 111.9, 111.2, 108.5, 48.7, 31.0, 22.1. HRMS (ESI) $[M + Na]^+$ calcd for C19H15N3O5, 365.1012; found, 388.0894

2-(2,6-dioxopiperidin-3-yl)-4-((4-hydroxyphenyl)amino)isoindoline-1,3-dione (10). 122 mg. Yield 76%. Characterization data correspond to those previously reported.[3] ^{1}H NMR (600 MHz, DMSO) δ 11.11 (s, 1H), 9.45 (s, 1H), 8.13 (s, 1H), 7.57 – 7.49 (m, 1H), 7.18 – 7.11 (m, 3H), 7.11 – 7.04 (m, 1H), 6.86 – 6.76 (m, 2H), 5.11 (dd, *J* = 12.7, 5.4 Hz, 1H), 2.97 – 2.85 (m, 1H), 2.67 – 2.53 (m, 2H), 2.11 – 2.02 (m, 1H). MS (ESI) $[M + H]^+$ calcd for C19H15N3O5, 365.10; found, 366.12

2-(2,6-dioxopiperidin-3-yl)-4-((2-hydroxyphenyl)amino)isoindoline-1,3-dione (11). 120 mg. Yield 75% ^{1}H NMR (700 MHz, DMSO) δ 11.13 (s, 1H), 9.97 (s, 1H), 8.21 (s, 1H), 7.67 – 7.56 (m, 1H), 7.40 – 7.36 (m, 1H), 7.34 (d, *J* = 8.6 Hz, 1H), 7.21 (d, *J* = 7.0 Hz, 1H), 7.03 – 6.98 (m, 1H), 6.98 – 6.94 (m, 1H), 6.88 – 6.82 (m, 1H), 5.12 (dd, *J* = 12.9, 5.5 Hz, 1H), 2.98 – 2.85 (m, 1H), 2.67 – 2.52 (m, 2H), 2.11 – 2.04 (m, 1H). ^{13}C NMR (151 MHz, DMSO) δ 172.8, 170.0, 168.8, 167.1, 149.4, 143.0, 136.2, 132.2, 126.6, 124.9, 121.9, 119.3, 118.7, 115.8, 112.7, 111.2, 48.7, 31.0, 22.1. HRMS (ESI) $[M + Na]^+$ calcd for C19H15N3O5, 365.1012; found, 388.0905

General procedure for the synthesis of fluorosulfates FL1 to FL4

To a solution of phenol precursors **6, 7, 9** or **10** (1.0 eq) in a mixture of acetonitrile/DMSO (0.1 M, 90:10) to facilitate solubility, triethylamine (2.6 eq.) is added dropwise and stirred 20 min at room temperature. 1-(Fluorosulfuryl)-2,3-dimethyl-1*H*-imidazol-3-ium trifluoromethanesulfonate (2.6 eq.) is added and the reaction stirred at room temperature for 2-3 h until TLC shows no starting phenol. The mixture is then diluted in ethyl acetate and washed with water, aq. 0.5 M HCl and brine. Organic layer is dried over anhydrous Na_2SO_4, filtered and the solvent evaporated under reduced pressure. Desired fluorosulfates were purified by flash chromatography in hexane:ethyl acetate and obtained as yellow powders.

3-((2-(2,6-dioxopiperidin-3-yl)-1,3-dioxoisoindolin-5-yl)amino)phenyl sulfurofluoridate (FL1). 135 mg. Yield 76%. ^{1}H NMR (700 MHz, DMSO) δ 11.10 (s, 1H), 9.53 (s, 1H), 7.77 (d, *J* = 8.2 Hz, 1H), 7.56 (t, *J* = 8.2 Hz, 1H), 7.44 – 7.42 (m, 1H), 7.42 – 7.38 (m, 2H), 7.37 (dd, *J* = 8.1, 2.1 Hz, 1H), 7.22 (dd, *J* = 8.2, 2.4 Hz, 1H), 5.09 (dd, *J* = 12.9, 5.4 Hz, 1H), 2.96 – 2.84 (m, 1H), 2.64 – 2.51 (m, 2H), 2.09 – 2.00 (m, 1H). ^{13}C NMR (151 MHz, DMSO) δ 172.8, 170.0, 167.0, 166.7, 150.3, 148.9, 143.0, 133.8, 131.6, 125.4, 121.1, 119.9, 119.4, 114.2, 111.3, 109.1, 48.8, 30.9, 22.1. ^{19}F NMR (659 MHz, DMSO) δ 38.77. HRMS (ESI) $[M + Na]^+$ calcd for C19H14FN3O7S, 447.0536; found, 470.0403.

4-((2-(2,6-dioxopiperidin-3-yl)-1,3-dioxoisoindolin-5-yl)amino)phenyl sulfurofluoridate (FL2). 116 mg. Yield 65%. ^{1}H NMR (500 MHz, DMSO) δ 11.12 (s, 1H), 9.47 (s, 1H), 7.77 (d, *J* = 8.2 Hz, 1H), 7.66 – 7.51 (m, 2H), 7.47 – 7.31 (m, 4H), 5.10 (dd, *J* = 12.8, 5.4 Hz, 1H), 2.98 – 2.79 (m, 1H), 2.66 – 2.53 (m, 2H), 2.09 – 2.00 (m, 1H). ^{13}C NMR (151 MHz, DMSO) δ 172.8, 170.0, 167.1, 166.8, 149.3, 144.1, 141.4, 133.9, 125.4, 122.4, 120.7, 119.6, 108.8, 48.8, 30.9, 22.1. ^{19}F NMR (471 MHz, DMSO) δ 37.82. HRMS (ESI) $[M + Na]^+$ calcd for C19H14FN3O7S, 447.0536; found, 470.0420.

3-((2-(2,6-dioxopiperidin-3-yl)-1,3-dioxoisoindolin-4-yl)amino)phenyl sulfurofluoridate (FL3). 135 mg. Yield 79%. ^{1}H NMR (600 MHz, DMSO) δ 11.14 (s, 1H), 8.86 (s, 1H), 7.74 – 7.65 (m, 1H), 7.63 – 7.57 (m, 1H), 7.57 – 7.50 (m, 2H),

7.49 – 7.42 (m, 1H), 7.39 (d, *J* = 7.0 Hz, 1H), 7.28 – 7.19 (m, 1H), 5.14 (dd, *J* = 12.8, 5.5 Hz, 1H), 2.98 – 2.86 (m, 1H), 2.66 – 2.52 (m, 2H), 2.12 – 2.04 (m, 1H). ^{13}C NMR (151 MHz, DMSO) δ 172.8, 169.9, 167.6, 166.9, 150.2, 142.6, 141.0, 136.2, 132.7, 131.3, 121.2, 120.4, 115.0, 114.7, 114.1, 112.5, 48.8, 30.9, 22.1. ^{19}F NMR (471 MHz, DMSO) δ 38.86. HRMS (ESI) $[M + H]^+$ calcd for C19H14FN3O7S, 447.0536; found, 448.0611.

4-((2-(2,6-dioxopiperidin-3-yl)-1,3-dioxoisoindolin-4-yl)amino)phenyl sulfurofluoridate (FL4). 107 mg. Yield 60%. ^{1}H NMR (600 MHz, DMSO) δ 11.13 (s, 1H), 8.72 (s, 1H), 7.69 – 7.62 (m, 1H), 7.59 – 7.44 (m, 5H), 7.36 – 7.33 (m, 1H), 5.13 (dd, *J* = 12.8, 5.5 Hz, 1H), 2.97 – 2.85 (m, 1H), 2.67 – 2.54 (m, 2H), 2.08 – 2.03 (m, 1H). ^{13}C NMR (151 MHz, DMSO) δ 172.8, 170.0, 167.8, 166.9, 144.8, 141.6, 140.8, 136.2, 132.6, 122.3, 122.1, 120.6, 114.6, 113.5, 48.7, 30.9, 22.1. ^{19}F NMR (471 MHz, DMSO) δ 37.90. HRMS (ESI) $[M + Na]^+$ calcd for C19H14FN3O7S, 447.0536; found, 470.0429.

General procedure for the synthesis of fluorosulfates FL5, FL6 and FL7

FL5, **FL6** and **FL7** are synthesized from the corresponding phenol precursors (150 mg, 0.7 mmol) as **FL1** to **FL4** using acetonitrile as solvent and triethylamine (155 µL, 0.91 mmol, 1.3eq.) and 1-(fluorosulfuryl)-2,3-dimethyl-1*H*-imidazol-3-ium trifluoromethanesulfonate (298 mg, 0.91 mmol, 1.3 eq.). Phenol precursors were provided by WuXi. Final compounds are purified by flash chromatography using hexane:ethyl acetate as solvents and obtained as white powders.

4-(2,6-dioxopiperidin-3-yl)phenyl sulfurofluoridate (FL5). 176 mg. Yield 88%. ^{1}H NMR (700 MHz, DMSO) δ 10.90 (s, 1H), 7.59 – 7.53 (m, 2H), 7.49 – 7.42 (m, 2H), 4.00 (dd, *J* = 12.5, 4.9 Hz, 1H), 2.74 – 2.66 (m, 1H), 2.57 – 2.52 (m, 1H), 2.30 – 2.22 (m, 1H), 2.08 – 2.01 (m, 1H). ^{13}C NMR (176 MHz, $CDCl_3$) δ 173.9, 173.3, 148.5, 140.5, 131.1, 120.9, 46.8, 31.6, 25.7. ^{19}F NMR (659 MHz, DMSO) δ 38.41. HRMS (ESI) $[M + Na]^+$ calcd for C11H10FNO5S, 287.0264; found, 310.0155.

3-(2,6-dioxopiperidin-3-yl)phenyl sulfurofluoridate (FL6). 184 mg. Yield 92%. ^{1}H NMR (700 MHz, DMSO) δ 10.90 (s, 1H), 7.56 (t, *J* = 7.9 Hz, 1H), 7.53 – 7.49 (m, 2H), 7.43 – 7.39 (m, 1H), 4.02 (dd, *J* = 12.5, 4.9 Hz, 1H), 2.74 – 2.65 (m, 1H), 2.57 – 2.51 (m, 1H), 2.33 – 2.24 (m, 1H), 2.07 – 1.99 (m, 1H). ^{13}C NMR (176 MHz, DMSO) δ 173.6, 173.2, 149.6, 142.5, 130.6, 129.7, 121.3, 119.5, 47.0, 31.6, 25.5. ^{19}F NMR (659 MHz, DMSO) δ 38.60. HRMS (ESI) $[M + H]^+$ calcd for C11H10FNO5S, 287.0264; found, 288.0339.

2-(2,6-dioxopiperidin-3-yl)phenyl sulfurofluoridate (FL7). 160 mg. Yield 80%. ^{1}H NMR (700 MHz, DMSO) δ 10.95 (s, 1H), 7.60 – 7.56 (m, 1H), 7.54 – 7.47 (m, 3H), 4.24 (dd, *J* = 12.9, 4.9 Hz, 1H), 2.87 – 2.79 (m, 1H), 2.59 – 2.54 (m, 1H), 2.33 – 2.24 (m, 1H), 2.03 – 1.95 (m, 1H). ^{13}C NMR (176 MHz, DMSO) δ 173.3, 173.0, 148.6, 131.3, 131.2, 129.6, 129.1, 120.7, 41.9, 31.7, 24.8. ^{19}F NMR (659 MHz, DMSO) δ 42.03. HRMS (ESI) $[M + H]^+$ calcd for C11H10FNO5S, 287.0264; found, 288.0329.

Synthesis of cyclic sulfamates 12, 13

12 and **13** are synthesized as reported for **FL1**-**FL4**.

5-(2,2-dioxido-3H-benzo[d][1,2,3]oxathiazol-3-yl)-2-(2,6-dioxopiperidin-3-yl)isoindoline-1,3-dione (12). 93 mg. Yield 80%. ^{1}H NMR (700 MHz, $CDCl_3$) δ 8.21 (s, 1H), 8.06 (d, *J* = 8.0 Hz, 1H), 8.04 – 8.00 (m, 1H), 7.93 (dd, *J* = 8.0, 2.0 Hz, 1H), 7.28 – 7.23 (m, 1H), 7.23 – 7.14 (m, 2H), 6.91 (dd, *J* = 7.7, 1.5 Hz, 1H), 5.02 (dd, *J* = 12.8, 5.4 Hz, 1H), 2.96 – 2.91 (m, 1H), 2.89 – 2.82 (m, 1H), 2.81 – 2.74 (m, 1H), 2.22 – 2.14 (m, 1H). HRMS (ESI) $[M + Na]^+$ calcd for C19H13N3O7S, 427.0474; found, 450.0363

4-(2,2-dioxido-3H-benzo[d][1,2,3]oxathiazol-3-yl)-2-(2,6-dioxopiperidin-3-yl)isoindoline-1,3-dione (13). 88 mg. Yield 75%. ^{1}H NMR (500 MHz, $CDCl_3$) δ 8.10 (s, 1H), 8.06 – 8.01 (m, 1H), 7.93 (td, *J* = 7.7, 2.3 Hz, 1H), 7.85 (t, *J* = 1.0

Hz, 1H), 7.26 – 7.22 (m, 1H), 7.16 – 7.10 (m, 2H), 6.72 – 6.66 (m, 1H), 4.96 (dd, *J* = 12.5, 5.4 Hz, 1H), 2.92 – 2.87 (m, 1H), 2.81 – 2.76 (m, 1H), 2.73 – 2.67 (m, 1H), 2.17 – 2.10 (m, 1H).^{13}C NMR (151 MHz, $CDCl_3$) δ 170.5, 167.4, 165.8, 163.9, 142.1, 136.6, 134.4, 134.0, 131.4, 130.6, 128.0, 125.0, 125.0, 124.1, 112.0, 111.8, 49.6, 31.2, 22.4. HRMS (ESI) $[M + Na]^+$ calcd for C19H13N3O7S, 427.0474; found, 450.0365

High Throughput Synthesis of sulfamate esters

Reactions (0.2 M) were performed in 384-well plates using fluorosulfates FL1 – FL6 (1.0 eq.) and a library of 21 secondary amines (1.0 eq.) with HOBt (0.3 eq.) as catalyst, TBDMS (2.0 eq.) as additive, and DIPEA (1.0, 2.0 or 3.0 eq. for neutral amines, HCl salts amines or 2xHCl amines, respectively). Stock solutions of fluorosulfates (0.51 M), amines (0.83 M) and HOBt (0.72 M) were prepared in DMSO. To a solution of 7.8 µL of fluorosulfate, 4.8 µL amine, 1.56 µL TBDMS, 2 µL HOBt and DIPEA (0.77 µL for neutral amines, 1.54 µL for HCl salts and 2.31 µL for 2xHCl salts) were added in a total volume of 20 µL. The plate was sealed and shaken at 65 C for 48 hours. Conversion was monitored via TLC showing high to full conversion in all wells.

Table S 1: Library of secondary amines used for the high throughput synthesis

Amine	Structure	MW	Amine	Structure	MW
**1**<br>EN300-209069	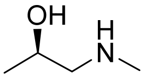	89.1	**11**<br>EN300-7444635	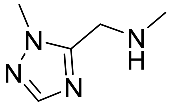	162.6<br>(HCl)
**2**<br>EN300-7413100	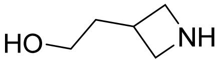	137.6<br>(HCl)	**12**<br>EN300-7436813	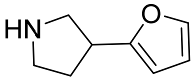	173.6<br>(HCl)
**3**<br>EN300-<br>26619723	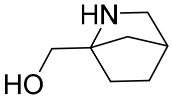	163.3<br>(HCl)	**13**<br>EN300-6748344	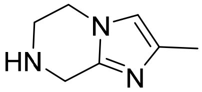	210.1<br>(2x HCl)
**4**<br>EN300-6759138	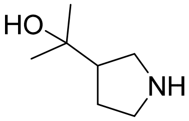	165.7<br>(HCl)	**14**<br>EN300-<br>26621514	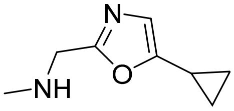	225.1<br>(2x HCl)
5<br>Aldrich	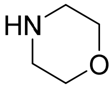	87.1	**15**<br>EN300-189562	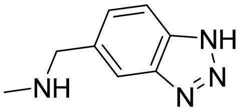	162.2
**6**<br>EN300-7608240	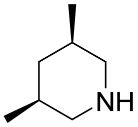	149.7<br>(HCl)	**16**<br>EN300-180608	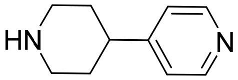	235.2<br>(2x HCl)
**7**<br>EN300-7542742	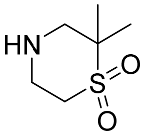	199.7<br>(HCl)	**17**<br>EN300-75743	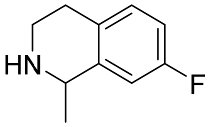	165.2
**8**<br>EN300-7564409	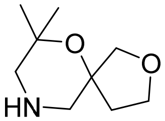	207.7<br>(HCl)	**18**<br>EN300-<br>26623744	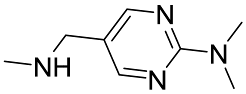	239.1<br>(2x HCl)

**9**<br>EN300-1605226	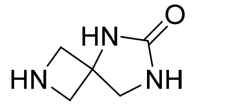	163.3<br>(HCl)	**19**<br>EN300-06743	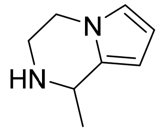	136.2
**10**<br>EN300-7355378	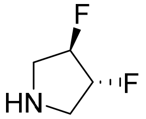	143.6<br>(HC)	**20**<br>EN300-12816	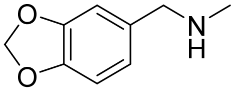	165.2
			**21 (ALDRICH)**<br>CAS# 67376-94-7	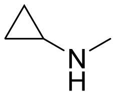	107.6<br>(HCl)

Synthesis of FL2-14

A mixture of fluorosulfate **FL2** (10 mg, 0.022 mmol), 14 (4.9 mg, 0.022 mmol), TBDMS (8 µL, 0.044 mmol), HOBt (1.0 mg, 0.007 mmol) and DIPEA (12µL, 0.066 mmol) in 0.17 mL ethyl acetate:DMSO 1:1 is stirred at 60 C for 16 hours. The reaction is then diluted with ethyl acetate and washed with water and aq. 0.5 M HCl. Organic layer is dried over anh. Na_2SO_4, filtered and the solvent evaporated under reduced pressure. Final compound is purified via flash chromatography using hexane:ethylacetate as solvents.

4-((2-(2,6-dioxopiperidin-3-yl)-1,3-dioxoisoindolin-5-yl)amino)phenyl ((5-cyclopropyloxazol-2-yl)methyl)(methyl)sulfamate (FL2-14). 7 mg. 55% yield. ^{1}H NMR (700 MHz, MeOD) δ 7.68 (d, *J* = 8.3 Hz, 1H), 7.41 (d, *J* = 2.2 Hz, 1H), 7.29 (dd, *J* = 8.3, 2.2 Hz, 1H), 7.28 – 7.21 (m, 4H), 6.78 (d, *J* = 0.8 Hz, 1H), 5.08 (dd, *J* = 12.7, 5.6 Hz, 1H), 4.50 (s, 2H), 3.05 (s, 3H), 2.92 – 2.80 (m, 1H), 2.80 – 2.64 (m, 2H), 2.16 – 2.06 (m, 1H), 2.00 – 1.90 (m, 1H), 1.00 – 0.93 (m, 2H), 0.80 – 0.72 (m, 2H). ^{13}C NMR (176 MHz, $CDCl_3$) δ 174.6, 171.6, 169.1, 168.8, 158.8, 157.6, 152.0, 146.7, 141.1, 135.8, 126.3, 124.2, 122.6, 122.0, 121.9, 120.1, 109.6, 50.5, 48.5, 37.0, 32.2, 23.8, 7.2, 7.1. HRMS (ESI) $[M + H]^+$ calcd for C27H25N5O8S 579.1424, found 580.1491.

Synthesis of fluorescent tracer 16

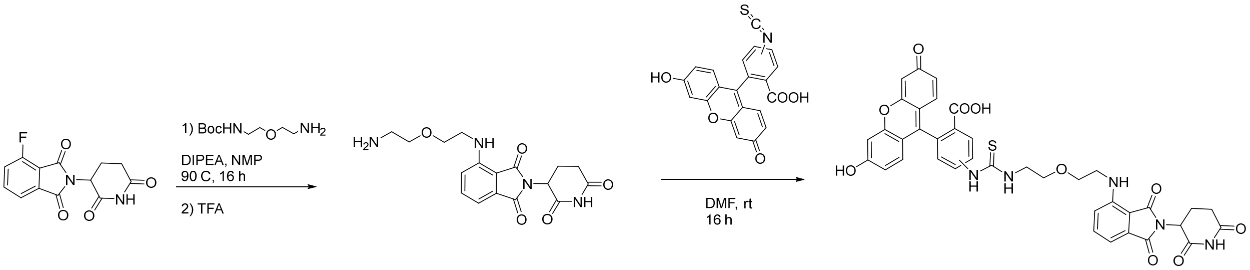

14 15 16

4-Fluoro-2-(2,6-dioxopiperidin-3-yl)isoindoline-1,3-dione (14). A mixture of 3-fluorophthalic anhydride (200 mg, 0.88 mmol), 3-aminopiperidine-2, 6-dione hydrochloride (197 mg, 0.88 mmol) and sodium acetate (118 mg, 2.4 mmol) in acetic acid (4.4 mL) is stirred at 120 C for 16 h. Once the reaction is completed, the mixture is cooled, water is added, and the desired product collected by filtration. Characterization data correspond to those previously reported.[4] 194 mg. Yield 80% ^{1}H NMR (600 MHz, DMSO) δ 11.14 (s, 1H), 7.98 – 7.92 (m, 1H), 7.79 (d, *J* = 7.3 Hz, 1H), 7.74 (t, *J* = 8.8 Hz, 1H), 5.16 (dd, *J* = 13.0, 5.4 Hz, 1H), 2.94 – 2.84 (m, 1H), 2.65 – 2.58 (m, 1H), 2.56 – 2.51 (m, 1H), 2.11 – 2.03 (m, 1H). (ESI) $[M + Na]^+$ calcd for C13H9FN2O4 276.05, found 299.11.

4-((2-(2-aminoethoxy)ethyl)amino)-2-(2,6-dioxopiperidin-3-yl)isoindoline-1,3-dione (15). A mixture of of 14 (50 mg, 0.18 mmol), tert-butyl (2-(2-aminoethoxy)ethyl)carbamate (51 mg, 0.25 mg) and DIPEA (99 uL, 0.56 mmol) in N-methyl-2-pyrrolidone (1.0 mL) is stirred at 90 C for 16 h until no starting material is observed. The mixture is diluted in ethyl acetate and washed with water. Organic layer is dried over anh. Na_2SO_4, the solvent evaporated under reduced pressure and the desired intermediate purified by flash chromatography using hexane:ethyl acetate as solvents. The resulting Boc-protected compound (45 mg) is dissolved in dichloromethane and TFA (0.25 mL) is added dropwise at 0 C. The reaction is warmed to room temperature and after 1 h no starting material is observed. After solvent evaporation, the compound is used in the next step without further purification.

5-(3-(2-(2-((2-(2,6-dioxopiperidin-3-yl)-1,3-dioxoisoindolin-4-yl)amino)ethoxy)ethyl)thioureido)-2-(6-hydroxy-3-oxo-3H-xanthen-9-yl)benzoic acid (16). To a solution of 15-TFA salt (57 mg, 0.125 mmol) in anhydrous DMF (2.5 mL), TEA (61 uL, 0.44 mmol) is added under Ar and stirred for 5 min. FITC (48 mg, 0.125 mmol) is added and the reaction stirred at room temperature protected from light during 16 h. Solvent is evaporated and the final compound purified by flash chromatography using ethyl acetate:methanol as solvents. 82 mg, 88% yield. ^{1}H NMR (600 MHz, DMSO) δ 11.09 (s, 1H), 10.13 (s, 2H), 10.03 (s, 1H), 8.26 (s, 1H), 8.15 (s, 1H), 7.73 (d, *J* = 8.3 Hz, 1H), 7.58 (dd, *J* = 8.6, 7.1 Hz, 1H), 7.17 (dd, *J* = 8.4, 2.0 Hz, 2H), 7.03 (d, *J* = 7.0 Hz, 1H), 6.70 – 6.64 (m, 3H), 6.63 – 6.59 (m, 2H), 6.59 – 6.54 (m, 2H), 5.05 (dd, *J* = 12.8, 5.4 Hz, 1H), 3.78 – 3.63 (m, 6H), 3.56 – 3.50 (m, 2H), 2.88 – 2.82 (m, 1H), 2.60 – 2.54 (m, 1H), 2.54 – 2.51 (m, 1H), 2.04 – 1.99 (m, 1H). ^{13}C NMR (151 MHz, DMSO) δ 180.6, 172.8, 170.1, 169.0, 168.5, 167.3, 159.5, 151.9, 147.2, 146.4, 141.3, 136.2, 132.1, 129.4, 129.0, 126.6, 124.0, 117.4, 116.4, 112.6, 110.7, 109.7, 109.3, 102.2, 83.0, 68.8, 68.3, 48.5, 43.6, 41.7, 31.0, 22.1. HRMS (ESI) $[M + H]^+$ calcd for C38H31N5O10S 749.1792, found 750.1868.

^{1}H, ^{13}C and ^{19}F NMR spectra

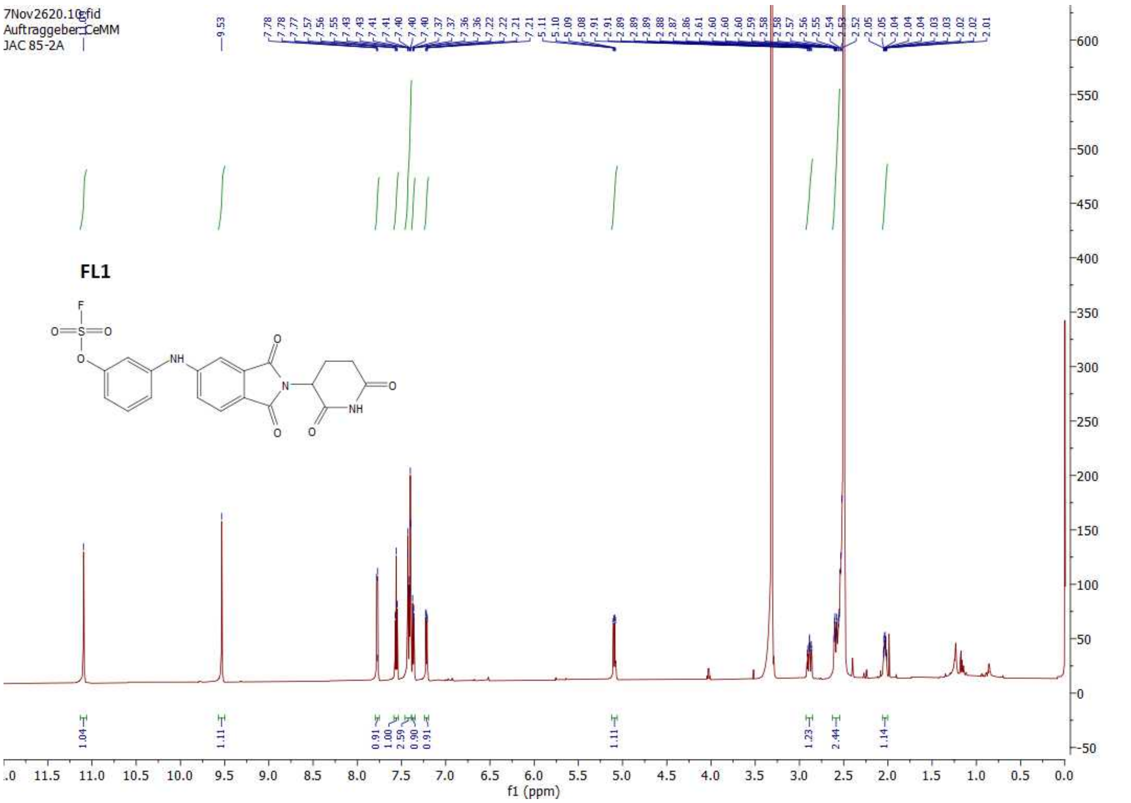

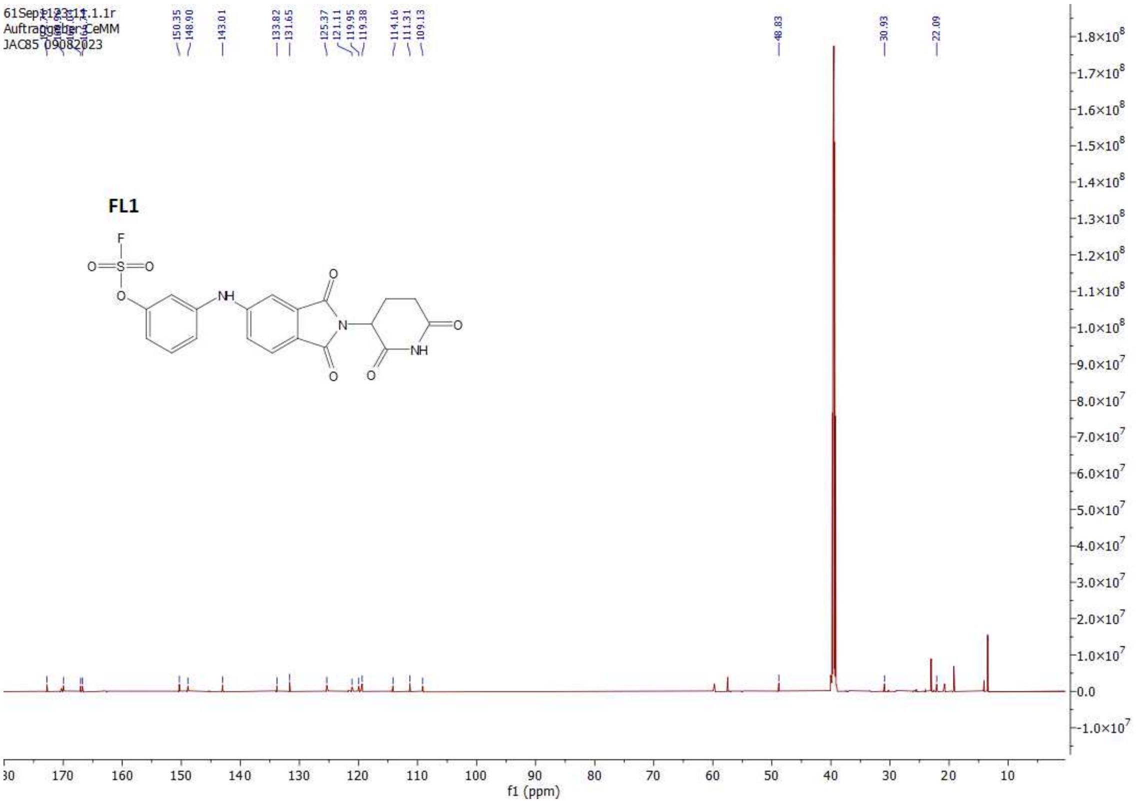

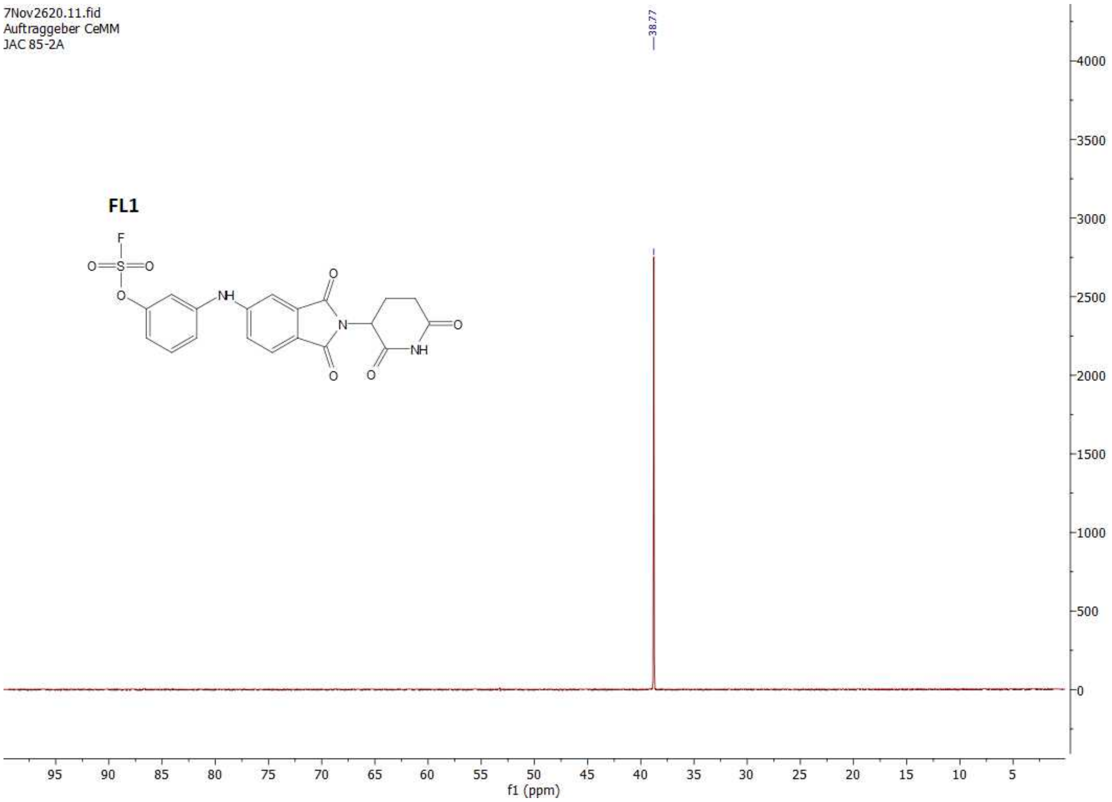
7Nov2620.11.fid
Auftraggeber CeMM
JAC 85-2A
38.77
FL1
f1 (ppm)

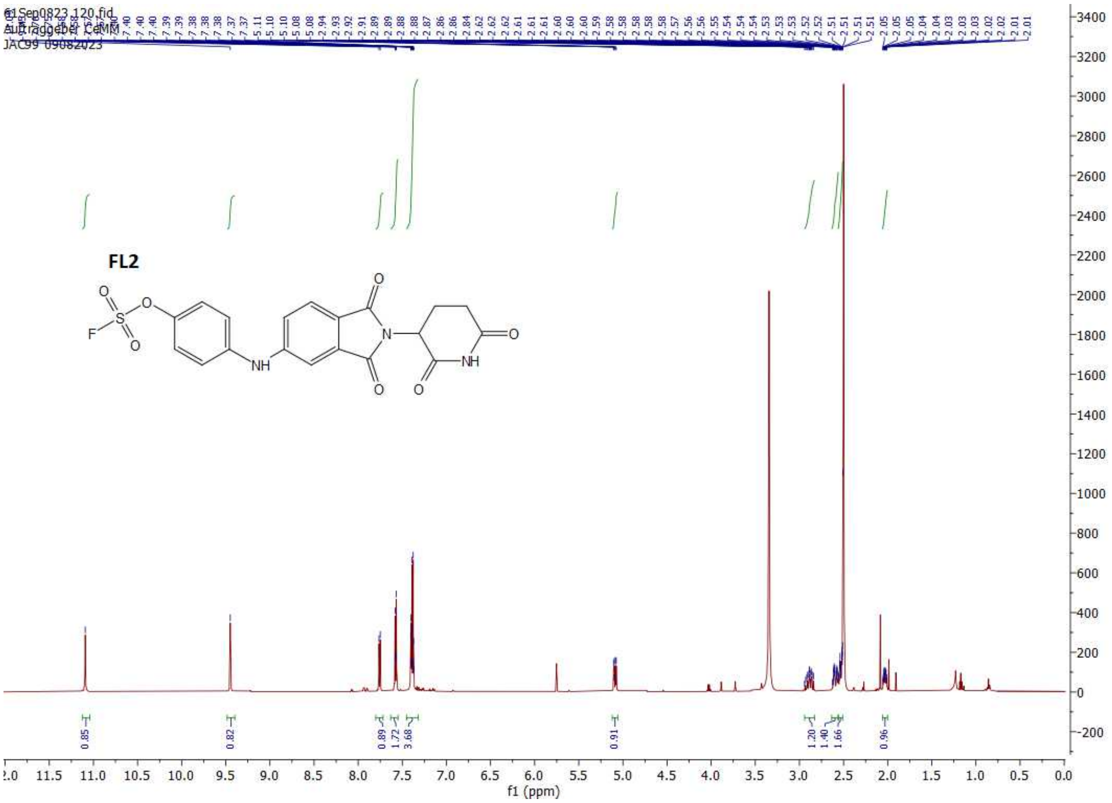
Auftraggeber CeMM
JAC99 09082023
FL2
f1 (ppm)

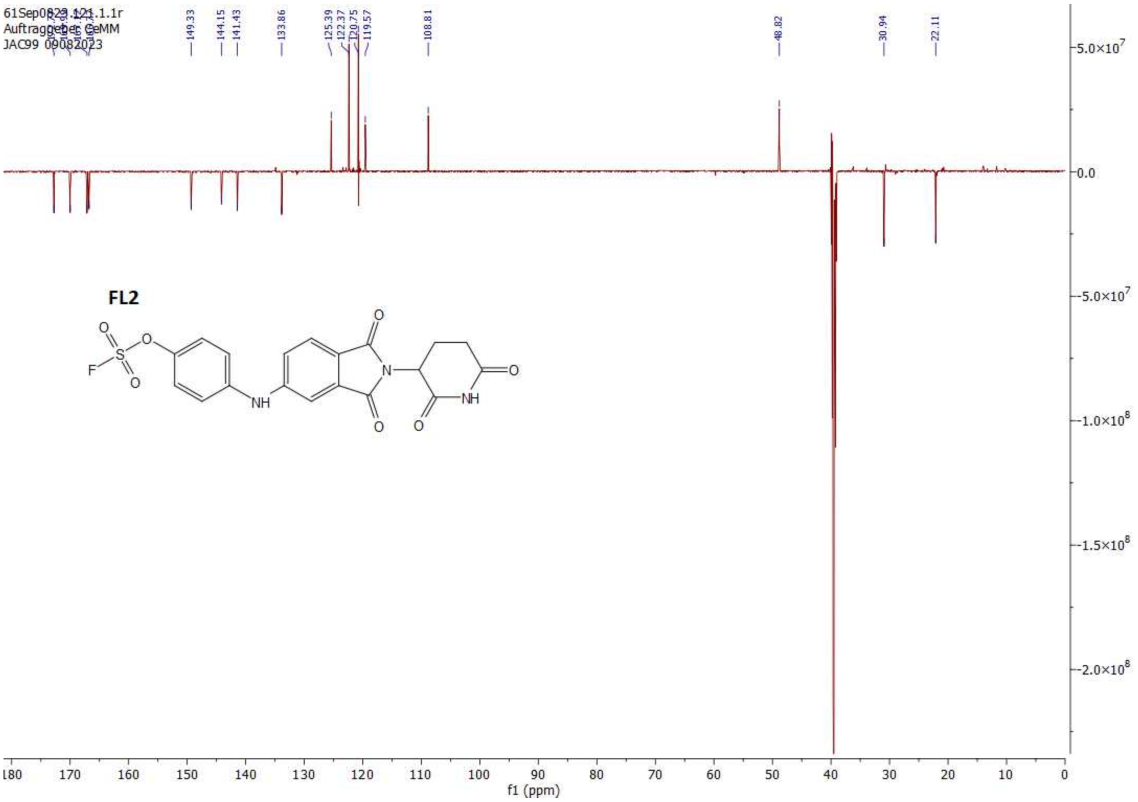

61Sep0829.121.1.1r
Auftraggeber GeMM
JAC99 09082023
149.33
144.15
141.43
133.86
125.39
122.37
120.75
119.57
108.81
48.82
30.94
22.11
FL2
f1 (ppm)

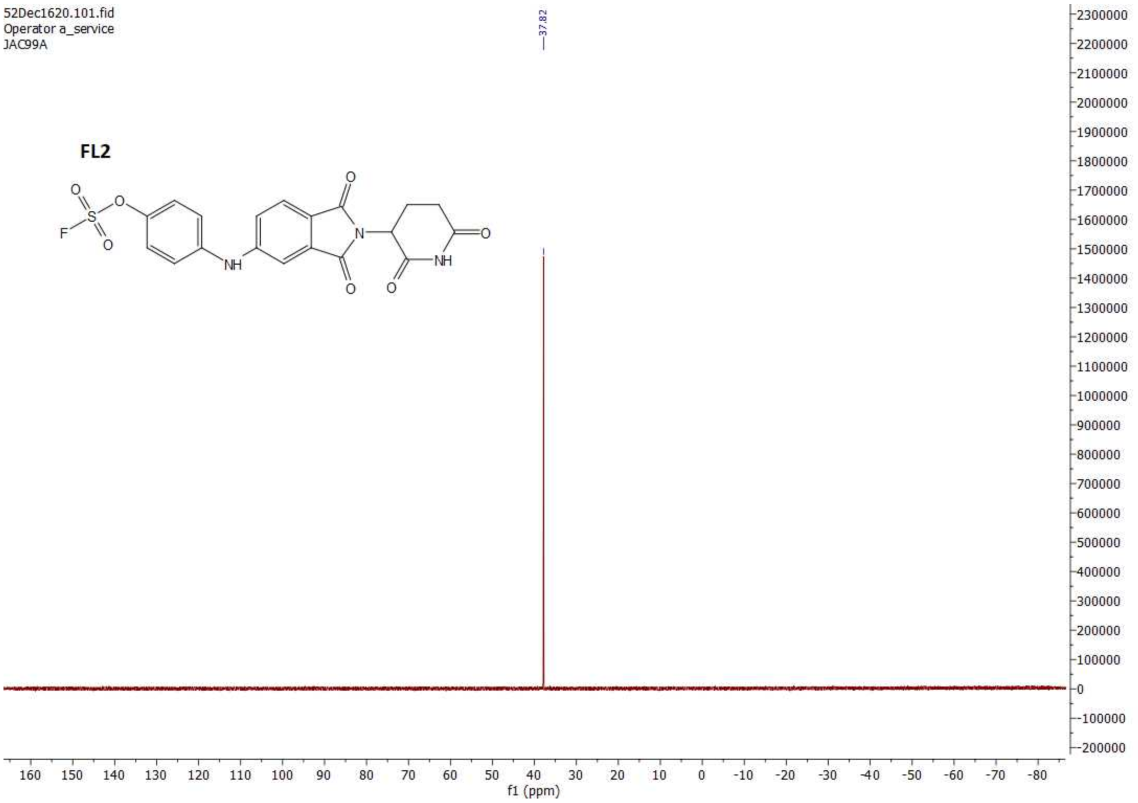

52Dec1620.101.fid
Operator a_service
JAC99A
37.82
FL2
f1 (ppm)

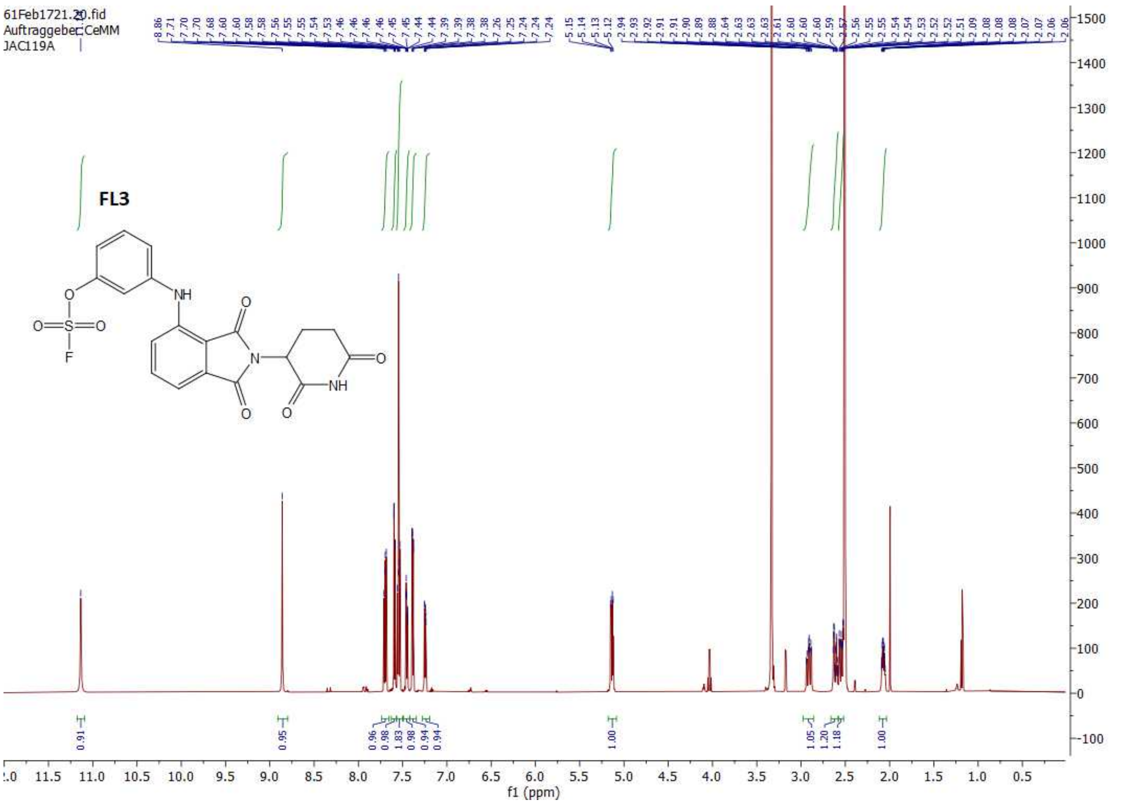
61Feb1721.20.fid
Auftraggeber CeMM
JAC119A
FL3
f1 (ppm)

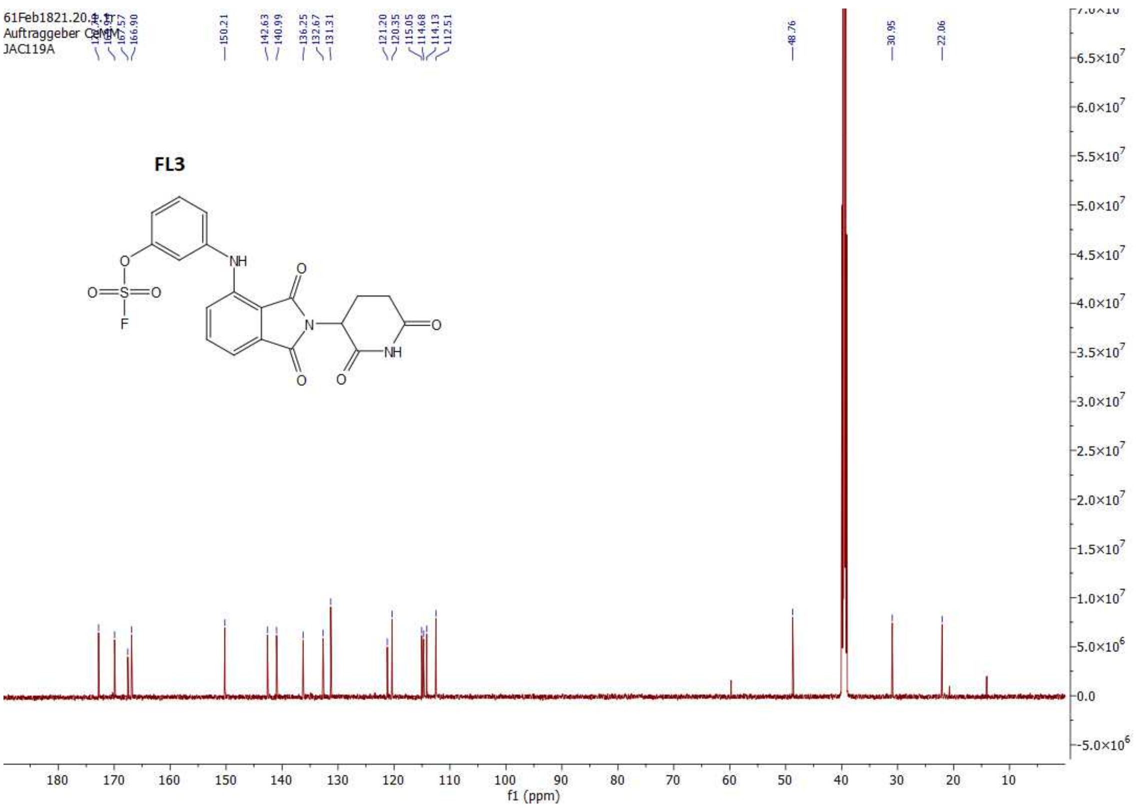
Auftraggeber CeMM
JAC119A
FL3
f1 (ppm)

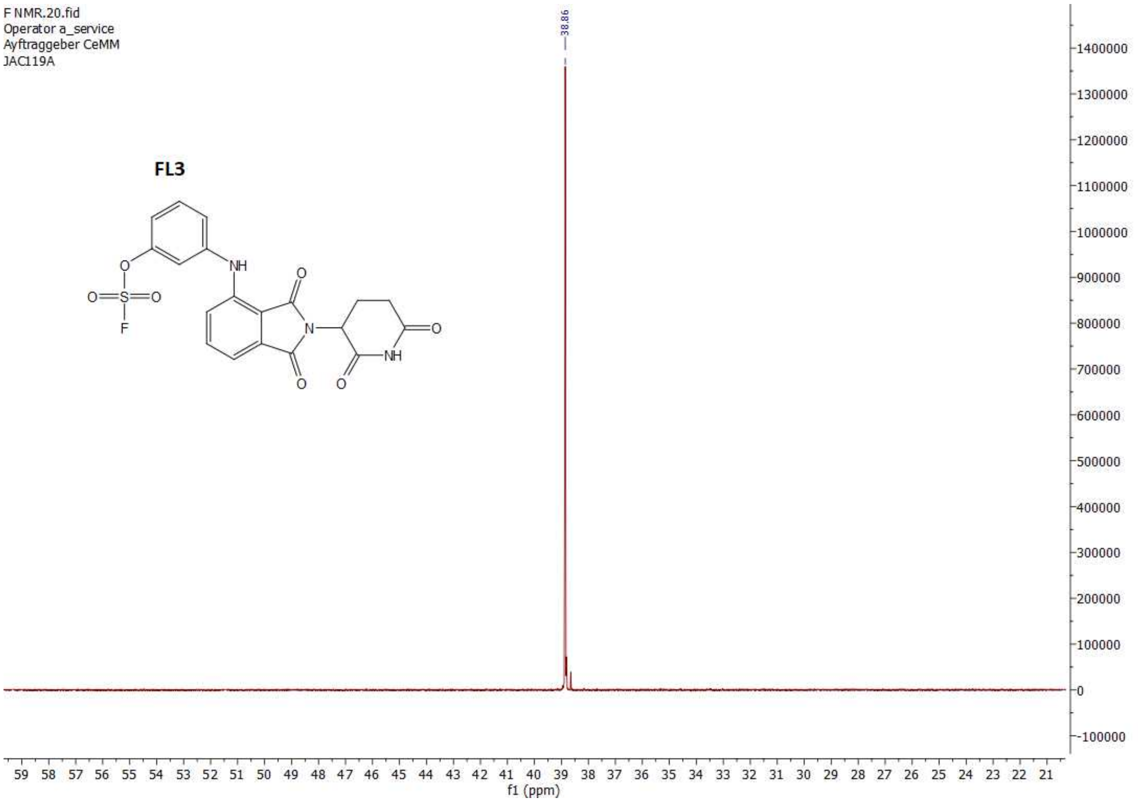
F NMR.20.fid
Operator a_service
Ayftraggeber CeMM
JAC119A
FL3
38.86
f1 (ppm)

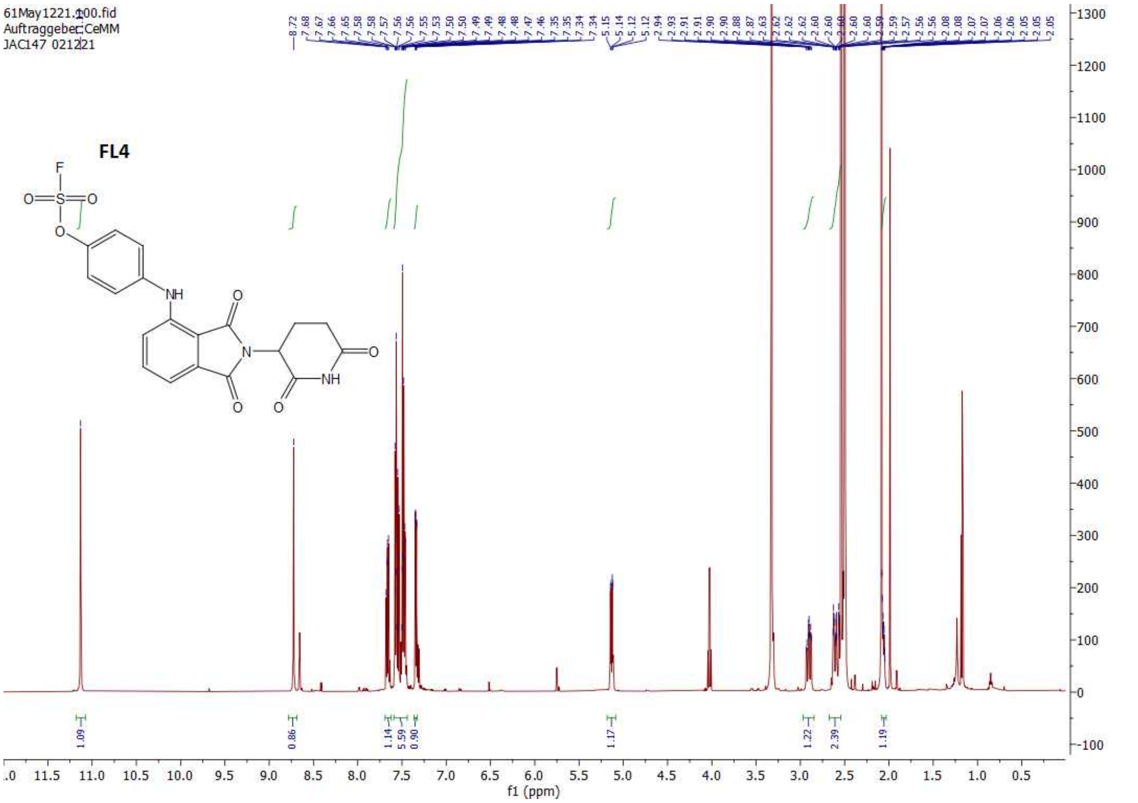
Auftraggeber CeMM
JAC147 021221
FL4
f1 (ppm)

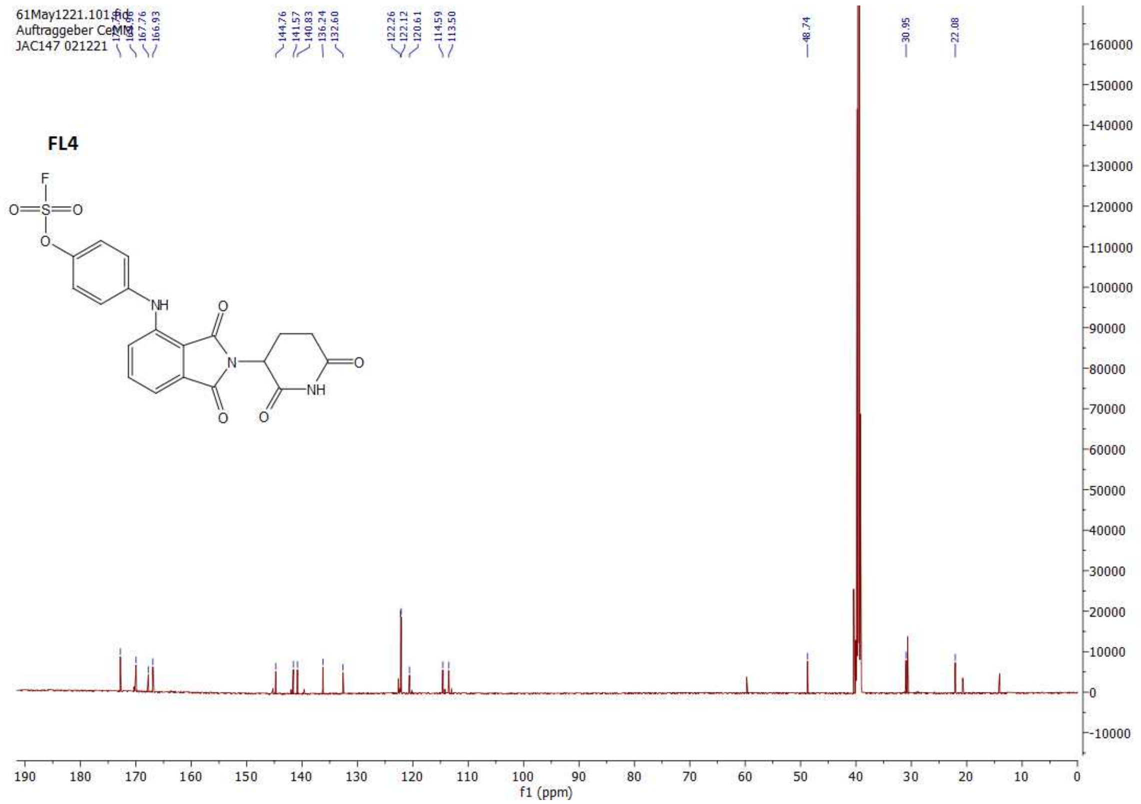
61May1221.101.fid
Auftraggeber CeMM
JAC147 021221
FL4
144.76
141.57
140.83
136.24
132.60
122.26
122.12
120.61
114.59
113.50
48.74
30.95
22.08
f1 (ppm)

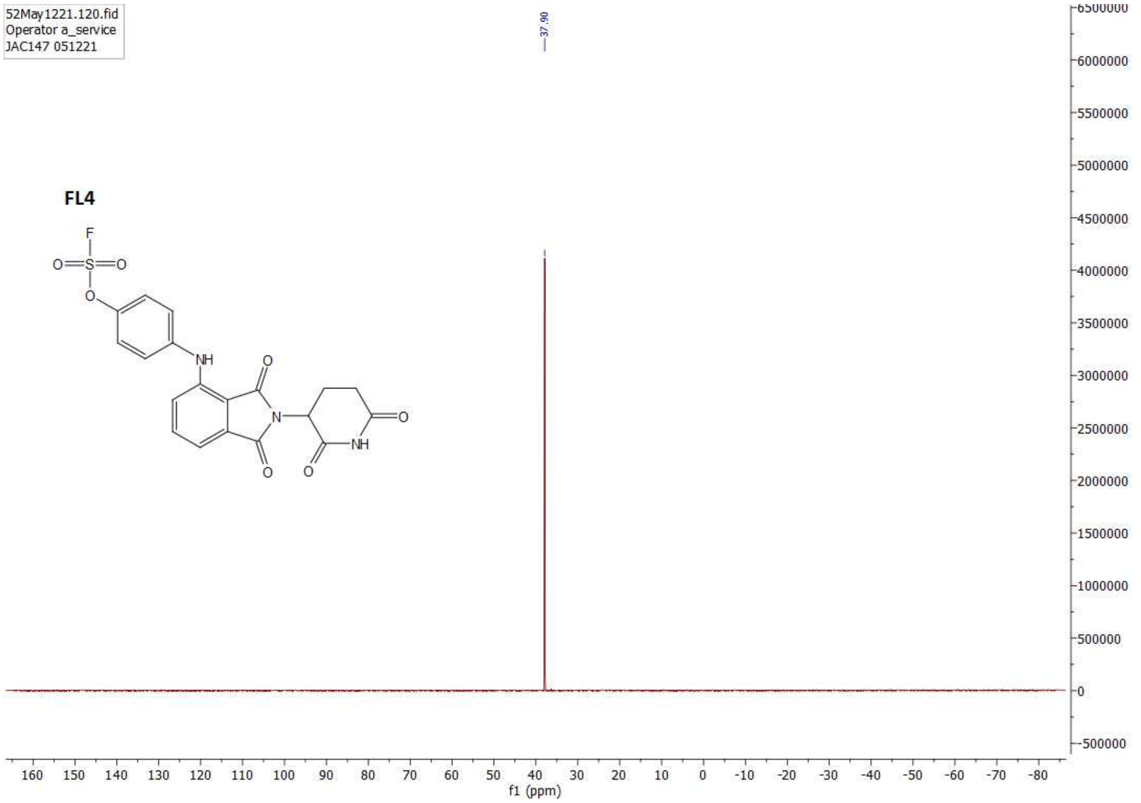
52May1221.120.fid
Operator a_service
JAC147 051221
FL4
37.90
f1 (ppm)

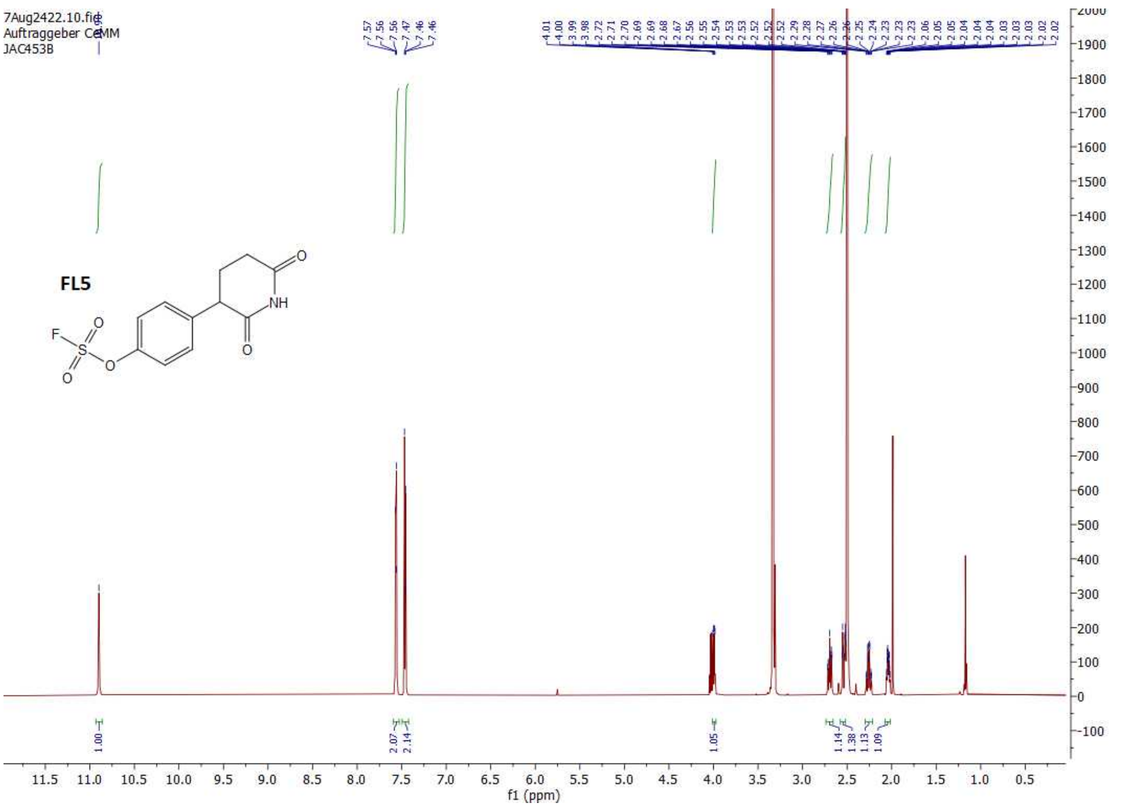
7Aug2422.10.fid
Auftraggeber CeMM
JAC453B
FL5
1.00
2.07
2.14
1.05
1.14
1.38
1.13
1.09
f1 (ppm)

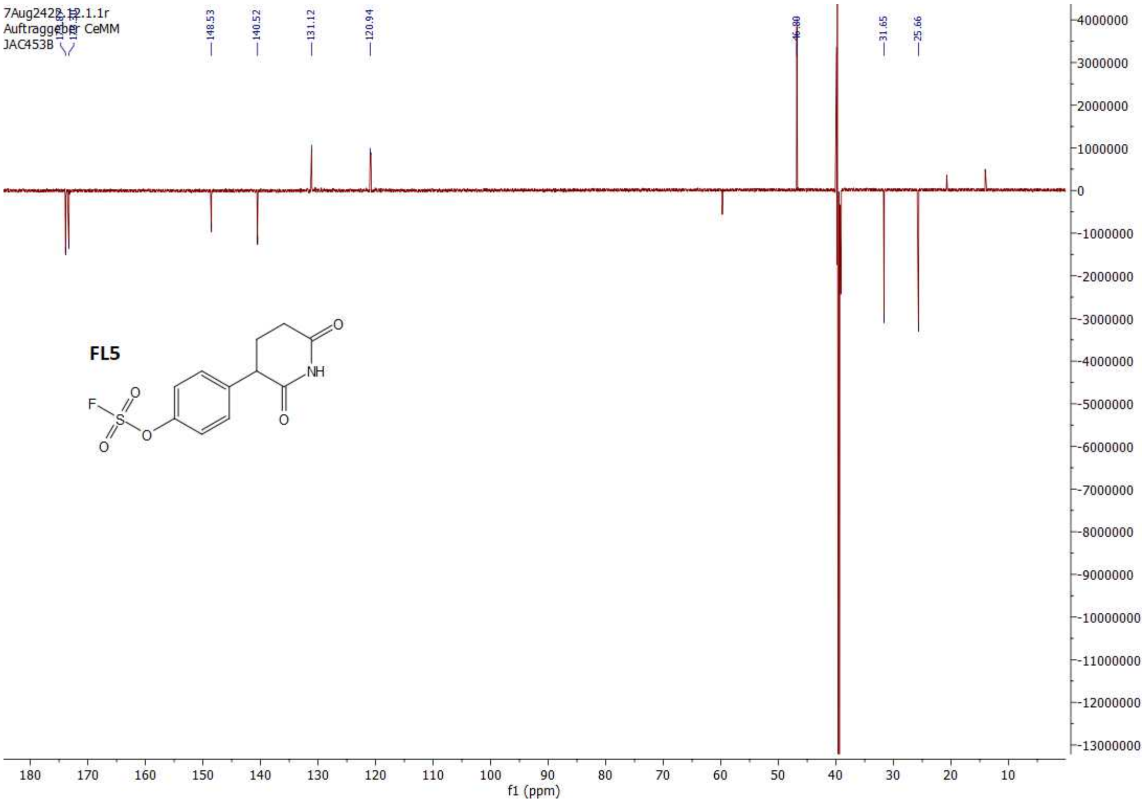
Auftraggeber CeMM
JAC453B
148.53
140.52
131.12
120.94
31.65
25.66
FL5
f1 (ppm)

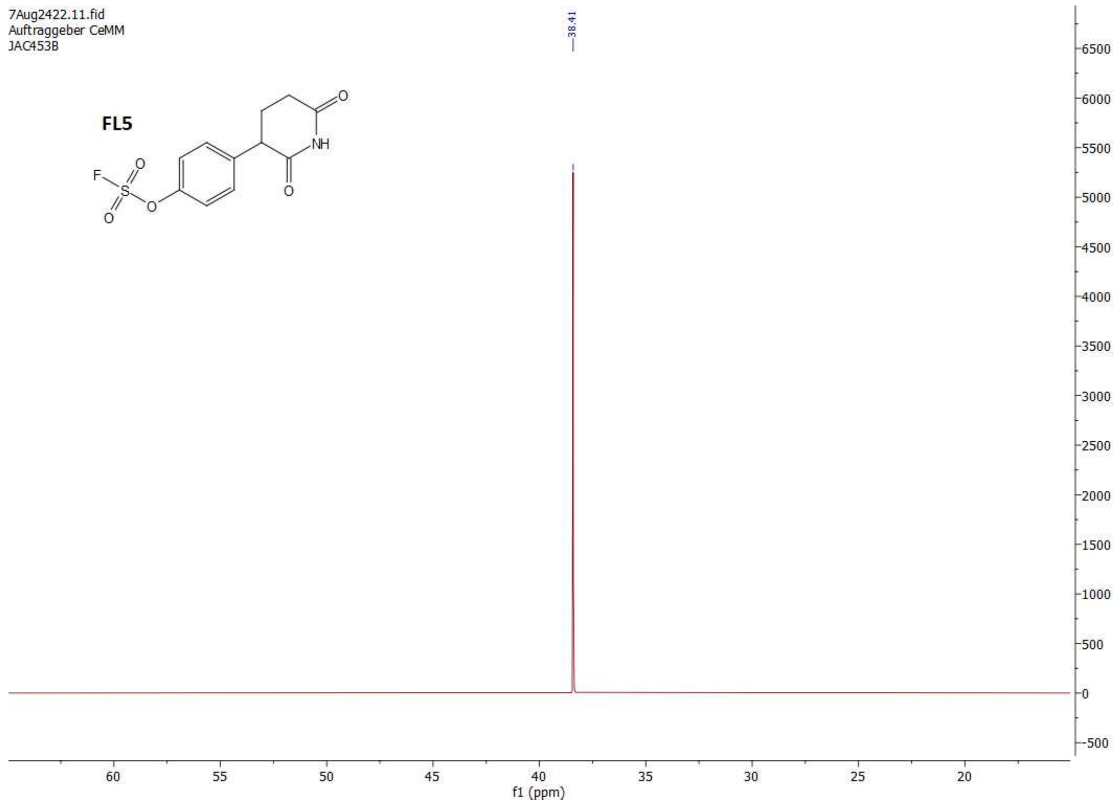
7Aug2422.11.fid
Auftraggeber CeMM
JAC453B
FL5
38.41
f1 (ppm)

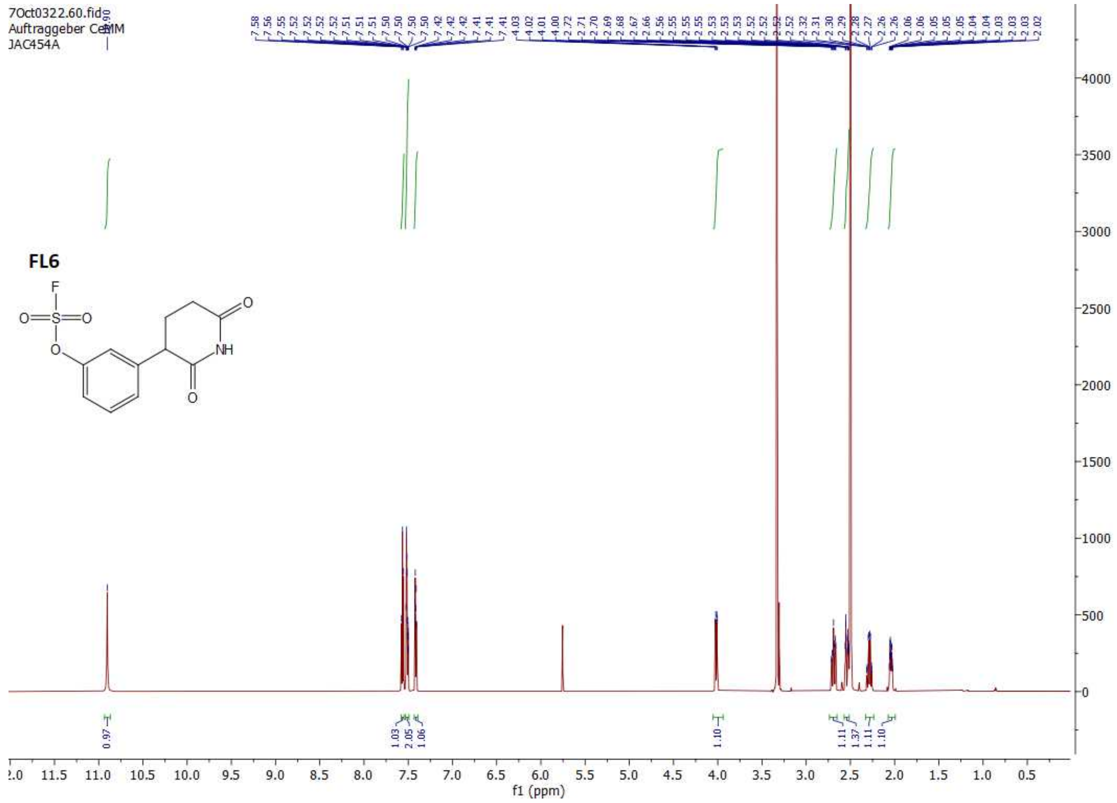
7Oct0322.60.fid
Auftraggeber CeMM
JAC454A
FL6
0.97
1.03
2.05
1.06
1.10
1.11
1.37
1.11
1.10
f1 (ppm)

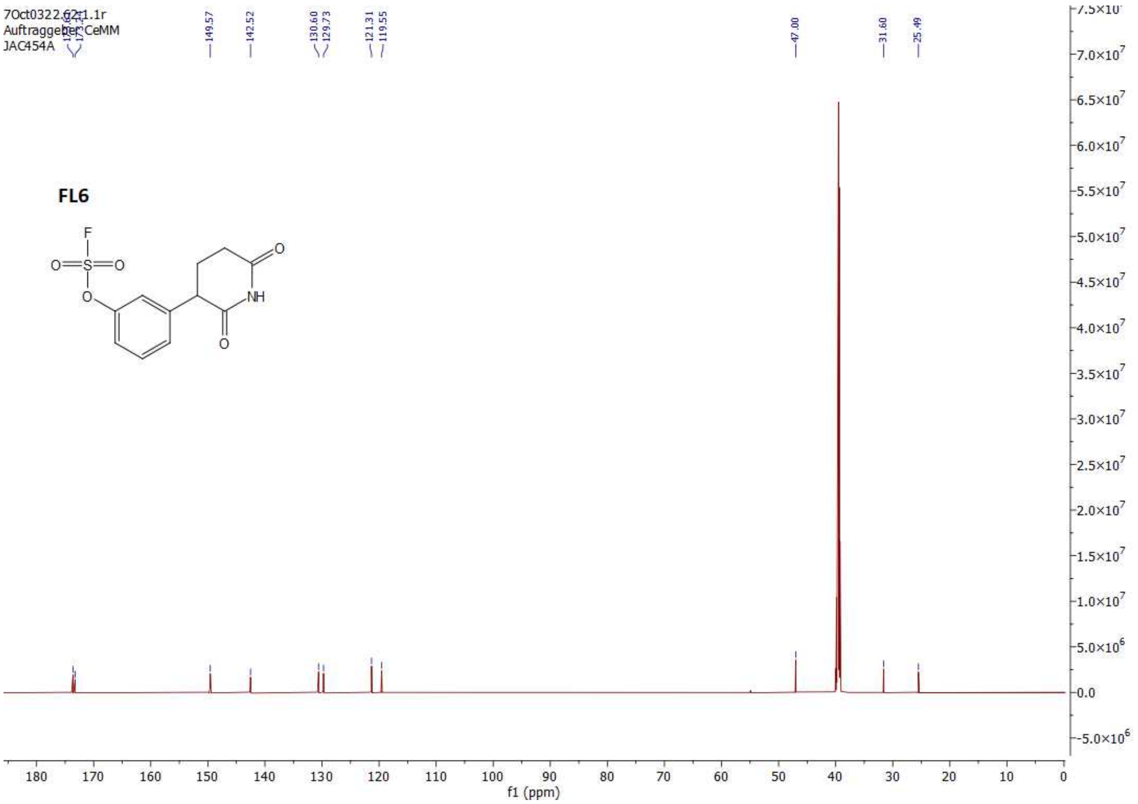
Auftraggeber CeMM
JAC454A
149.57
142.52
130.60
129.73
121.31
119.55
47.00
31.60
25.49
FL6
F
O
S
O
O
NH
O
O
f1 (ppm)

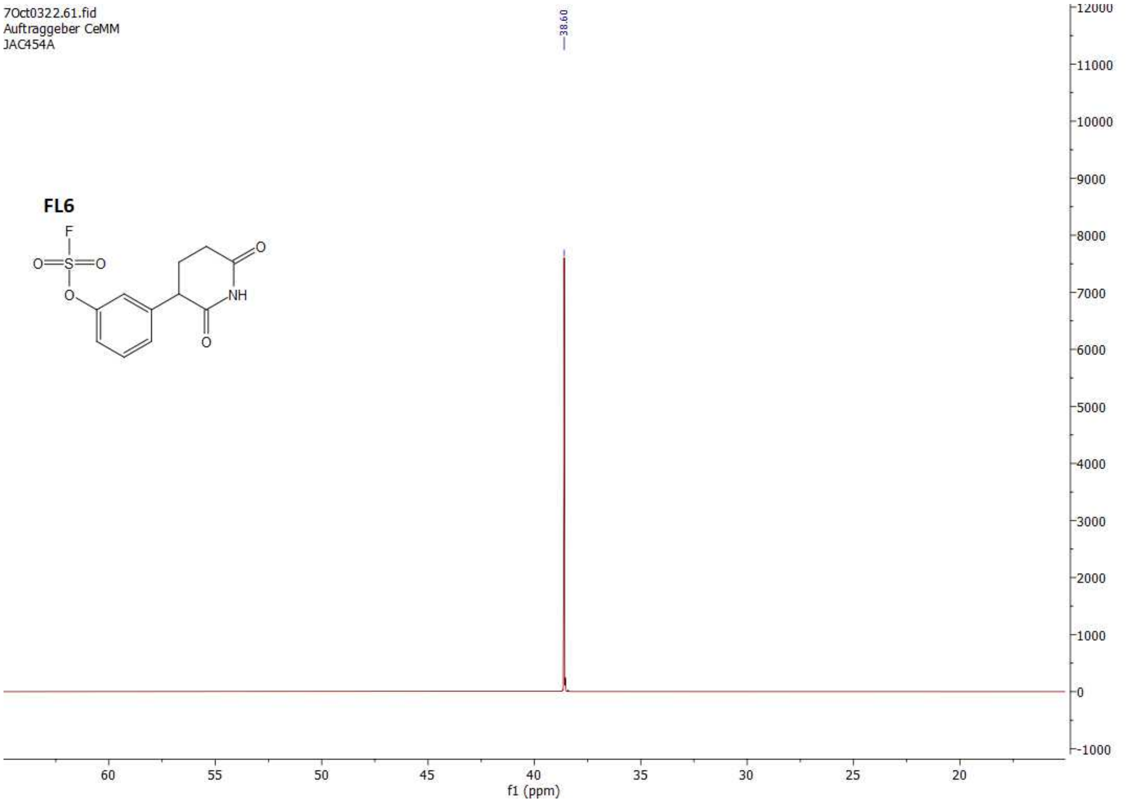
7Oct0322.61.fid
Auftraggeber CeMM
JAC454A
38.60
FL6
F
O
S
O
O
NH
O
O
f1 (ppm)

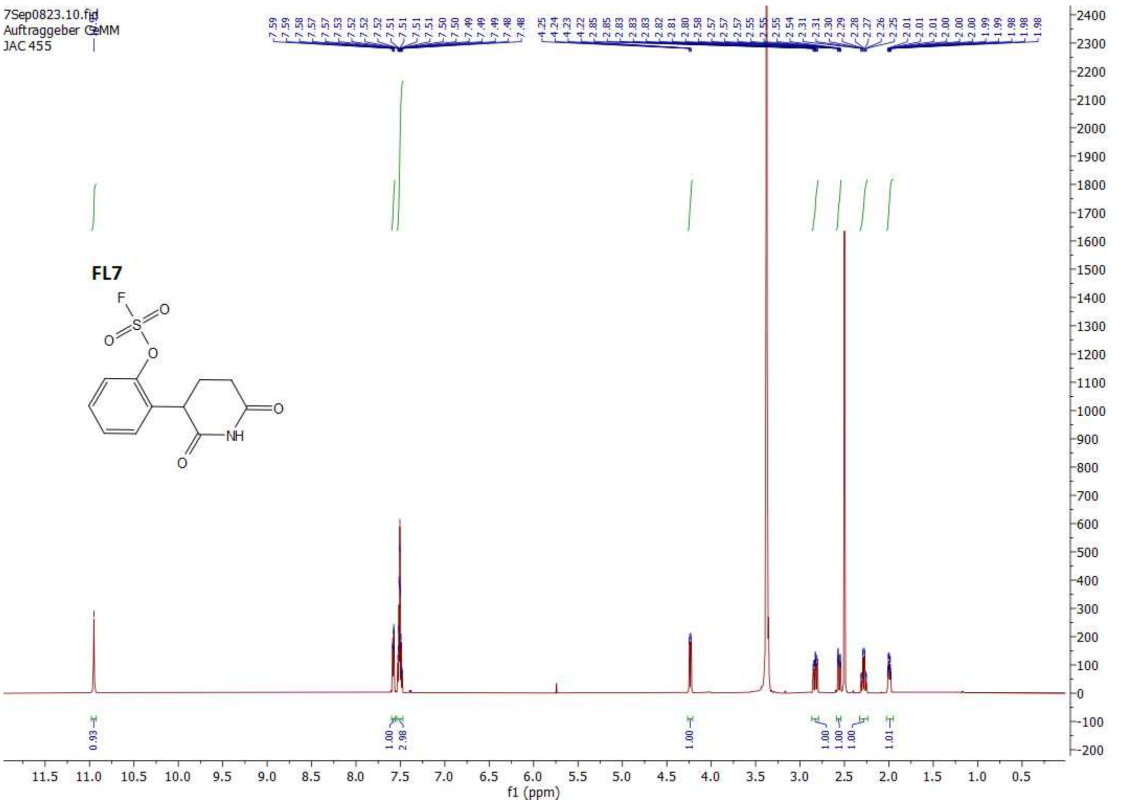
7Sep0823.10.fid
Auftraggeber CeMM
JAC 455
FL7
f1 (ppm)

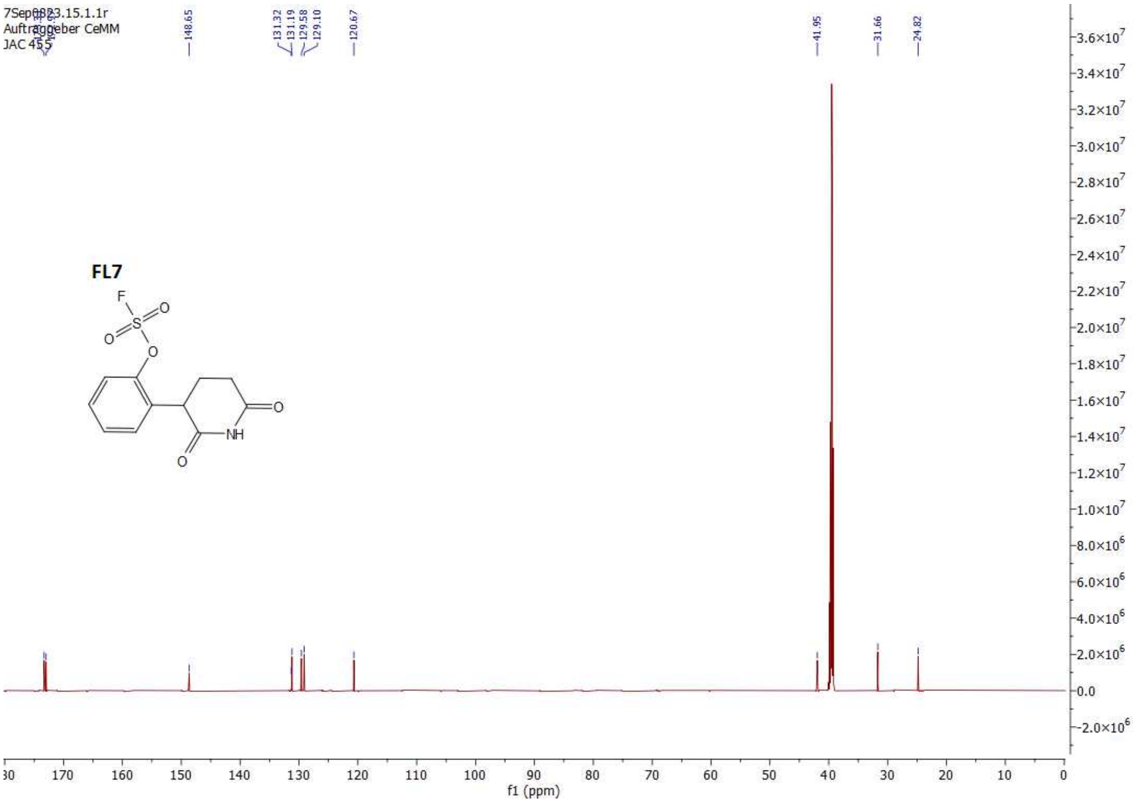
7Sep0823.15.1.1r
Auftraggeber CeMM
JAC 455
148.65
131.32
131.19
129.58
129.10
120.67
41.95
31.66
24.82
FL7
f1 (ppm)

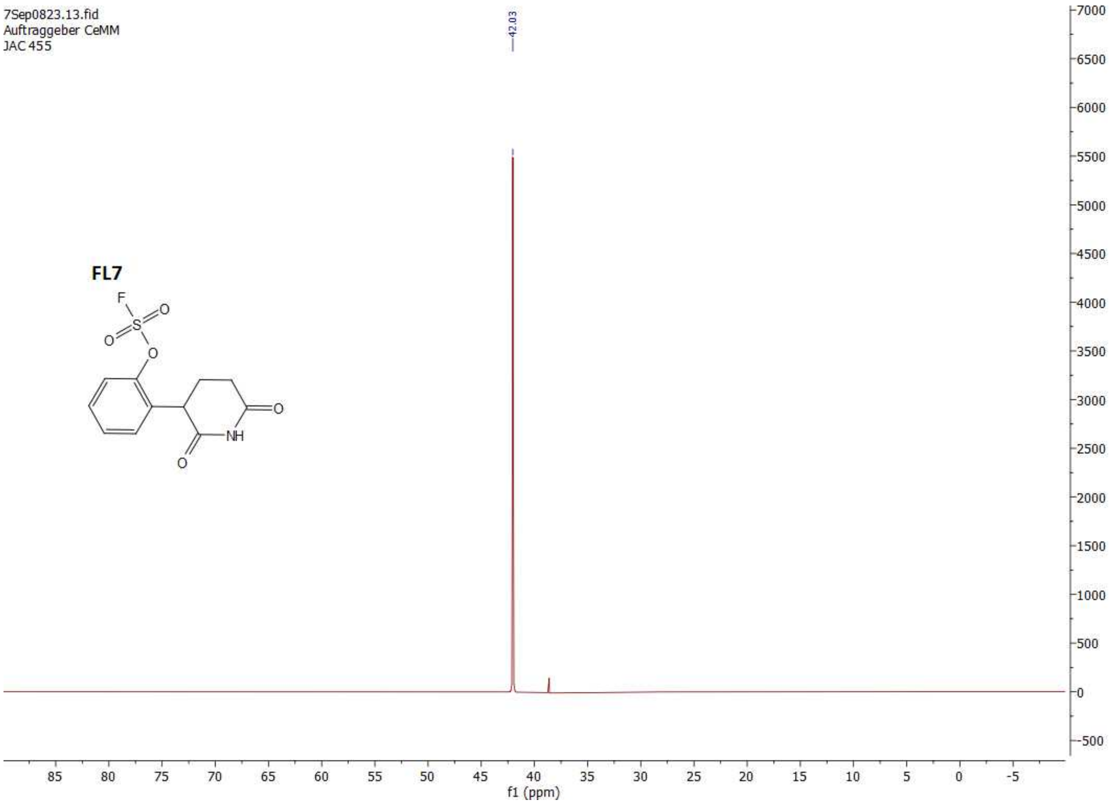
7Sep0823.13.fid
Auftraggeber CeMM
JAC 455
42.03
FL7
f1 (ppm)

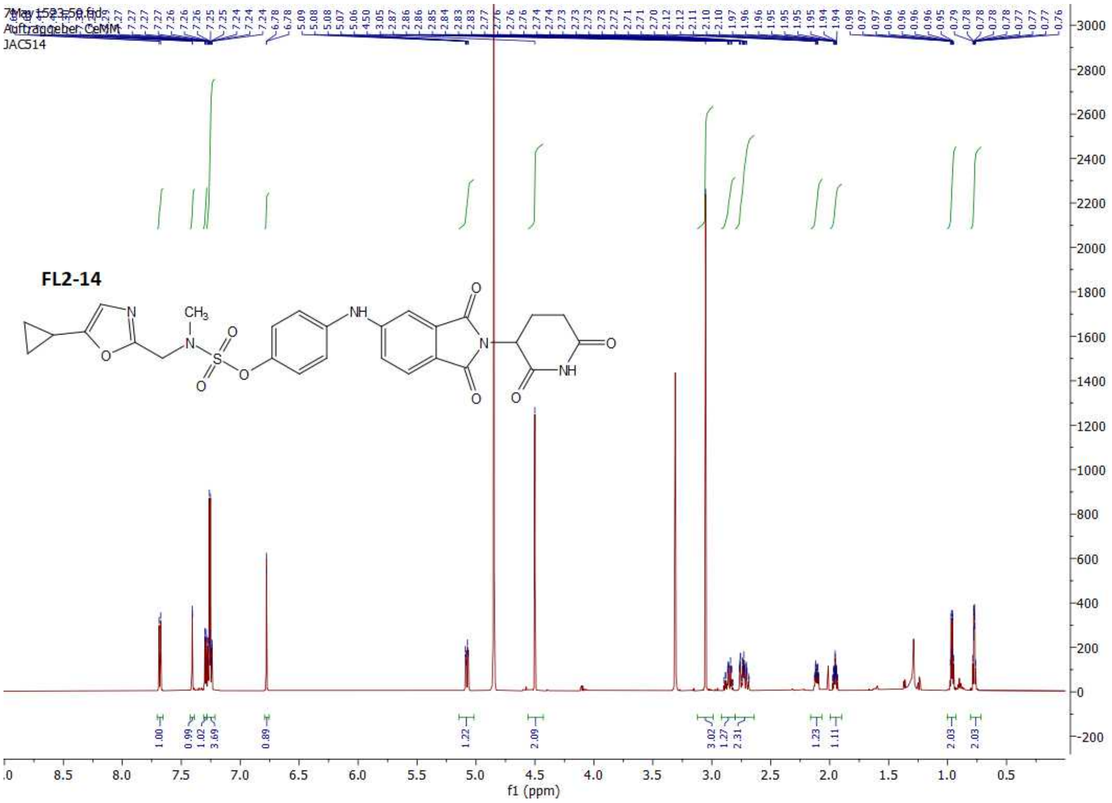
7May1523.50.fid
Auftraggeber CeMM
JAC514
FL2-14
f1 (ppm)

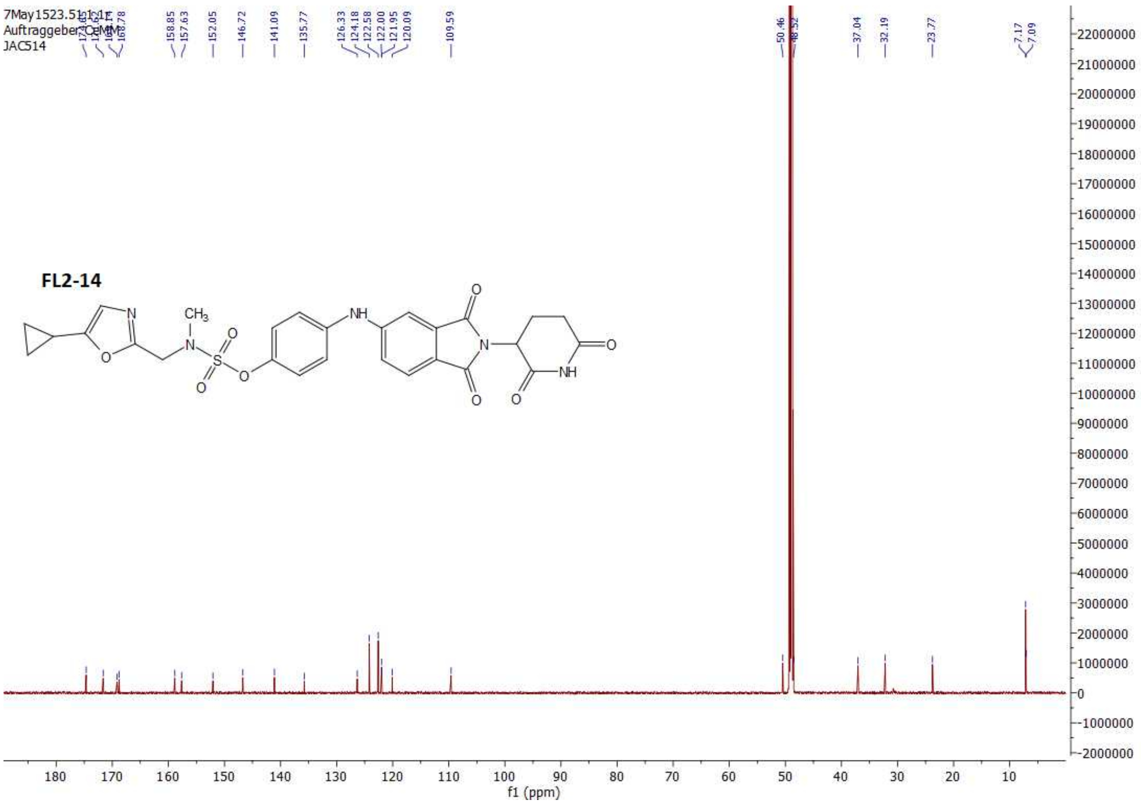
JAC514
168.78
158.85
157.63
152.05
146.72
141.09
135.77
126.33
124.18
122.58
122.00
121.95
120.09
109.59
50.46
48.52
37.04
32.19
23.77
7.17
7.09
FL2-14
f1 (ppm)

Experimental methods

Assessment of gene expression and essentiality in RKO WT

Public datasets on the transcriptome and essentiality of a protein-encoding gene for RKO (cell line ID: ACH-000943) were retrieved from DepMap Public 23Q2 (https://depmap.org/portal/, accessed on 14 August 2023). The public dataset of proteins predicted to harbor the G-loop, the degron recognized by CRBN, was retrieved from the CRBN Substrate Database (https://bailab.siais.shanghaitech.edu.cn/services/crbn-subslib, accessed on 17 May 2023).[5] We retrieved the protein-encoding genes with available transcriptomic and essentiality data (17798 protein-encoding genes). We also filtered the list of predicted CRBN substrates retrieved to those containing the key glycine residue in the seventh position of the G-loop, yielding a list of 2610 CRBN substrates and 2484 of them had transcriptomic and essentiality data. We considered a protein expressed if $\log_2(\text{transcripts per million} + 1) \geq 2$. Expressed proteins were then binned into essential (CERES score ≤ -1) and non-essential (CERES score > -1).

Cell culture

RKO WT, RKO CRBN KO and RKO CRBN OE cells[6] were maintained in Gibco DMEM, high glucose (Fisher-Scientific, #11-965-092) containing 10% fetal calf serum (Thermo-Scientific, #A3382101) and 1% Penicillin-Streptomycin (Sigma-Aldrich, P4333). MV4.11 cells were maintained in RPMI media (Thermo-Scientific, #11530586) with 10% fetal calf serum (Thermo-Scientific #A3382101) and 1% Penicillin-Streptomycin (Sigma-Aldrich, P4333). Cells were cultivated and treated with compounds at 37°C, 5% CO_2 in a humidified cell incubator.

Fluorescence Polarization-based competition assay

Fluorescence Polarization assays were performed in 384-well, Low Volume, Black, Round Bottom, Polystyrene Nonbinding surface Microplates (Corning, #4514). The test compounds were incubated with 200 nM CRBN-DDB1 and 20 nM Pomalidomide-fluorescein tracer **16** in 50 mM Tris-HCl pH 7.5, 200 mM NaCl, 1 mM DTT, 0.02% Triton X-100, and 0.1 µg/µL Bovine Serum Albumin (Sigma Aldrich, #A7030) in a total volume of 25 µL. Fluorescence polarization values were determined with a PerkinElmer Envision reader with FITC FP dual mirror and FITC FP 480 excitation filter and FITC FP 535 emission filters. Fluorescence polarization values of a 10x serial dilution (10 nM to 100 µM) of the test compounds were determined in triplicates. EC50 values were calculated with GraphPad Prism software (version 10.0.2) with a non-linear fit function (4PL sigmoidal curve fit) adjusting the bottom constrain to the FP value of free tracer.

Cell-titer Glo assay

Cell-titer Glo® Luminescent Cell Viability Assays (Promega, #G7572) were performed according to the manufacturer's instructions. Cell viability was determined after 48 h treatment with the drugs. For the screening of the whole SuFEx compound library compound dilution and transfer (8-point dosage, 3-fold dilution, 13.5 µM starting concentration in duplicates) as well as cell seeding of RKO WT cells was carried out by a liquid handling system (PerkinElmer). We used bortezomib as a positive control (13.5 nM). We had 32 negative control wells (DMSO) and 32 positive control wells on each plate. We used these controls to calculate a Z'-factor[7] for each plate individually. All plates had wells with Z' > 0. To compare compounds across plates with different signals, we calculate their signals as a percentage of the control. This calculation is done by linear regression, setting the mean signal of the DMSO wells to 100% and the mean signal of the positive control wells to 0% for each plate. Data visualization was done with *seaborn* (https://seaborn.pydata.org, version 0.12.2).

For the assessment of the cytotoxicity of FL2-14, FL1-2, FL1-12, FL1-18 and CC-885 CellTiter-Glo assays were carried out manually in RKO WT and HEK293T cells (9-point dosage, 3-fold dilution, 10 µM starting concentration in triplicates, 72 h treatment) and data evaluation was done in GraphPad Prism software (version 10.0.2).

Isogenic Cell Painting Assay

The Cell Painting assay we implemented follows the method described by Bray et al.[8] and the updated version described in Cimini et al.[9] with the omission of one cellular stain (SYTO 14) due to limitations of the laser and filter configuration of the Opera Phenix used in this study. We used RKO cell lines (WT, CRBN KO and CRBN OE)[6] which were passaged for no more than 5 passages. When the cells reached a confluency of ca. 80%, they were washed with PBS, detached with trypsin and resuspended in DMEM media. The cell suspension was passed through a 40 µM cell strainer to reduce the clumping of cells. The cells were counted with a CASY Cell Counter and diluted to achieve a count of 1000 cells per well in a volume of 40 µL per well. The cells were dispensed into PhenoPlate 384-well microplates using the Multidrop Combi reagent dispenser (Thermo Scientific #5840300). Microplates were transferred to an incubator at 37 °C, 5.0% (v/v) CO_2, 95.0% humidity for 24 h.

The microplates were than dosed with compound using the Labcyte Echo 550 acoustic liquid handler (Beckman Coulter). We avoided dosing (and imaging) the outermost wells in the microplates as we observed that these wells tended to exhibit imaging biases with lower median channel intensities detected in our imaging set up. The microplates were returned to the incubator for another 48 h.

To stain the cells, we first prepared working solutions of each stain. We diluted Concanavalin A Alexa Fluor 488 conjugate, Phalloidin Alexa Fluor 568 conjugate and MitoTracker Deep Red following the manufacturer's instructions. We diluted Hoechst 33342 with water to a concentration of 20 mg/mL. We prepared the permeabilization buffer comprising 1× HBSS mixed with 0.1% (v/v) Triton X-100 and 1.0% (wt/v) BSA. We mixed the permeabilization buffer with Concanavalin A Alexa Fluor 488 conjugate (f.c. 2 µg/mL), Hoechst 33342 (f.c. 5 µg/mL) and Phalloidin Alexa Fluor 568 conjugate (f.c. 8.25 nM) to obtain a multi-staining and permeabilization solution.

We stained the cells by dispensing 20 µL MitoTracker Deep Red into each well. The microplates were returned to the incubator for 30 min. We then fixed the cells by dispensing 20 µL 16.0% methanol-free paraformaldehyde and incubated the microplates at r.t. for 30 min. We washed the wells with 60 µL PBS 4 times using the BioTek ELX405 washer (Perkin Elmer). We permeabilized and stained the cells using 20 µL multi-staining and permeabilization solution, and incubated the microplates at r.t. for 30 min. We washed off the solution and added 40 µL 1× HBSS with 0.05% (wt/v) NaN_3. We sealed the lid of the microplates with parafilm. The microplates were wrapped with aluminium foil and stored at 4 °C until ready for imaging (for up to four days).

Compound treatment for the isogenic Cell Painting Assay. We used eight compounds that induced diverse morphology (dubbed "morphology controls") as recommended by the JUMP-Cell Painting Consortium.[10] We also curated a list of nine molecular glue degraders and PROTACs with CRBN-dependent bioactivity and seven compounds without CRBN-dependent activity (**Table S3**). In addition to the 132 test compounds synthesized in-plate by sulfur(IV)-fluoride exchange chemistry, we used a total of 156 compounds. The control compounds were present in all microplates used for the Cell Painting assay, while the 132 test compounds were divided across microplates (44 test compounds per plate). The microplates were dosed with compound or DMSO to a final concentration of 10 µM or the equivalent volume to reach the IC_{50} value.

Image acquisition. We acquired images from 5 sites per well using an automated spinning disc confocal HCS device (Perkin Elmer Opera Phenix) equipped with with a 20× 0.4 NA Plan Apochromat air objective (binning 2) and a sCMOS camera. We acquired images in four fluorescent channels (DNA: Ex405/Em435-480; ER: Ex488/Em500-550; AGP: Ex561/Em570-630; Mito: Ex640/Em650-760) and brightfield.

Feature extraction and data pre-processing. We processed and extracted features from the images acquired using *CellProfiler* (https://cellprofiler.org, version 4.2.1) and *cellpose* (https://cellpose.org, version 1.0.2) to extract 3005 morphological features and one feature reflecting cell count. In brief, the illumination correction matrix per channel was calculated for each plate and used for correcting images prior to nuclei and whole cell segmentation and feature extraction. The DNA channel images were used for nuclei segmentation, and the composite images of DNA and AGP channels were used for whole cell segmentation. Segmentation was done using the default models of cellpose (i.e. "nuclei" and "cyto"). The segmentation masks were then used for defining the nuclei, whole cells and cytoplasmic regions for feature extraction using a custom CellProfiler pipeline adapted from the pipeline used by the JUMP-Cell Painting Consortium (https://github.com/broadinstitute/imaging-platform-pipelines/tree/master/JUMP_production).

We assessed the quality of the images with a supervised approach that uses the measurements in the "Image.csv" file from the feature extraction pipeline. To identify biases in cell seeding or staining across the plate, we visualized the cell counts ("Count_RelatedUnfilteredCells") and the median channel intensities of each channel (aggregated per well) as a heatmap formatted to mimic the layout of a 384-well plate and visually inspected them. We also visualized the same features per site as a kernel density estimate plot and rug plot. The plots indicated a skewed distribution in median intensities of the channels with trailing right tails. We, thus, visually inspected images sampled from the trailing right tail of each distribution to determine a suitable threshold on median channel intensities or if thresholding was necessary. The data presented in this study did not require thresholding on the median channel intensities. We also flagged blurry images and images with saturation artefacts by manually setting thresholds on all fluorescent channel measurements with the prefixes "ImageQuality_PowerLogLogSlope" and "ImageQuality_PercentMaximal". We visually inspected flagged images to ensure that they are indeed blurry or contain saturation artefacts. Lastly, we flagged treatments that exhibited an unusually high ratio of nuclei to cells i.e. supernumerary nuclei phenotype. Treatments with ratio within one standard deviation of the ratio for AMG-900 were flagged. AMG-900 is a morphological control that induces the supernumerary nuclei phenotype. All the images and treatments flagged as "failing" our quality checks were recorded in cell-line specific CSV files.

Finally, we assembled the morphological profiles. The individual object CSV files (i.e. "Nuclei.csv", "Cell.csv" and "Cytoplasm.csv") were merged per cell line. Images and treatments flagged during quality control were excluded. The merger of the individual object CSV files assumes each cell has one matching nuclei and cytoplasm object. As such, treatments that induce the supernumerary nuclei must be excluded. Thereafter, the data was aggregated as the median of all detected objects (i.e. cells) per site. Wells with less than three sites and/or 10 cells per site detected in the feature extraction pipeline were excluded. Finally, the cell line-specific profiles were merged, and the cell line context was indicated as "Metadata_Cell".

Feature selection. We employed two strategies for feature selection – the "global" and "treatment-centric" feature selection – using a custom Python class (*SelectFeatures* in post_feature_extraction_modules.py). For both strategies, features that are constant (i.e. they have a median absolute deviation (MAD) of 0) across all treatments or within all DMSO controls in all cell lines are discarded. The remaining features are then selected depending on the strategy employed.

We only carried out global feature selection for RKO WT data, where redundant features are discarded directly. We quantified the median absolute deviation (MAD) of each feature, ordered the features by descending MAD to select features that vary more and removed features with a Pearson's correlation coefficient greater than 0.8. We assumed that features with higher variation could be used to distinguish treatments better than features with lower variation.

The set of global features should, thus, approximate the minimal set of morphological features changed across all treatments.

Treatment-centric feature selection uses data from all cell lines, where an additional step is carried out before redundant features are discarded. For each treatment, we calculated the correlation between feature measurements and cell lines used using Kendall's Tau-b coefficient (*Scientific Python*, https://rdcu.be/b08Wh, version 1.11.1). We treated the cell lines as ordinal data and ranked them as "RKO CRBN KO, RKO CRBN WT and RKO CRBN OE" (in order of increasing CRBN expression). From the Kendall's Tau-b coefficient results, we selected features with a p-value is less than 0.05 after Bonferroni correction for multiple-hypothesis testing. For each cell line, the remaining features are put through a vote on redundancy using a similar process as the global feature selection strategy (ordering the features by descending MAD instead to select features that are more consistent per treatment). Finally, we selected features that are redundant in no more than one cell line. We assume that features that are redundant in more than one cell line are truly redundant, although we could falsely discard non-redundant features in theory. We proceeded with this approach as any redundant features can artificially inflate corrected U scores used for CRBN-dependency prediction (see **Prioritizing Test Compounds by their Likelihood of CRBN-dependent Bioactivity**). The final set of treatment-centric features should, thus, approximate the minimal set of features describing the morphology induced by a compound treatment in a CRBN dependent manner.

The features selected are exported as a CSV file detailing the feature selection strategy used and the respective set of features selected using the strategy.

Two-dimensional UMAP projection of features selected by "global" feature selection for RKO WT. We reduced the features from the 470 selected by the "global" strategy to two UMAP dimensions with *umap-learn* (https://umap-learn.readthedocs.io/en/latest/#, version 0.5.3) with *metric* set to "cosine".

Quantification of strength of morphological perturbation. The strength of morphological perturbation by a compound compared to DMSO controls was calculated using the RKO WT profile and features selected by the "global" strategy (see Feature Selection). We quantified the strength of morphological perturbation as Robust Hellinger Distance following Vuillard et al.[11] We first reduced the features from the 470 selected by the "global" strategy to four UMAP dimensions with *UMAP.jl* (version 0.1.9), using *CosineMetric()* and setting *min_dist* to 2. We then calculated the Robust Hellinger Distance using *BioProfiling.jl*.

Calculating the likelihood that a compound has CRBN-dependent bioactivity. We used the RKO CRBN KO and RKO CRBN OE profiles and features selected by the "treatment-centric" strategy (see Feature Selection) to calculate an "induction score" (relevant custom Python functions in profile_interpretation_modules.py; see GitHub repository). For each treatment, a set of features ($N_{features}$) are selected. The induction score is the average absolute robust Z score per treatment-centric feature (x_i) per image calculated using the following equation:

$$Induction\ score = \sum_{i=1}^{N_{features}} \frac{|x_i|}{N_{features}}$$

As the features selected correlate with CRBN dependency, we hypothesized that compounds that have CRBN-dependent bioactivity should have induction scores greater in the RKO CRBN OE background than in the RKO CRBN KO background. We, thus, compared the induction scores in RKO CRBN OE and RKO CRBN KO using the Mann-Whitney U test (*Scientific Python*, https://rdcu.be/b08Wh, version 1.11.1). The maximum value of U is the product

of the sample sizes of (i.e. number of images in) RKO CRBN OE and RKO CRBN KO data. We, thus, normalized the U scores calculated to allow us to compare U scores for different treatments with varying sample sizes:

$$Corrected\ U = \frac{U}{N_{images,RKO\ CRBN\ KO} \times N_{images,RKO\ CRBN\ OE}}$$

Mathematically, if a compound has a corrected U score > 0.5, the compound has marginally higher induction scores in RKO CRBN OE than in RKO CRBN KO. We considered compounds with corrected U score > 0.5 as potentially CRBN-dependent.

Comparing the treatment-centric features of compounds predicted to have CRBN-dependent bioactivity. We compared the treatment-centric features between all pairs of compounds predicted to have CRBN-dependent bioactivity (number of treatment-centric features ≥ 5 and corrected U scores ≥ 0.7). We first retrieved the set of treatment-centric features selected for the pair of compounds compared. We then retrieve the matrix of Kendall's τ-b correlation coefficients calculated per feature for each compound. In our use case, τ approximates the strength of positive or negative correlation between the robust Z scores of a feature with the CRBN-expression level of cell lines. We finally compare the matrices of τ values between compounds using the cosine similarity metric (*Numerical Python*, https://numpy.org, version 1.24.3).

We then visualized the cosine similarities calculated on a network graph with *NetworkX* (https://networkx.org, version 3.1), using the *spring_layout()* with seed set to 500 and iterations set to 200. Cosine similarities ≥ 0.85 were represented as thick edges with a *weight* of 3.0, while those < 0.85 were represented as light grey edges with a *weight* of 0.2. Thick edges were also color-coded. If any compound/node connected by the edge is a CRBN-dependent control, the edge is blue. Otherwise, the edge is black

Expression proteomics to identify the degradation target of FL2-14

Sample preparation. Quantitative proteomics was performed for an unbiased, proteome-wide identification of the degradation target(s) of FL2-14 in RKO cells. 40 million RKO cells per condition were treated with selected SuFEx IMiDs for 16 hours in biological duplicates. Cells were harvested via centrifugation, washed three times in ice-cold PBS and snap-frozen in liquid nitrogen. Each washed cell pellet was lysed separately in 500 µl of freshly prepared lysis buffer containing 50 mM HEPES (pH 8.0), 2% SDS, 1 mM PMSF and protease inhibitor cocktail (Sigma-Aldrich). Samples rested at RT for 20 minutes before heating to 99°C for 5 min. After cooling down to RT, DNA was sheared by sonication using a Covaris S2 high performance ultrasonicator. Cell debris was removed by centrifugation at 16,000 × g for 15 min at 20°C. Supernatant was transferred to fresh Eppendorf tubes and protein concentration determined using the BCA protein assay kit (Pierce Biotechnology). Filter-aided sample preparation (FASP) was performed using a 30 kDa molecular weight cutoff centrifugal filters (Microcon 30, Ultracel YM-30, Merck Millipore) essentially according to the procedure described by Wisniewski et al.[12] In brief, 100 µg of total protein per sample was reduced by the addition of DTT to a final concentration of 83.3 mM, followed by incubation at 99°C for 5 minutes. After cooling to room temperature, samples were mixed with 200 µL of freshly prepared 8 M urea in 100 mM Tris-HCl (pH 8.5) (UA-solution) in the filter unit and centrifuged at 14.000 × g for 15 min at 20°C to remove SDS. Residual SDS was washed out by a second wash step with 200 µL UA solution. Proteins were alkylated with 100 µl of 50 mM iodoacetamide in the dark for 30 min at RT. Thereafter, three washes were performed with 100 µL of UA solution, followed by three washes with 100 µL of 50 mM TEAB buffer (Sigma-Aldrich). Proteolytic digestion is performed using the protease trypsin in a 1:50 ratio overnight at 37°C. Peptides were recovered using 40 µL of 50 mM TEAB buffer followed by 50 µL of 0.5 M NaCl (Sigma-Aldrich). Peptides were desalted using the Thermo Scientific™ Pierce™

Peptide Desalting Spin Columns (Pierce). TMTpro 18plex Label Reagent Set was used for labeling according to the manufacturer (Pierce). After the labeling reaction was quenched, the samples were pooled, the organic solvent removed in the vacuum concentrator, and the labeled peptides purified by C18 solid phase extraction (SPE).

Tryptic peptides were re-buffered in 10 mM ammonium formate buffer pH 10, shortly before separation by reversed phase (RP) liquid chromatography at pH 10 as described by Gilar et al.[13] Peptides were separated into 96 time-based fractions on a Phenomenex C18 RP column (150 × 2.0 mm Gemini-NX, 3 µm C18 110Å, Phenomenex, Torrance, CA, USA) using Dionex Ultimate 3000 series HPLC system fitted with a binary pump delivering solvent at 50 µL/min. Acidified fractions were consolidated into 36 fractions via a concatenated strategy described by Wang et al.[14] After removal of solvent in a vacuum concentrator, samples were reconstituted in 0.1% TFA prior to LC-MS/MS analysis.

Sample analysis (LC-MS/MS). Mass spectrometry analysis was performed on an Orbitrap Fusion Lumos Tribrid mass spectrometer (ThermoFisher Scientific) coupled to a Dionex Ultimate 3000 RSLCnano system (ThermoFisher Scientific) via a Nanospray Flex Ion Source (ThermoFisher Scientific) interface. Peptides were loaded onto a PepMap 100 C18, 5 µm, 5 × 0.3 mm trap column (ThermoFisher Scientific) at a flow rate of 10 µL/min using 0.1% TFA as loading buffer. After loading, the trap column was switched in-line with an Acclaim PepMap nanoHPLC C18 analytical column with 2.0 µm particle size and a dimension of 75 µm IDx 500 mm (ThermoFisher Scientific, #164942). The column temperature was maintained at 50°C. Mobile phase A consisted of 0.4% formic acid in water, and mobile phase B consisted of 0.4% formic acid in a mixture of 90% acetonitrile and 10% water. Separation was achieved using a multistep gradient over 150 min at a flow rate of 230 nL/min (increase of initial gradient from 6% to 9% solvent B within 1 min, 9% to 30% solvent B within 146 min, 30% to 65% solvent B within 8 min, 65% to 100% solvent B within of 1 minute and 100% solvent B for 6 minutes before equilibrating to 6% solvent B for 24 minutes before the next injection). In the liquid junction setup, electrospray ionization was enabled by applying a voltage of 1.8 kV directly to the liquid being sprayed, and non-coated silica emitter was used.

The mass spectrometer was operated in a data-dependent acquisition mode (DDA) and used a synchronous precursor selection (SPS) approach. For both MS2 and MS3 levels, we collected a 400–1600 m/z survey scan in the Orbitrap at 120 000 resolution (FTMS1), the AGC target was set to 'standard' and a maximum injection time (IT) of 50 ms was applied. Precursor ions were filtered by charge state (2-5), dynamic exclusion (60 s with a ±10 ppm window), and monoisotopic precursor selection. Precursor ions for data-dependent MSn (ddMSn) analysis were selected using 10 dependent scans (TopN approach). A charge-state filter was used to select precursors for data-dependent scanning. In ddMS2 analysis, spectra were obtained using one charge state per branch (from z=2 to z=5) in a dual-pressure linear ion trap (ITMS2). The quadrupole isolation window was set to 0.7 Da and the collision-induced dissociation (CID) fragmentation technique was used at a normalized collision energy of 35%. The normalized AGC target was set to 200% with a maximum IT of 35 ms. During the ddMS3 analyses, precursors were isolated using SPS waveform and different MS1 isolation windows (1.3 m/z for z=2, 1.2 m/z for z=3, 0.8 m/z for z= 4 and 0.7 m/z for z = 5). Target MS2 fragment ions were further fragmented by high-energy collision induced dissociation (HCD) followed by Orbitrap analysis (FTMS3). The normalized HCD collision energy was set to 45% and the normalized AGC target was set to 300% with a maximum IT of 100 ms. The resolution was set to 50 000 with a defined scanning range of 100 to 500 m/z. Xcalibur Version 4.3.73.11 and Tune 3.4.3072.18 were used to operate the instrument.

Data processing and analysis. Following data acquisition, the acquired raw data files were processed using the Proteome Discoverer v.2.4.1.15 platform, with a TMT18plex quantification method selected. In the processing step, we used the Sequest HT database search engine and the Percolator validation software node to remove false positives with a false discovery rate (FDR) of 1% at the peptide and protein level under stringent conditions. All MSn

spectra were searched against the human proteome (Canonical, reviewed, 20 304 sequences) and appended known contaminants and streptavidin, with a maximum of two allowable miscleavage sites. The search was performed with full tryptic digestion. Methionine oxidation (+15.994 Da) and protein N-terminal acetylation (+42.011 Da), as well as methionine loss (-131.040 Da) and protein N-terminal acetylation with methionine loss (-89.030 Da) were set as variable modifications, while carbamidomethylation (+57.021 Da) of cysteine residues and tandem mass tag (TMT) 18-plex labeling of peptide N termini and lysine residues (+304.207 Da) were set as fixed modifications. Data were searched with mass tolerances of ±10 ppm and ±0.6 Da for the precursor and fragment ions, respectively. Results were filtered to include peptide spectrum matches with Sequest HT cross-correlation factor (Xcorr) scores of ≥1 and high peptide confidence assigned by Percolator. MS2 signal-to-noise (S/N) values of TMTpro reporter ions were used to calculate peptide/protein abundance values. Peptide spectrum matches (PSMs) with precursor isolation interference values of > 70, average TMTpro reporter ion S/N < 10 and SPS Mass Matches < 65 % were excluded from quantification. Both unique and razor peptides were used for TMT quantification. Correction of isotopic impurities was applied. Data were normalized to total peptide abundance to correct for experimental bias and scaled “to all average”. Protein ratios are directly calculated from the grouped protein abundances using an ANOVA hypothesis test. Adjusted p-values are calculated using the Benjamini-Hochberg method.

Analysis of targeted protein degradation of GSPT1 and GSPT2 via western blot

For degradation assays 800 000 RKO WT or CRBN KO cells were seeded 1 day prior to the treatment in 6 well plates. Then, cells were treated with 10 µM of the compounds for 16 hours if not otherwise stated. Cells were harvested, lysed for 30 min at 37°C in lysis buffer (1x PBS, 1% SDS, 2 mM $MgCl_2$, 1x Halt Protease inhibitor cocktail, 50 Units Benzonase) and cleared for 30 min at 18 000 x g at 4°C. Protein concentration was determined with the Pierce 660 nm Protein Assay using the Ionic Detergent Compatibility Reagent (Thermo Scientific). 10 µg total proteome were subjected to western blot analysis by adding 4x Bolt™ LDS Sample Buffer (Thermo Scientific). Samples were boiled at 95°C for 5 min and subjected to gel electrophoresis with Bolt™ 4-12% Bis-Tris Plus Gels (Thermo Scientific) with Bolt™ MES SDS Running Buffer (Thermo Scientific). Membranes were blocked with 5% fat-free milk in TBS-T (20 mM Tris, 150 mM NaCl, 0.1% Tween, pH 7.6) for 1 h at room temperature and washed subsequently 3x 5 min with TBS-T. Afterwards, they were incubated for 1 h at room temperature with either GSPT1 antibody (Abcam ab49878, rabbit, 1:1000), GSPT2 antibody (Thermo #PA5-60824, rabbit, 1:1000), IKZF1 (Cell Signaling Technologies, CST14859S, rabbit, 1:10 000), CRBN (Cell Signaling Technologies, CST71810S, rabbit, 1:1000), or GAPDH (SantaCruz sc-47724, mouse, 1:1 000). Followed by washing 3x 5 min with TBS-T and incubation for 1 h at room temperature with either Goat ANTI-RABBIT IgG StarBright™ Blue 700 (Bio-Rad, #12004162, 1:10 000) or Goat anti-Mouse IgG (H+L) Cross-Adsorbed Secondary Antibody, DyLight™ 800 (Bio-Rad, STAR117D800GA, 1:10 000) fluorophore-conjugated secondary antibodies. Following washing 3x 5 min with TBS-T, images were acquired with a ChemiDoc system (Bio-Rad).

Prediction of the G-loop degron sequence in GSPT2

We first compiled all CRBN degron sequences with known crystal structures (**Table S 2**). We subsequently discarded 6UML as its degron sequence included an alanine instead of the typical glycine observed in the seventh amino acid residue of other degron sequences, leaving six degron sequences. We then scanned the latest AlphaFold structure of GSPT2[15] for a potential stretch of 10 amino acids that would align best with each degron sequence. We yielded the same stretch of amino acid residues from position 560 to 569 (LVDKKSGEKS) and RMSD values under 0.5 Å.

Table S 2: List of crystal structures used for the computational prediction of the G-loop degron sequence in GSPT2

PDB code	Resolution (Å)	Chain	Segment	Sequence	Reference
5FQD	2.45	C	34-43	AINITN**G**EEV	16
6UML	3.58	C	389-398	AQCKIC**A**SHI	17
5HXB	3.60	A	569-578	LVDKKS**G**EKS	18
6XK9	3.64	A	568-577	LVDKKS**G**EKS	19
6H0F	3.25	C	145-154	FQCNQC**G**ASF	20
6H0G	4.25	C	417-426	LQCEIC**G**FTC	20
7BQU	1.90	B	410-419	FVCSVC**G**HRF	21

Analysis of G-loop mutation

GSPT2 in a pENTR223 vector was obtained from the BCCM/GeneCorner ORFeome (https://bccm.belspo.be/, #81108-A10) and cloned in a lentiviral backbone containing a C-terminal 2xHA-tag by Gateway cloning. The Invitrogen Clonase Gateway LR Clonase II Enzyme Mix (Thermo Fisher, #11-791-020) was used according to the manufacturer's instructions. The construct was amplified in *E. coli* Stbl3 cells at 30°C over night and DNA was extracted with the QIAGEN Plasmid Plus Midi Kit (Qiagen, #12943).

The G566N point mutation was introduced in the pENTR-GSPT2 vector using the Q5 site-directed mutagenesis kit (NEB, #E0552S) with forward primer (CAAAAAATCAaacGAAAAAAGTAAGACACGAC) and reverse primer (TCTACCAAGGAGATTAAC) according to the manufacturer's instructions. For the PCR reaction an initial denaturation step at 98°C was followed by 30 cycles of 10 sec at 98°C, 20 sec at 55°C and 120 sec at 72°C and a final extension of 2 min at 72°C. The PCR product was treated with KLD mix (NEB, #M0554S) according to the manufacturer's instructions. The plasmid was amplified in *E. coli* Stbl3 cells, the mutation was verified by Sanger sequencing and the modified insert was cloned in the same HA-tag destination vector as wild-type GSPT2 as described above.

For generation of lentivirus of the two constructs, Lenti-X 293T cells (at approx. 80% confluency) were co-transfected with the target vector, lentiviral psPAX2 helper (Addgene #12260) and pMD2.G envelope (Addgene #12259) using polyethyleneimine (PEI MAX® MW 40 000, Polysciences, #24765-100) as previously described.[22] Viral supernatant was harvested after 60 h and cleared of cellular debris by filtration through a 0.45-µm PES filter. Cells were transduced with respective virus via spinfection (900 x g, 1 h, 37 °C) in the presence of Polybrene (6.4 µg/mL, SantaCruz, SC-134220). Cells were selected with Puromycin (1 µg/mL, Gibco, A1113803) two days post transduction for a total of 5 days, after which the expression of target proteins was validated via western blotting with an anti-HA-tag antibody (CST #3724S, rabbit, 1:10 000).

For degradation assays 800 000 RKO wt or CRBN knock-out cells were seeded 1 day prior to the treatment in 6 well plates. Cells were treated with 10 µM of the compounds for 16 hours if not otherwise stated. Cells were harvested, lysed as described above and 10 µg total proteome were subjected to western blot analysis as described above.

Membranes were incubated for 1 h at room temperature with either anti-HA-tag antibody (CST #3724S, rabbit, 1:10 000) or GAPDH (SantaCruz sc-47724, mouse, 1:1 000). Followed by washing 3x 5 min with TBS-T and incubation for 1 h at room temperature with either Goat ANTI-RABBIT IgG StarBright™ Blue 700 (Bio-Rad, #12004162, 1:10 000) or Goat anti-Mouse IgG (H+L) Cross-Adsorbed Secondary Antibody, DyLight™ 800 (Bio-Rad, STAR117D800GA, 1:10 000) fluorophore-conjugated secondary antibodies. Following washing 3x 5 min with TBS-T, images were acquired with a ChemiDoc system (Bio-Rad).

Expression and purification of recombinant CRBN-DDB1 complex

Baculovirus expression vector systems (BEVS) for His-CRBN and His-DDB1ΔB (lacking beta-propeller B) were generated with the BestBac system kit (Expression Systems, BioTrend #91-200) in SF9 cells according to the manufacturer's instructions. The utilized vectors pESF140_HisSpy_DDB1ΔB and pAJ075_hsCRBN were a gift from Eric Fischer (Addgene plasmids #124213 and #124214). BEVS were propagated in SF9 cells to obtain high titer stocks. For gene expression High Five insect cells were harvested, carefully resuspended with a 1:1 ratio of DDB1 and CRBN BEVS solutions and incubated for 60 min at 27 °C and 120 rpm shaking. Afterwards, cells were resuspended in pre-warmed SF-900 media and distributed to sterile plastic flasks with vented lids. Cells were harvested after 72 h and cell pellets were stored at -20°C. Next, cell pellets were resuspended in lysis buffer (50 mM Tris pH 7.5, 200 mM NaCl, 0.5% Triton X-100, 1 mM DTT, 1x HALT protease inhibitors, 1x DNAse I, 2.5 mM $MgCl_2$, 0.1 mM CaCl2), incubated for 30 min on ice and sonicated (2 cycles with 30 sec, 30% output level, Branson Sonifier). Crude lysates were centrifugated for 30 min with 18 000 x g at 4°C. In the meantime, Ni-NTA agarose bead slurry was washed twice with Wash buffer 1 (50 mM Tris pH 7.5, 100 mM NaCl), the cleared lysate was added, and beads were incubated for 1 h on a roller at 4°C to allow binding. Next, beads were washed three times with Wash buffer 1 (50 mM Tris pH 7.5, 100 mM NaCl) followed by three washes with Wash buffer 2 (50 mM Tris pH 7.5, 100 mM NaCl, 10 mM Imidazole, 1 mM DTT). Next, the CRBN-DDB1 protein complex was eluted five times with Elution buffer (50 mM Tris pH 7.5, 100 mM NaCl, 250 mM Imidazole, 1 mM DTT, 1x HALT protease inhibitors). Fractions containing proteins of interest were united and subjected to anion exchange chromatography with a HiTrap Q HP 1 mL column using an ÄKTA FPLC system. Proteins were concentrated and buffer was exchanged to Storage buffer (50 mM Tris pH 7.5, 100 mM NaCl, 1 mM DTT) with Amicon filter devices with an Ultracel-30 regenerated cellulose membrane (Sigma-Aldrich, #UFC803008). Proteins were snap-frozen in liquid nitrogen and stored at -80°C.

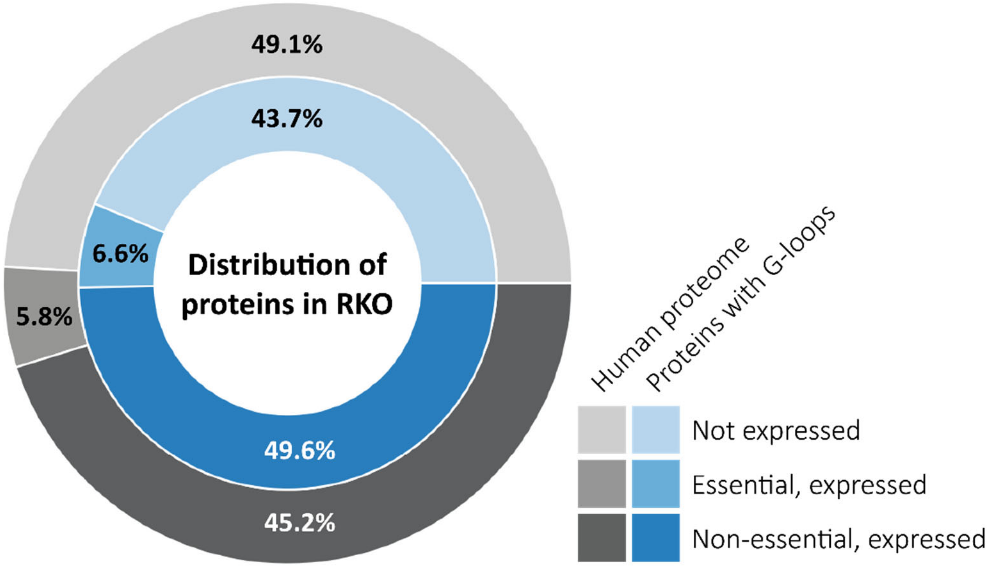

Figure S1: Distribution of proteins in RKO (the cell line background primarily used in this study) classified by expression (presumed from transcription levels) and essentiality. A total of 17798 protein-encoding genes have transcriptomic and essentiality data available publicly from DepMap (https://depmap.org/portal/). RKO expressed slightly over half of the proteins comprising the human proteome (ignoring protein isoforms encoded by the same gene). A tenth of the expressed proteome is essential, while the remainder of the expressed proteome is non-essential. Reflecting a similar trend, out of the 2484 proteins predicted to contain a G-loop (the degron recognized by CRBN)[5] with publicly available transcriptomic and essentiality data, over half are expressed in RKO. A large proportion of these G-loop containing proteins are non-essential and expressed in RKO.

Table S3: List of compounds used as CRBN-dependent (in blue) and CRBN-independent (in pink) controls in the isogenic CPA and their reported targets. Transcriptomics and essentiality data retrieved from DepMap Public 23Q2 files (OmicsExpressionProteinCodingGenesTPMLogp1.csv and CRISPRGeneDependency.csv respectively) for RKO WT (DepMap ID: ACH-000943). Targets with a log_2TPM ≥ 2 were considered as “expressed” and targets with a gene dependency score ≤ -1 were considered as “essential”.

**Compound (mechanism) with reference(s)**	**Absent from public transcriptomics/ essentiality data**	**Not expressed**	**Expressed, not essential**	**Expressed, essential**
Avadomide[23] (MGD)		IKZF1, IKZF3	ZFP91	
Pomalidomide[20,24–28] (MGD)	ZFP692, WIZ1	IKZF1, IKZF3, ZNF653, ZNF827, SALL4, ZNF98, ZBTB16	ZFP91, ZNF276, RNF166, GZF1, ZBTB39, FAM83F, RAB28, DTWD1	
Lenalidomide[20,25,26,28–30] (MGD)	ZFP692, WIZ1	IKZF1, IKZF3, ZNF653, ZNF827, SALL4	ZFP91, ZNF276, RNF166, CSNK1A1, FAM83F, RAB28	
Iberdomide (CC-220)[25,31] (MGD)		IKZF1, IKZF3, ZNF98	ZFP91	
CC-885[18,19] (MGD)		IKZF1, IKZF3	GSPT2, CSNK1A1, HBS1L	GSPT1
CC-90009[19] (MGD)				GSPT1
TL 13-12[32] (PROTAC)		ALK, FER	PTK2	AURKA
dBET1[33] (PROTAC)			BRD2, BRD3	BRD4
dBET6[34] (PROTAC)			BRD2, BRD3	BRD4
Palbociclib[35] (Inhibitor)			CDK4, CDK6	
MZ1[36] (PROTAC)			BRD2, BRD3	BRD4
Cis/MZ1[36] (Inactive PROTAC/ Inhibitor)			BRD2, BRD3	BRD4
ARV-771[37] (PROTAC)			BRD2, BRD3	BRD4
dCeMM2[38] (MGD)				CCNK
Indisulam[39] (MGD)			RBM23	RBM39
Methylated pomalidomide[40] (Inactive MGD)				

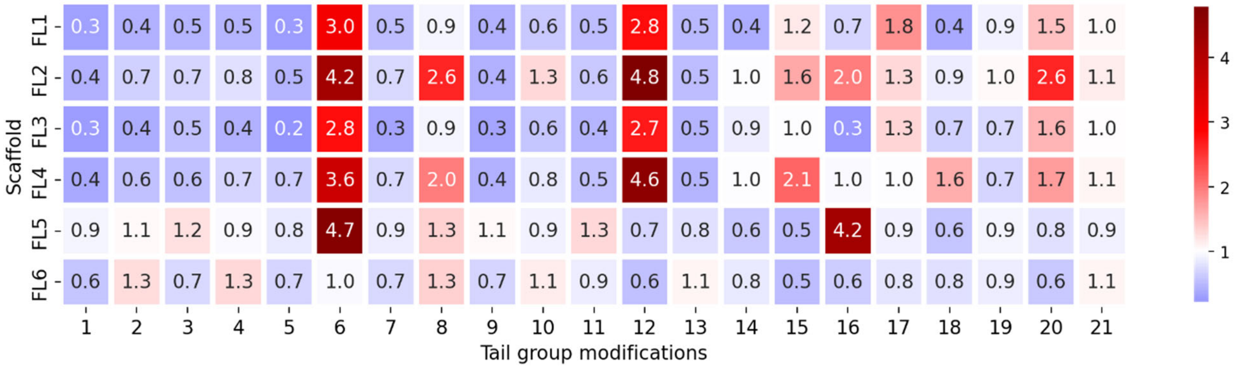

Figure S2: CRBN binding affinities of the library synthesized using the SuFEx protocol reported in this study. The CRBN binding affinities of these compounds were determined with the Fluorescence Polarization-based competition assay described in the methods section. The values are given in µM and are the average of triplicate measurements.

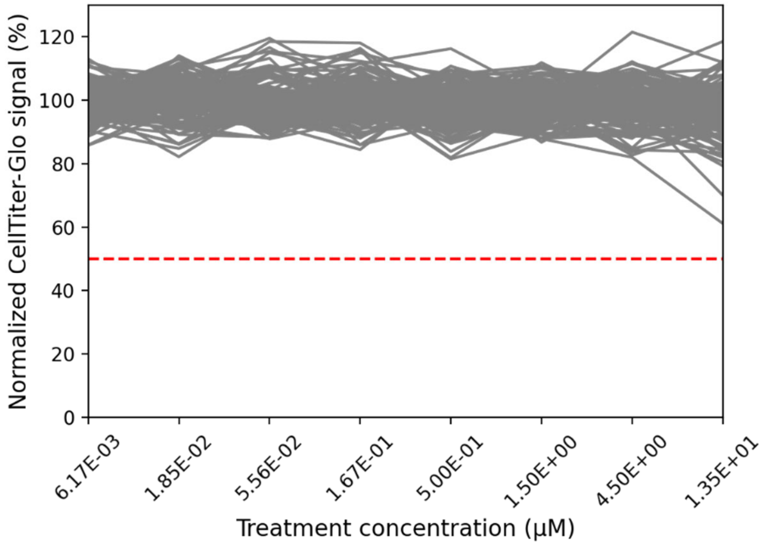

Figure S 3: Assessment of cytotoxicity of CRBN binders in RKO WT using CellTiter-Glo assays (Promega). None of the CRBN binders reduced the normalized Cell-Titer-Glo signal to 50% (marked in red). This observation indicates that none of the CRBN binders induce cytotoxic effects up to treatment concentrations of 13.5 µM. Only two compounds induce mild cytotoxic effects with normalized CellTiter-Glo signals quantified slightly under 65.0% at the maximum treatment concentration of 13.5 µM. The normalized CellTiter-Glo signal quantified is the average of duplicate measurements normalized to a positive control (bortezomib-treated cells) and a negative control (DMSO-treated cells).

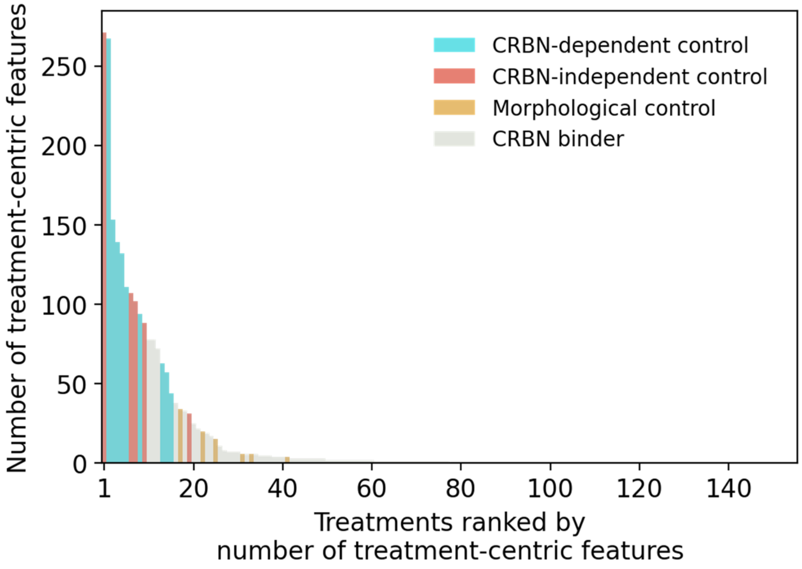

Figure S 4: Treatment-centric features can vary greatly across compounds. Some CRBN-independent controls have numbers of treatment-centric features similar (or even more than) that of CRBN-dependent controls.

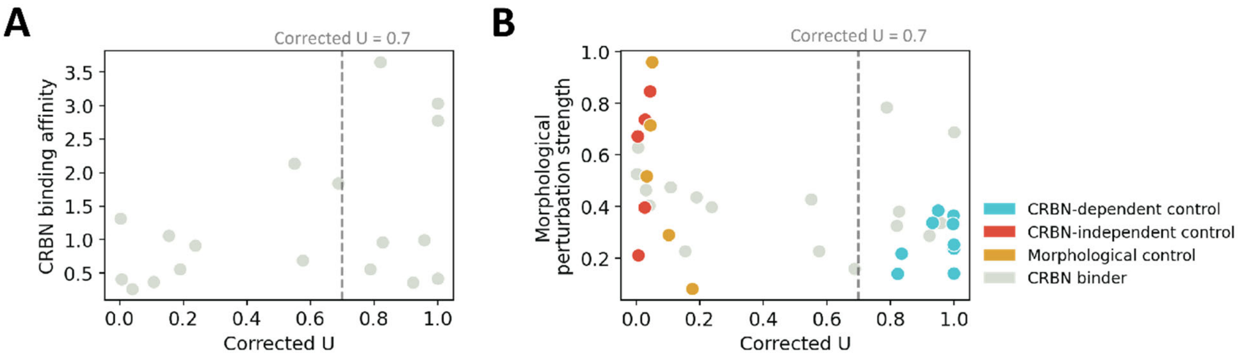

Figure S 5: The corrected U score calculated does not correlate with the CRBN binding affinity or morphological perturbation strength of the compound. (A) The CRBN binding affinity of CRBN binders (given in µM, also shown in Figure S2) compared to their corrected U scores. (B) The morphological perturbation strength of all compounds compared to their corrected U scores.

Table S 4: List of compounds with at least five treatment-centric features and corrected U scores ≥ 0.7 i.e. they are predicted to have CRBN-dependent bioactivity. The compounds colored in blue are CRBN-dependent controls, while the ones in black are CRBN binders. The CRBN binding affinities shown were quantified by the fluorescence polarization-based competition assay (data also shown in Figure S2).

Compound	Number of treatment-centric features	Corrected U score	CRBN binding affinity (EC_{50})
Avadomide	57	0.823333	
CC-885	132	1	
CC-90009	44	1	
dBET1	63	1	
dBET6	94	0.997222	
Iberdomide	153	0.932778	
Lenalidomide	139	0.950556	
Pomalidomide	111	0.835392	
TL 13-12	267	0.99887	
**FL1-12**	78	1	2.776
**FL1-18**	33	0.9225	0.363
**FL1-2**	72	1	0.419
**FL1-6**	78	1	3.034
**FL2-14**	25	0.9575	0.991
**FL4-2**	8	0.7875	0.557
**FL4-6**	22	0.82	3.65
**FL6-6**	6	0.8275	0.961

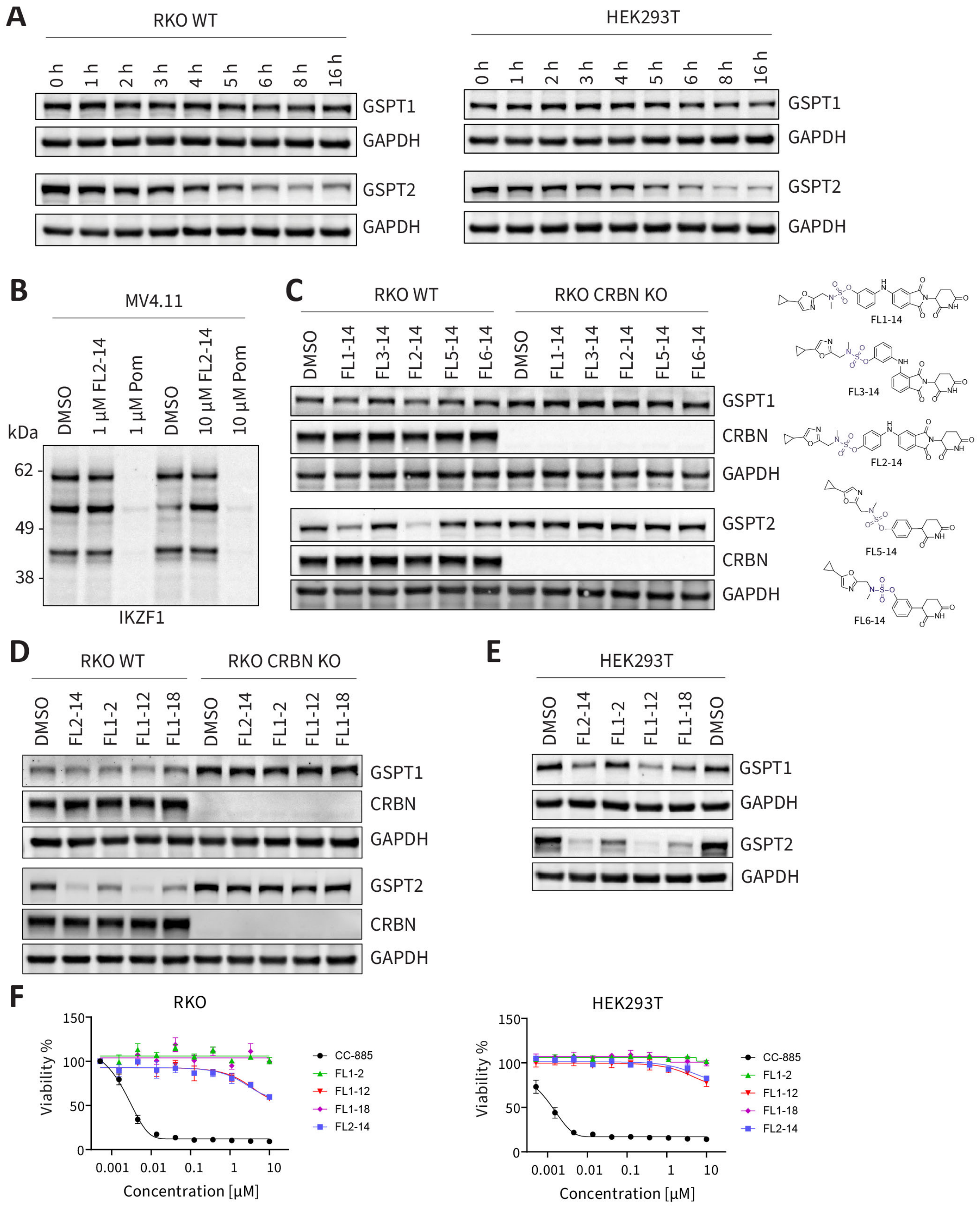

Figure S 6: Additional validation experiments of GSPT1 and GSPT2 degradation by FL2-14. (A) Time-course of treatment of RKO and HEK293T cells with 10 µM FL2-14. (B) Degradation of IKZF1 in MV4.11 cells. Cells were treated for 16 h with the indicated concentrations of compounds. Pom: Pomalidomide. (C) Analysis of CRBN binders with the tail group modification **14** from the library of CRBN binders. RKO WT or RKO CRBN KO cells were treated for 16 h with 10 µM of the indicated compounds. The chemical structures of tested compounds are shown below the Western blot images. (D and E) Analysis of compounds with

treatment-centric features similar to FL2-14 in **Figure 2E** in two cell line backgrounds namely RKO and HEK293T. RKO WT, RKO CRBN KO and HEK293T cells were treated for 16 h with 10 µM of the indicated compounds. (F) Analysis of the cytotoxicity of compounds with treatment-centric features related to FL2-14 in RKO and HEK293T cells after treatment with up to 10 µM of the compounds for 72 h.

Supplementary references

(1) Bricelj, A.; Dora Ng, Y. L.; Ferber, D.; Kuchta, R.; Müller, S.; Monschke, M.; Wagner, K. G.; Krönke, J.; Sosič, I.; Gütschow, M.; Steinebach, C. Influence of Linker Attachment Points on the Stability and Neosubstrate Degradation of Cereblon Ligands. *ACS Med. Chem. Lett.* **2021**, *12* (11), 1733–1738. https://doi.org/10.1021/acsmedchemlett.1c00368.

(2) Zhou, Q.; Reekie, T. A.; Abbassi, R. H.; Indurthi Venkata, D.; Font, J. S.; Ryan, R. M.; Munoz, L.; Kassiou, M. Synthesis and in Vitro Evaluation of Diverse Heterocyclic Diphenolic Compounds as Inhibitors of DYRK1A. *Bioorg. Med. Chem.* **2018**, *26* (22), 5852–5869. https://doi.org/10.1016/j.bmc.2018.10.034.

(3) Yeung, S. Y.; Kampmann, S.; Stubbs, K. A.; Skelton, B. W.; Kaskow, B. J.; Abraham, L. J.; Stewart, S. G. Novel Thalidomide Analogues with Potent NFκB and TNF Expression Inhibition. *Medchemcomm* **2011**, *2* (11), 1073. https://doi.org/10.1039/c1md00184a.

(4) Zhang, J.; Che, J.; Luo, X.; Wu, M.; Kan, W.; Jin, Y.; Wang, H.; Pang, A.; Li, C.; Huang, W.; Zeng, S.; Zhuang, W.; Wu, Y.; Xu, Y.; Zhou, Y.; Li, J.; Dong, X. Structural Feature Analyzation Strategies toward Discovery of Orally Bioavailable PROTACs of Bruton's Tyrosine Kinase for the Treatment of Lymphoma. *J. Med. Chem.* **2022**, *65* (13), 9096–9125. https://doi.org/10.1021/acs.jmedchem.2c00324.

(5) Wang, L.; Li, F. L.; Ma, X. Y.; Cang, Y.; Bai, F. PPI-Miner: A Structure and Sequence Motif Co-Driven Protein-Protein Interaction Mining and Modeling Computational Method. *J. Chem. Inf. Model.* **2022**, *62* (23), 6160–6171. https://doi.org/10.1021/acs.jcim.2c01033.

(6) Hanzl, A.; Casement, R.; Imrichova, H.; Hughes, S. J.; Barone, E.; Testa, A.; Bauer, S.; Wright, J.; Brand, M.; Ciulli, A.; Winter, G. E. Functional E3 Ligase Hotspots and Resistance Mechanisms to Small-Molecule Degraders. *Nat. Chem. Biol.* **2023**, *19* (3), 323–333. https://doi.org/10.1038/s41589-022-01177-2.

(7) Zhang, J.-H.; Chung, T. D. Y.; Oldenburg, K. R. A Simple Statistical Parameter for Use in Evaluation and Validation of High Throughput Screening Assays. *SLAS Discov.* **1999**, *4* (2), 67–73. https://doi.org/10.1177/108705719900400206.

(8) Bray, M. A.; Singh, S.; Han, H.; Davis, C. T.; Borgeson, B.; Hartland, C.; Kost-Alimova, M.; Gustafsdottir, S. M.; Gibson, C. C.; Carpenter, A. E. Cell Painting, a High-Content Image-Based Assay for Morphological Profiling Using Multiplexed Fluorescent Dyes. *Nat. Protoc.* **2016**, *11* (9), 1757–1774. https://doi.org/10.1038/nprot.2016.105.

(9) Cimini, B. A.; Chandrasekaran, S. N.; Kost-Alimova, M.; Miller, L.; Goodale, A.; Fritchman, B.; Byrne, P.; Garg, S.; Jamali, N.; Logan, D. J.; Concannon, J. B.; Lardeau, C.-H.; Mouchet, E.; Singh, S.; Shafqat Abbasi, H.; Aspesi, P.; Boyd, J. D.; Gilbert, T.; Gnutt, D.; Hariharan, S.; Hernandez, D.; Hormel, G.; Juhani, K.; Melanson, M.; Mervin, L. H.; Monteverde, T.; Pilling, J. E.; Skepner, A.; Swalley, S. E.; Vrcic, A.; Weisbart, E.; Williams, G.; Yu, S.; Zapiec, B.; Carpenter, A. E. Optimizing the Cell Painting Assay for Image-Based Profiling. *Nat. Protoc.* **2023**, *18* (July). https://doi.org/10.1038/s41596-023-00840-9.

(10) Singh, S.; Chandrasekaran, S. N.; Carpenter, A. E.; Cimini, B. A.; Arevalo, J. *JUMP-Target*. GitHub. https://github.com/jump-cellpainting/JUMP-Target#positive-control-compounds.

(11) Vulliard, L.; Hancock, J.; Kamnev, A.; Fell, C. W.; Ferreira Da Silva, J.; Loizou, J. I.; Nagy, V.; Dupre, L.; Menche,

J. BioProfiling.Jl: Profiling Biological Perturbations with High-Content Imaging in Single Cells and Heterogeneous Populations. *Bioinformatics* **2022**, *38* (6), 1692–1699. https://doi.org/10.1093/bioinformatics/btab853.

(12) Wiśniewski, J. R.; Zougman, A.; Nagaraj, N.; Mann, M. Universal Sample Preparation Method for Proteome Analysis. *Nat. Methods* **2009**, *6* (5), 359–362. https://doi.org/10.1038/nmeth.1322.

(13) Gilar, M.; Olivova, P.; Daly, A. E.; Gebler, J. C. Two-dimensional Separation of Peptides Using RP-RP-HPLC System with Different PH in First and Second Separation Dimensions. *J. Sep. Sci.* **2005**, *28* (14), 1694–1703. https://doi.org/10.1002/jssc.200500116.

(14) Wang, Y.; Yang, F.; Gritsenko, M. A.; Wang, Y.; Clauss, T.; Liu, T.; Shen, Y.; Monroe, M. E.; Lopez-Ferrer, D.; Reno, T.; Moore, R. J.; Klemke, R. L.; Camp, D. G.; Smith, R. D. Reversed-Phase Chromatography with Multiple Fraction Concatenation Strategy for Proteome Profiling of Human MCF10A Cells. *Proteomics* **2011**, *11* (10), 2019–2026. https://doi.org/10.1002/pmic.201000722.

(15) Varadi, M.; Anyango, S.; Deshpande, M.; Nair, S.; Natassia, C.; Yordanova, G.; Yuan, D.; Stroe, O.; Wood, G.; Laydon, A.; Zídek, A.; Green, T.; Tunyasuvunakool, K.; Petersen, S.; Jumper, J.; Clancy, E.; Green, R.; Vora, A.; Lutfi, M.; Figurnov, M.; Cowie, A.; Hobbs, N.; Kohli, P.; Kleywegt, G.; Birney, E.; Hassabis, D.; Velankar, S. AlphaFold Protein Structure Database: Massively Expanding the Structural Coverage of Protein-Sequence Space with High-Accuracy Models. *Nucleic Acids Res.* **2022**, *50* (D1), D439–D444. https://doi.org/10.1093/nar/gkab1061.

(16) Petzold, G.; Fischer, E. S.; Thomä, N. H. Structural Basis of Lenalidomide-Induced CK1α Degradation by the CRL4 CRBN Ubiquitin Ligase. *Nature* **2016**, *532* (7597), 127–130. https://doi.org/10.1038/nature16979.

(17) Matyskiela, M. E.; Clayton, T.; Zheng, X.; Mayne, C.; Tran, E.; Carpenter, A.; Pagarigan, B.; McDonald, J.; Rolfe, M.; Hamann, L. G.; Lu, G.; Chamberlain, P. P. Crystal Structure of the SALL4–Pomalidomide–Cereblon–DDB1 Complex. *Nat. Struct. Mol. Biol.* **2020**, *27* (4), 319–322. https://doi.org/10.1038/s41594-020-0405-9.

(18) Matyskiela, M. E.; Lu, G.; Ito, T.; Pagarigan, B.; Lu, C. C.; Miller, K.; Fang, W.; Wang, N. Y.; Nguyen, D.; Houston, J.; Carmel, G.; Tran, T.; Riley, M.; Nosaka, L.; Lander, G. C.; Gaidarova, S.; Xu, S.; Ruchelman, A. L.; Handa, H.; Carmichael, J.; Daniel, T. O.; Cathers, B. E.; Lopez-Girona, A.; Chamberlain, P. P. A Novel Cereblon Modulator Recruits GSPT1 to the CRL4 CRBN Ubiquitin Ligase. *Nature* **2016**, *535* (7611), 252–257. https://doi.org/10.1038/nature18611.

(19) Surka, C.; Jin, L.; Mbong, N.; Lu, C. C.; Jang, I. S.; Rychak, E.; Mendy, D.; Clayton, T.; Tindall, E.; Hsu, C.; Fontanillo, C.; Tran, E.; Contreras, A.; Ng, S. W. K.; Matyskiela, M.; Wang, K.; Chamberlain, P.; Cathers, B.; Carmichael, J.; Hansen, J.; Wang, J. C. Y.; Minden, M. D.; Fan, J.; Pierce, D. W.; Pourdehnad, M.; Rolfe, M.; Lopez-Girona, A.; Dick, J. E.; Lu, G. CC-90009, a Novel Cereblon E3 Ligase Modulator, Targets Acute Myeloid Leukemia Blasts and Leukemia Stem Cells. *Blood* **2021**, *137* (5), 661–677. https://doi.org/10.1182/blood.2020008676.

(20) Sievers, Q. L.; Petzold, G.; Bunker, R. D.; Renneville, A.; Słabicki, M.; Liddicoat, B. J.; Abdulrahman, W.; Mikkelsen, T.; Ebert, B. L.; Thomä, N. H. Defining the Human C2H2 Zinc Finger Degrome Targeted by Thalidomide Analogs through CRBN. *Science (80-.).* **2018**, *362* (6414). https://doi.org/10.1126/science.aat0572.

(21) Furihata, H.; Yamanaka, S.; Honda, T.; Miyauchi, Y.; Asano, A.; Shibata, N.; Tanokura, M.; Sawasaki, T.; Miyakawa, T. Structural Bases of IMiD Selectivity That Emerges by 5-Hydroxythalidomide. *Nat. Commun.* **2020**, *11* (1), 1–11. https://doi.org/10.1038/s41467-020-18488-4.

(22) Joung, J.; Konermann, S.; Gootenberg, J. S.; Abudayyeh, O. O.; Platt, R. J.; Brigham, M. D.; Sanjana, N. E.; Zhang, F. Genome-Scale CRISPR-Cas9 Knockout and Transcriptional Activation Screening. *Nat. Protoc.* **2017**,

12 (4), 828–863. https://doi.org/10.1038/nprot.2017.016.

(23) Hagner, P. R.; Man, H.-W.; Fontanillo, C.; Wang, M.; Couto, S.; Breider, M.; Bjorklund, C.; Havens, C. G.; Lu, G.; Rychak, E.; Raymon, H.; Narla, R. K.; Barnes, L.; Khambatta, G.; Chiu, H.; Kosek, J.; Kang, J.; Amantangelo, M. D.; Waldman, M.; Lopez-Girona, A.; Cai, T.; Pourdehnad, M.; Trotter, M.; Daniel, T. O.; Schafer, P. H.; Klippel, A.; Thakurta, A.; Chopra, R.; Gandhi, A. K. CC-122, a Pleiotropic Pathway Modifier, Mimics an Interferon Response and Has Antitumor Activity in DLBCL. *Blood* **2015**, *126* (6), 779–789. https://doi.org/10.1182/blood-2015-02-628669.

(24) An, J.; Ponthier, C. M.; Sack, R.; Seebacher, J.; Stadler, M. B.; Donovan, K. A.; Fischer, E. S. PSILAC Mass Spectrometry Reveals ZFP91 as IMiD-Dependent Substrate of the CRL4CRBN Ubiquitin Ligase. *Nat. Commun.* **2017**, *8* (1), 15398. https://doi.org/10.1038/ncomms15398.

(25) Donovan, K. A.; An, J.; Nowak, R. P.; Yuan, J. C.; Fink, E. C.; Berry, B. C.; Ebert, B. L.; Fischer, E. S. Thalidomide Promotes Degradation of SALL4, a Transcription Factor Implicated in Duane Radial Ray Syndrome. *Elife* **2018**, *7*. https://doi.org/10.7554/eLife.38430.

(26) Krönke, J.; Udeshi, N. D.; Narla, A.; Grauman, P.; Hurst, S. N.; McConkey, M.; Svinkina, T.; Heckl, D.; Comer, E.; Li, X.; Ciarlo, C.; Hartman, E.; Munshi, N.; Schenone, M.; Schreiber, S. L.; Carr, S. A.; Ebert, B. L. Lenalidomide Causes Selective Degradation of IKZF1 and IKZF3 in Multiple Myeloma Cells. *Science (80-.).* **2014**, *343* (6168), 301–305. https://doi.org/10.1126/science.1244851.

(27) Matyskiela, M. E.; Zhu, J.; Baughman, J. M.; Clayton, T.; Slade, M.; Wong, H. K.; Danga, K.; Zheng, X.; Labow, M.; LeBrun, L.; Lu, G.; Chamberlain, P. P.; Thompson, J. W. Cereblon Modulators Target ZBTB16 and Its Oncogenic Fusion Partners for Degradation via Distinct Structural Degrons. *ACS Chem. Biol.* **2020**, *15* (12), 3149–3158. https://doi.org/10.1021/acschembio.0c00674.

(28) Yu, H. H.; Reitsma, J. M.; Sweredoski, M. J.; Moradian, A.; Hess, S.; Deshaies, R. J. Single Subunit Degradation of WIZ, a Lenalidomide- and Pomalidomide-Dependent Substrate of E3 Ubiquitin Ligase CRL4CRBN. *bioRxiv* **2019**.

(29) Krönke, J.; Fink, E. C.; Hollenbach, P. W.; MacBeth, K. J.; Hurst, S. N.; Udeshi, N. D.; Chamberlain, P. P.; Mani, D. R.; Man, H. W.; Gandhi, A. K.; Svinkina, T.; Schneider, R. K.; McConkey, M.; Järås, M.; Griffiths, E.; Wetzler, M.; Bullinger, L.; Cathers, B. E.; Carr, S. A.; Chopra, R.; Ebert, B. L. Lenalidomide Induces Ubiquitination and Degradation of CK1α in Del(5q) MDS. *Nature* **2015**, *523* (7559), 183–188. https://doi.org/10.1038/nature14610.

(30) Lu, G.; Middleton, R. E.; Sun, H.; Naniong, M. V.; Ott, C. J.; Mitsiades, C. S.; Wong, K. K.; Bradner, J. E.; Kaelin, W. G. The Myeloma Drug Lenalidomide Promotes the Cereblon-Dependent Destruction of Ikaros Proteins. *Science (80-.).* **2014**, *343* (6168), 305–309. https://doi.org/10.1126/science.1244917.

(31) Matyskiela, M. E.; Zhang, W.; Man, H. W.; Muller, G.; Khambatta, G.; Baculi, F.; Hickman, M.; Lebrun, L.; Pagarigan, B.; Carmel, G.; Lu, C. C.; Lu, G.; Riley, M.; Satoh, Y.; Schafer, P.; Daniel, T. O.; Carmichael, J.; Cathers, B. E.; Chamberlain, P. P. A Cereblon Modulator (CC-220) with Improved Degradation of Ikaros and Aiolos. *J. Med. Chem.* **2018**, *61* (2), 535–542. https://doi.org/10.1021/acs.jmedchem.6b01921.

(32) Powell, C. E.; Gao, Y.; Tan, L.; Donovan, K. A.; Nowak, R. P.; Loehr, A.; Bahcall, M.; Fischer, E. S.; Jänne, P. A.; George, R. E.; Gray, N. S. Chemically Induced Degradation of Anaplastic Lymphoma Kinase (ALK). *J. Med. Chem.* **2018**, *61* (9), 4249–4255. https://doi.org/10.1021/acs.jmedchem.7b01655.

(33) Winter, G. E.; Buckley, D. L.; Paulk, J.; Roberts, J. M.; Souza, A.; Dhe-Paganon, S.; Bradner, J. E. Phthalimide Conjugation as a Strategy for in Vivo Target Protein Degradation. *Science (80-.).* **2015**, *348* (6241), 1376–1381. https://doi.org/10.1126/science.aab1433.

(34) Winter, G. E.; Mayer, A.; Buckley, D. L.; Erb, M. A.; Roderick, J. E.; Vittori, S.; Reyes, J. M.; di Iulio, J.; Souza, A.; Ott, C. J.; Roberts, J. M.; Zeid, R.; Scott, T. G.; Paulk, J.; Lachance, K.; Olson, C. M.; Dastjerdi, S.; Bauer, S.; Lin, C. Y.; Gray, N. S.; Kelliher, M. A.; Churchman, L. S.; Bradner, J. E. BET Bromodomain Proteins Function as Master Transcription Elongation Factors Independent of CDK9 Recruitment. *Mol. Cell* **2017**, *67* (1), 5-18.e19. https://doi.org/10.1016/j.molcel.2017.06.004.

(35) Bollard, J.; Miguela, V.; Ruiz de Galarreta, M.; Venkatesh, A.; Bian, C. B.; Roberto, M. P.; Tovar, V.; Sia, D.; Molina-Sánchez, P.; Nguyen, C. B.; Nakagawa, S.; Llovet, J. M.; Hoshida, Y.; Lujambio, A. Palbociclib (PD-0332991), a Selective CDK4/6 Inhibitor, Restricts Tumour Growth in Preclinical Models of Hepatocellular Carcinoma. *Gut* **2017**, *66* (7), 1286–1296. https://doi.org/10.1136/gutjnl-2016-312268.

(36) Zengerle, M.; Chan, K. H.; Ciulli, A. Selective Small Molecule Induced Degradation of the BET Bromodomain Protein BRD4. *ACS Chem. Biol.* **2015**, *10* (8), 1770–1777. https://doi.org/10.1021/acschembio.5b00216.

(37) Raina, K.; Lu, J.; Qian, Y.; Altieri, M.; Gordon, D.; Rossi, A. M. K.; Wang, J.; Chen, X.; Dong, H.; Siu, K.; Winkler, J. D.; Crew, A. P.; Crews, C. M.; Coleman, K. G. PROTAC-Induced BET Protein Degradation as a Therapy for Castration-Resistant Prostate Cancer. *Proc. Natl. Acad. Sci. U. S. A.* **2016**, *113* (26), 7124–7129. https://doi.org/10.1073/pnas.1521738113.

(38) Mayor-Ruiz, C.; Bauer, S.; Brand, M.; Kozicka, Z.; Siklos, M.; Imrichova, H.; Kaltheuner, I. H.; Hahn, E.; Seiler, K.; Koren, A.; Petzold, G.; Fellner, M.; Bock, C.; Müller, A. C.; Zuber, J.; Geyer, M.; Thomä, N. H.; Kubicek, S.; Winter, G. E. Rational Discovery of Molecular Glue Degraders via Scalable Chemical Profiling. *Nat. Chem. Biol.* **2020**, *16* (11), 1199–1207. https://doi.org/10.1038/s41589-020-0594-x.

(39) Han, T.; Goralski, M.; Gaskill, N.; Capota, E.; Kim, J.; Ting, T. C.; Xie, Y.; Williams, N. S.; Nijhawan, D. Anticancer Sulfonamides Target Splicing by Inducing RBM39 Degradation via Recruitment to DCAF15. *Science (80-.).* **2017**, *356* (6336). https://doi.org/10.1126/science.aal3755.

(40) Lu, J.; Qian, Y.; Altieri, M.; Dong, H.; Wang, J.; Raina, K.; Hines, J.; Winkler, J. D.; Crew, A. P.; Coleman, K.; Crews, C. M. Hijacking the E3 Ubiquitin Ligase Cereblon to Efficiently Target BRD4. *Chem. Biol.* **2015**, *22* (6), 755–763. https://doi.org/10.1016/j.chembiol.2015.05.009.
